# Cartilage-selective genes identified in genome-scale analysis of non-cartilage and cartilage gene expression

**DOI:** 10.1186/1471-2164-8-165

**Published:** 2007-06-12

**Authors:** Vincent A Funari, Allen Day, Deborah Krakow, Zachary A Cohn, Zugen Chen, Stanley F Nelson, Daniel H Cohn

**Affiliations:** 1 Medical Genetics Institute, Cedars-Sinai Medical Center, SSB-3, 8700 Beverly Blvd, Los Angeles, CA 90048, USA; 2 Departments of Human Genetics, David Geffen School of Medicine at UCLA, Los Angeles, CA 90095, USA; 3 Departments of Obstetrics and Gynecology, David Geffen School of Medicine at UCLA, Los Angeles, CA 90095, USA; 4 Departments of Pediatrics, David Geffen School of Medicine at UCLA, Los Angeles, CA 90095, USA

## Abstract

**Background:**

Cartilage plays a fundamental role in the development of the human skeleton. Early in embryogenesis, mesenchymal cells condense and differentiate into chondrocytes to shape the early skeleton. Subsequently, the cartilage anlagen differentiate to form the growth plates, which are responsible for linear bone growth, and the articular chondrocytes, which facilitate joint function. However, despite the multiplicity of roles of cartilage during human fetal life, surprisingly little is known about its transcriptome. To address this, a whole genome microarray expression profile was generated using RNA isolated from 18–22 week human distal femur fetal cartilage and compared with a database of control normal human tissues aggregated at UCLA, termed Celsius.

**Results:**

161 cartilage-selective genes were identified, defined as genes significantly expressed in cartilage with low expression and little variation across a panel of 34 non-cartilage tissues. Among these 161 genes were cartilage-specific genes such as cartilage collagen genes and 25 genes which have been associated with skeletal phenotypes in humans and/or mice. Many of the other cartilage-selective genes do not have established roles in cartilage or are novel, unannotated genes. Quantitative RT-PCR confirmed the unique pattern of gene expression observed by microarray analysis.

**Conclusion:**

Defining the gene expression pattern for cartilage has identified new genes that may contribute to human skeletogenesis as well as provided further candidate genes for skeletal dysplasias. The data suggest that fetal cartilage is a complex and transcriptionally active tissue and demonstrate that the set of genes selectively expressed in the tissue has been greatly underestimated.

## Background

Skeletogenesis begins with condensation of mesenchymal chondroprogenitor cells to form the cartilage anlagen that pattern the early skeleton. Subsequently, for bones that grow by endochondral ossification, the chondrocytes differentiate further to establish the growth plates. At the joint surfaces, development of articular cartilage facilitates and maintains joint movement during fetal life. These multi-step processes require the coordinated expression of many genes, including genes encoding extracellular matrix proteins and morphogens, as well as proliferative, angiogenic, and apoptotic signals [1]. Most of our knowledge of the function of the genes involved has been derived from developmental studies in model systems and cell lines [2] as well as from the identification of disease genes in skeletal disorders.

Whole genome analysis of chondrocyte gene expression has the potential to reveal novel genes and gene expression programs which define the tissue. Although the complete set of genes expressed in human cartilage has not yet been described, analysis of human cartilage cDNA libraries has provided an initial *in vivo *picture of the cartilage transcriptome [3-6]. These investigations have also identified expression of both known and novel genes. Comparative microarray studies in rat cartilage [7] and several chondrocyte cell lines [8,9] have provided a larger set of genes of potential importance in chondrocytes, including genes specific to the stages of chondrocyte differentiation. Wang et al. (2004) identified 92 genes with two-fold variation in expression between hypertrophic and proliferative growth plate chondrocytes. In this *in vivo *study, significant gene expression changes were principally associated with cell cycle, transcription, extracellular matrix structure, receptor and transporter functions. In microarray studies of mouse micromass cultures [8], 212 genes exhibited at least a ten-fold difference in gene expression as the cultures differentiated. Thus global characterization of gene expression is beginning to describe the identities of key regulatory molecules and their targets in chondrocytes.

Disrupting genes involved in the organization and maturation of the growth plate and/or the stability of articular cartilage results in inherited skeletal disorders that range from perinatal lethal phenotypes to mild disorders with early-onset osteoarthropathy as their major feature [10,11]. Of the approximately 370 clinically distinguishable skeletal dysplasias [12], mutations in 115 genes have been associated with about 150 disorders. Many of these disease genes are expressed in a cartilage-selective pattern, and therefore identifying additional genes uniquely expressed in cartilage should yield new skeletal dysplasia candidate genes.

To identify a larger set of genes uniquely expressed in chondrocytes, this study describes a genome-scale gene expression profile for 18–22 week human fetal cartilage. There were 161 genes which appeared to be selectively expressed in fetal cartilage, comprising a variety of novel genes that may contribute to skeletal development. The data suggest a complex pattern of cartilage gene expression and indicate that the number of genes selectively expressed in cartilage has been greatly underestimated.

## Results

### Identification of cartilage-selective genes

To define a set of genes preferentially or uniquely expressed in normal human fetal cartilage, cartilage probeset intensities were compared with probeset intensities across a variety of normal tissues. A two-step process was employed for gene identification, consisting of a training step and a validation step (see the additional data file 1, for a flow chart of an overview of the analysis). The tissue-selectivity of a representative sampling of the identified genes was confirmed by quantitative RT-PCR.

The training dataset consisted of five independent cartilage samples and 41 non-cartilage samples, all analyzed using Affymetrix U133 2.0 Plus arrays. The average correlation coefficient among the cartilage samples (R^2^) was 0.96. To identify unbiased relationships within the data, and to test the robustness of the normalization and tissue-specificity, an unsupervised approach [13], in which the genes and tissues were grouped based only on expression patterns, was employed. Probesets with the greatest variation across all tissues and whose expression in any two arrays differed by at least two standard deviations from their mean expression across the entire set of samples were selected. This selection yielded 9483 probesets.

**Figure 2:**
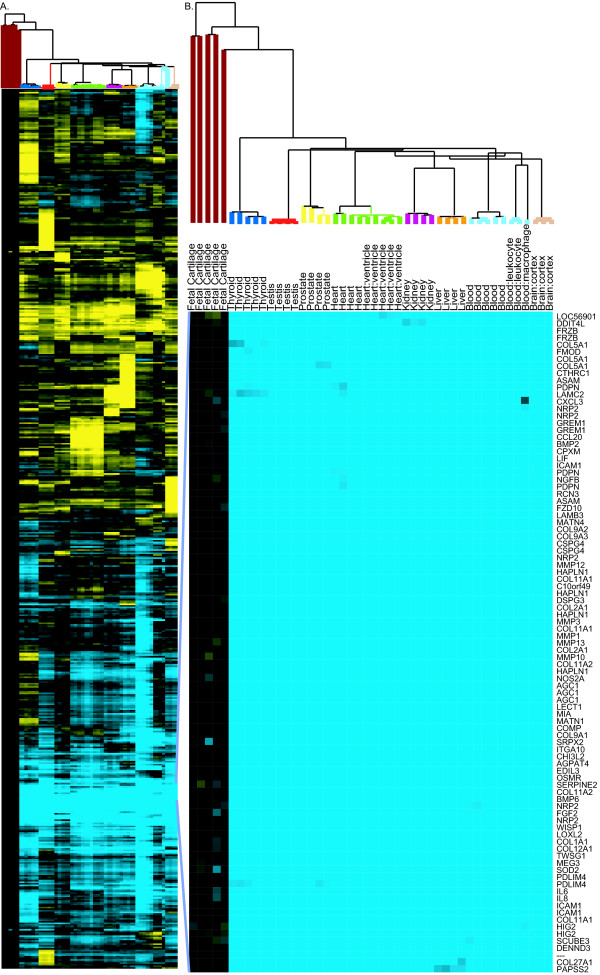
**Unsupervised analysis of all tissues using U133 2.0 microarrays**. (A) Two-way hierarchical clustering of 46 normal tissues (including five fetal cartilage and 41 non-cartilage tissues) and 9483 probesets which vary more than two standard deviations from the mean expression of the probeset. As detailed in the Methods, to generate a cartilage-centric figure, the median cartilage signals were normalized to zero prior to the clustering. This resulted in cartilage gene expression being displayed to the left and expression among the non-cartilage tissues to the right and produced deep branching among the cartilage samples. Mean-centered clustering reflected the high correlation among the cartilage samples (average regression correlation of 0.92) and did not qualitatively alter the correlations among the non-cartilage samples (data not shown). (B) Zoomed image of gene expression from 89 probesets representing 64 genes containing many known cartilage-specific genes which distinguish fetal cartilage expression.

Two-way hierarchical clustering based on similarity of expression of these 9483 probesets within the samples was performed (Figure 2). Samples from the same tissues clustered together, indicating that the normalization was sufficiently robust to allow tissue-selective expression patterns to be identified. Even with these relatively non-stringent selection criteria, the results showed a surprisingly large number of genes with a fetal cartilage-selective expression pattern. At least 89 probesets representing 64 genes with coordinately higher expression in cartilage relative to non-cartilage tissues appeared to drive the clustering of the two groups (Figure 2B). These probesets formed a gene expression node in the dendrogram which shared an overall expression correlation of 0.99. The genes represented by these probesets included some well established cartilage-selective genes, including aggrecan (*AGC1*), type × collagen (*COL10A1*), and matrilin 3 (*MATN3*), among others. Thus, a comparative approach with microarrays can identify genes whose expression is cartilage-selective.

To define a ranked list of genes significantly expressed in cartilage, a supervised analysis [13], comparing cartilage versus non-cartilage gene expression, was employed. This consisted of a two-class analysis with a modified t-test (SAM) (See additional data file 2, for the complete results of this analysis). There were 2634 probesets representing 1720 genes with at least three-fold differential expression when comparing cartilage and non-cartilage tissues, with a false discovery rate of zero. Of these, 2446 of the 2634 probesets demonstrated higher expression in cartilage with respect to non-cartilage tissues, while the remaining 188 probesets were expressed at significantly higher levels in the other tissues. As observed for the hierarchical clustering, probesets representing well-known cartilage markers, including *COL2A1*, *AGC1*, *COMP*, *COL9A3*, and *MMP3 *were among the top genes listed. In addition, lubricin (*PRG4*), an articular cartilage-specific marker, was also identified, confirming the ability to identify genes specific to fetal articular cartilage. Indeed, among the top 35 probesets more highly expressed in cartilage, only four probesets, representing unannotated genes, were derived from genes not previously known to be expressed in cartilage.

### In silico validation

Three array platforms were used to validate the 2446 probesets identified in the supervised analysis and generate a robust list of cartilage-selective genes (Table 1). A majority of these probesets (2245 probesets (> 92%)) were identified in 124 U133A and 74 U133B arrays using the Celsius database (see Materials and Methods), and represented expression from 34 normal tissues. A small proportion of the probesets (201/2446) are not found on the Affymetrix™ Human Genome U133A/B Arrays, so these probesets were identified in the analysis of 26 U133 Plus 2.0 arrays, representing eight non-cartilage tissues. A summary of the validation and the tissue distribution are available as additional files.

**Table 1:** Cartilage-selective genes validated *in silico *on U133A (left), U133B (center), and U133 Plus 2.0 (right) platforms.

**U133A SYMBOL**	**Probe**	**CV**	**Class**	**Mouse**	**Human**	**SAM**	**Fold**		**U133B SYMBOL**	**Probe**	**CV**	**Class**	**Mouse**	**Human**	**SAM**	**Fold**		**U133 2.0 SYMBOL**	**Probe**	**CV**	**Class**	**SAM**	**Fold**
				
AGC1	207692_s_at	9.0	ST		x	14	65.6		COL11A1	229271_x_at	11.0	ST	x	x	3	54.2		FLJ16008	1568868_at	6.4		44	8.2
AGC1	217161_x_at	9.2	ST		x	31	15.5		230895_at	230895_at	11.3				4	67.0		UBE3C	1560739_a_at	8.5	EZ	305	5.1
MATN1	206905_s_at	9.6	ST			22	75.3		EDIL3	233668_at	11.8	SI			221	6.0		1563414_at	1563414_at	8.8		77	5.1
COL10A1	217428_s_at	9.9	ST	x	x	284	5.9		C10orf49	236800_at	15.5				13	76.0		IRAK2	1553740_a_at	11.0	SI	107	6.3
MATN3	206091_at	10.1	ST		x	32	6.7		IRAK2	231779_at	15.6	SI			158	25.1		COL25A1	1555253_at	11.4	ST	516	5.1
MMP13	205959_at	10.2	ME	x	x	6	28.9		SLC4A5	234976_x_at	16.3	EZ			1036	6.4		KIAA0701	1554292_a_at	11.6		57	5.9
MATN4	207123_s_at	10.4	ST			59	7.8		LOC399959	239672_at	16.4				633	10.2		SULT1C2	1553321_a_at	11.7	ME	105	7.5
AGC1	205679_x_at	10.6	ST		x	18	28.6		IBSP	236028_at	16.4	SI			628	19.0		ITGB1	1561042_at	11.7	SI	542	7.0
COL11A2	216993_s_at	11.5	ST	x	x	116	7.3		EDNRA	243555_at	22.0	SI	x		213	5.6		RP4-756G23.1	1557123_a_at	13.1		214	6.2
NOS2A	210037_s_at	11.6	SI			20	18.7		HSUP1	229899_s_at	22.2				1994	5.1		NRP2	1555468_at	13.7	SI	45	10.2
LECT1	206309_at	13.3	SI	x		34	52.3		COL9A2	232542_at	22.5	ST		x	1929	5.0		MSI2	1552364_s_at	14.2	SI	178	5.2
HAS2	206432_at	14.2	ME			91	5.4		CMAH	229604_at	23.0	EZ			272	8.0		PTGFR	1555097_a_at	17.0	SI	470	8.0
WISP1	206796_at	15.6	SI			15	7.1		SNX5	223666_at	23.1	SI			108	5.3		BCL10	1557257_at	17.5	EZ	1014	6.1
HAPLN1	205523_at	18.5	ST	x		8	18.8		EDG2	232716_at	23.2	SI	x		712	5.4		WTAP	1560274_at	17.5	SI	228	6.3
COL9A1	222008_at	19.4	ST	x	x	19	185.1		TNFRSF18	224553_s_at	23.9	SI			124	9.4		RUNX1	1557527_at	17.8	SI	297	13.0
C1QTNF3	220988_s_at	20.3				291	5.4		USP12	236975_at	23.9	EZ			1776	6.3		LACTB	1552485_at	18.6	EZ	659	6.5
COL11A2	213870_at	20.5	ST	x	x	11	136.9		RP6-213H19.1	224407_s_at	25.3	SI			313	5.8		WWP2	1552737_s_at	21.1	EZ	144	10.9
COL10A1	205941_s_at	20.5	ST	x	x	684	38.5		BIC	229437_at	26.9	SI			571	5.5		PITPNC1	1568949_at	21.5	SI	258	5.5
NGFB	206814_at	21.0	SI			78	7.1		KIAA1718	225142_at	27.8				651	11.5		ARIH1	1558710_at	22.2	EZ	1264	5.2
PDPN	204879_at	21.5	ST	x		103	8.8		RB1CC1	237626_at	27.9	SI			1489	5.5		ADAMTS9	1554697_at	22.7	ME	769	7.4
222348_at	222348_at	21.8				227	8.4		WTAP	244219_at	28.1	SI			371	6.1		SYNJ2	1555009_a_at	23.7	SI	277	5.9
SLC28A3	220475_at	21.9	SI			296	5.1		RHOQ	239258_at	28.2	SI			784	5.4		SRGAP1	1554473_at	23.8	SI	809	5.1
EIF2C2	213310_at	22.4	EZ			1071	9.1		RPS6KA3	241460_at	28.3	SI	x	x	529	9.1		MGC17337	1552277_a_at	24.2		690	6.0
SOX5	207336_at	23.5	SI	x		1250	8.2		SEMA6D	233801_s_at	29.1	SI			245	5.3		1555841_at	1555841_at	24.5		1606	6.8
DSPG3	206439_at	24.0	ME			5	15.0		FN1	235629_at	29.6	ST			622	10.5		CD44	1565868_at	24.8	SI	1025	6.2
COL11A1	37892_at	25.7	ST	x	x	12	82.4		ULBP2	238542_at	29.7	SI			101	6.1		SLC41A2	1562208_a_at	24.9	ST	407	8.7
COL11A1	204320_at	26.0	ST	x	x	73	63.6		229221_at	229221_at	30.1				176	7.6		RHOF	1554539_a_at	26.7	SI	704	6.4
SOD2	215078_at	26.2	EZ			76	10.7		PTK2	241453_at	30.2	SI			355	7.7		ARF1	1565651_at	27.7	SI	768	5.6
HSPC159	219998_at	27.6	EZ			126	8.1		CHST11	226368_at	30.6	ME			310	5.3		1552288_at	1552288_at	28.0		127	17.1
BMP2	205289_at	29.3	SI			591	17.4		VASN	225867_at	31.9	SI			593	5.9		B3GNT5	1554835_a_at	29.5	ME	331	5.5
CSPG4	214297_at	29.5	ST			17	39.5		SCUBE3	230290_at	33.3	SI			123	6.4		B3GNT7	1555963_x_at	30.4	ME	339	18.3
CHST3	32094_at	30.0	EZ		x	1560	6.3		LOC338758	238893_at	33.4				998	5.2		SETD5	1569106_s_at	31.2		839	6.0
RELB	205205_at	30.1	SI			390	7.7		LOXL4	227145_at	33.8	EZ			260	7.5		OSMR	1554008_at	31.9	SI	85	50.3
CYTL1	219837_s_at	30.7	SI			151	17.0		SLC25A37	242335_at	33.9	SI			765	12.3		ZFYVE16	1554638_at	32.2		722	5.2
FZD10	219764_at	30.8	SI			51	7.3		228910_at	228910_at	34.0				630	5.4		AKR1C2	1562102_at	32.8	EZ	559	5.7
BDKRB1	207510_at	31.6	SI			239	5.1		PITPNC1	239808_at	34.3	SI			1129	11.8		WTAP	1558783_at	33.2	SI	946	8.0
ITGA10	206766_at	32.6	SI	x		41	29.4		FNDC3B	222693_at	34.3	SI			1394	10.2		LRRC8C	1558517_s_at	33.4		731	5.3
RNF24	210706_s_at	32.6				774	5.1		PDPN	226658_at	35.2	ST			125	15.7		1552289_a_at	1552289_a_at	34.4		164	28.5
NUPL1	204435_at	33.0	SI			1680	5.2		LOC201181	241383_at	35.2				173	14.5		SFXN3	1559993_at	35.7	SI	160	7.8
RNF24	204669_s_at	33.0				1349	6.0		FNDC3B	244022_at	37.0	SI			456	7.3		LRP11	1561180_at	37.8	SI	393	10.3
AKR1C2	217626_at	33.6	EZ			595	12.3		CHST11	226372_at	37.9	ME			554	5.9		B3GNT7	1555962_at	38.2	ME	396	12.5
LOC283824	213725_x_at	35.5				1433	6.5		225611_at	225611_at	38.2				1117	7.4		ZNF146	1569312_at	38.3		374	10.5
PDLIM4	214175_x_at	35.6	SI			212	5.6		NRP2	232701_at	38.6	SI			58	6.5		ZCCHC7	1556543_at	41.5		1906	6.2
FOSL1	204420_at	37.8	SI		x	458	15.2		TNFRSF10D	227345_at	38.7	SI			363	6.5		ATF1	1565269_s_at	42.0	SI	533	6.7
HAPLN1	205524_s_at	38.4	ST	x		16	137.6		FNDC3B	232472_at	39.4	SI			327	5.9		HIG2	1554452_a_at	43.0	EZ	119	44.6
MIA	206560_s_at	41.0	ST	x		36	8.1		229242_at	229242_at	39.7				330	6.3		KLF7	1555420_a_at	43.3	SI	1056	5.7
MMP12	204580_at	41.3	ME			29	8.2		ANKRD28	229307_at	39.7	SI			820	7.6		BCL2L11	1558143_a_at	43.8	EZ	683	6.2
TNMD	220065_at	41.9	ST	x		113	5.0		TRPS1	234351_x_at	39.7	SI		x	841	5.6		TGIF	1566901_at	43.8	SI	380	6.1
RLF	204243_at	43.0	SI			478	7.8		GLIS3	230258_at	40.2	SI			872	5.4		MCOLN2	1555465_at	43.9		520	8.6
EDIL3	207379_at	44.2	SI			84	5.4		SCUBE3	228407_at	40.2	SI			515	5.9		ChGn	1569387_at	44.7	ME	843	5.4
THBS3	209561_at	45.0	SI	x		716	7.2		SCYL1BP1	226337_at	40.5				1714	6.9		PTK2	1559529_at	45.1	SI	562	6.2
MMP3	205828_at	46.6	EZ	x		1	73.5		YME1L1	232216_at	40.9	ME			2251	5.5		FAM62B	1555830_s_at	49.5		837	6.6
RELA	209878_s_at	47.1	SI			971	7.3		KIAA0999	242920_at	41.0				1731	8.4							
LIF	205266_at	47.9	SI			120	15.7		236289_at	236289_at	41.2				445	5.4							
BMP6	206176_at	48.3	SI	x		60	38.9		GALNTL2	236361_at	42.1	ME			290	15.7							
ETNK1	219017_at	49.0	EZ			1400	5.1		PET112L	228441_s_at	42.2	EZ			333	9.8							
									ZNF697	227080_at	42.2				1119	5.5							
									FNDC3B	222692_s_at	42.3	SI			747	9.4							
									GPC6	223730_at	42.4	SI			511	9.5							
									COL27A1	225292_at	42.7	ST			135	5.4							
									NRP2	229225_at	43.1	SI			96	9.8							
									UFM1	242669_at	44.1	EZ			624	6.6							
									ASAM	228082_at	44.3	SI			68	11.7							
									KCNT2	234103_at	44.4	EZ			299	5.3							
									ERO1L	222646_s_at	44.6	EZ			1358	6.8							
									MAST4	225613_at	44.6	SI			642	7.9							
									228314_at	228314_at	44.7				1501	6.5							
									C8orf72	232668_at	45.0				400	15.6							
									RPS6	238156_at	45.7	EZ			1922	5.1							
									SQSTM1	244804_at	46.6	EZ	x		1440	5.6							
									230204_at	230204_at	46.8				26	78.1							
									TBX15	230438_at	46.9	SI			932	5.9							
									244533_at	244533_at	47.0				752	5.5							
									235821_at	235821_at	47.2				35	9.9							
									ZNF160	239954_at	47.5				1897	7.1							
									SERPINE2	236599_at	47.9	EZ			80	12.1							
									236685_at	236685_at	49.5				1555	5.5							

Genes were ranked by selectivity (CV) and classified into four functional classes Structural Protein (ST), Enzyme (EZ), Signaling (SI), and Extracellular Matrix Enzyme (ME). Genes associated with skeletal phenotypes in mice and/or human or genes are denoted in Mouse and Human columns, respectively. SAM defines the rank order of each gene identified by two-class SAM analysis of cartilage versus non-cartilage tissues. The average cartilage expression divided by the median of non-cartilage expression (Fold) is listed for each gene.

Of the three platforms, the U133A dataset was the most robust with regard to the number of arrays, biological replicates, diversity of tissues, probes identified, and gene annotation. From this platform, 1363 of the 2446 probesets identified in the SAM analysis as expressed at a higher level in cartilage were obtained. Two hundred seventy-four of the 1363 probesets (274/1363), representing 237 genes, exhibited at least five-fold higher expression when compared to non-cartilage tissues and were ranked by cartilage-specificity using an analog of coefficient of variation (CV) (see Methods). Of these, 56 probesets, representing 49 genes, were identified with a CV < 50% in non-cartilage samples, constituting the cartilage-selective gene set from this platform (Table 1, left). Twenty of these genes have mutations that have been associated with skeletal phenotypes in humans and/or mice, representing 44% of the probes selected from this platform.

Eight hundred eighty-two of the 2446 probesets were identified from the U133B validation set. Two hundred fourteen of these probesets, representing 158 genes, were well measured in cartilage with at least five-fold higher expression in cartilage relative to non-cartilage tissues. Of these, 77 probesets had a CV less than 50% in non-cartilage samples, representing 71 cartilage-selective genes (Table 1, center), including 3 genes also identified using the U133A platform (*COL11A1*, *EDIL3*, and *PDPN*).

A subset of the cartilage-selective genes was represented only on the Human Genome U133 Plus 2.0 arrays and were selected from the analysis of 28 non-cartilage samples. In total 201/2446 probesets were not represented in the U133A/B array set. Of these 201 probesets, 96 probesets, representing 85 genes, had a five-fold higher expression in cartilage than non-cartilage samples. By including the CV selection criterion, 52 probesets, representing 50 cartilage-selective genes were identified and added to the complete tally (Table 1, right), including 6 genes also identified using the U133A and U133B arrays (*IRAK2*, *NRP2*, *WTAP*, *PITPNC1*, *AKR1C2*, and *PTK2*).

In summary, 480 genes demonstrating enriched or specific expression in cartilage were selected from the comparison of cartilage and non-cartilage tissues with data derived from the U133A (n = 237), U133B (n = 158) and U133 Plus 2.0 (n = 85) platforms. Of these, a non-redundant set of 161 genes (Table 2), including 11 uncharacterized genes and 16 genes represented by unannotated probesets, were classified as cartilage-selective. These data greatly expand the number of genes known to be selectively expressed in cartilage and emphasize the unique pattern of gene expression that determines its properties.

**Table 2:** Non-redundant set of 161 cartilage-selective genes organized by chromosomal location.

Chromosomal Location	Symbol
chr1p11.1	TBX15
chr1p12	ZNF697
chr1p13.1	NGFB
chr1p21	COL11A1
chr1p22	BCL10
chr1p22	MCOLN2
chr1p22.2	228314_at
chr1p22.2	LRRC8C
chr1p31.1	PTGFR
chr1p32	RLF
chr1p33-p32	COL9A2
chr1p35	MATN1
chr1p36.21	PDPN
chr1p36.3	TNFRSF18
chr1q21	ITGA10
chr1q21	THBS3
chr1q24.2	SCYL1BP1
chr1q31.3	KCNT2
chr1q41	244533_at
chr1q42	ARF1
chr1q42.13	222348_at
chr2p13	SLC4A5
chr2p14	HSPC159
chr2p21	RHOQ
chr2p24-p23	MATN3
chr2q11.1-q11.2	SULT1C2
chr2q13	236289_at
chr2q13	BCL2L11
chr2q14.3	FLJ16008
chr2q32	KLF7
chr2q33.3	NRP2
chr2q33-q35	SERPINE2
chr2q34	FN1
chr2q37.1	B3GNT7
chr3p14.3-p14.2	ADAMTS9
chr3p24.3	ANKRD28
chr3p24.3	GALNTL2
chr3p25.3	IRAK2
chr3p25.3	SETD5
chr3q26.31	FNDC3B
chr3q28	B3GNT5
chr4p16-p15	CYTL1
chr4q21-q25	IBSP
chr4q22.1-q23	229221_at
chr4q25	COL25A1
chr4q27-q28	PET112L
chr4q31.23	EDNRA
chr4q35.1	1563414_at
chr5p13.1	OSMR
chr5p13.3	C1QTNF3
chr5p15.2-q14.3	ZFYVE16
chr5q12.3	225611_at
chr5q12.3	MAST4
chr5q14	EDIL3
chr5q14.3	230204_at
chr5q14.3	230895_at
chr5q14.3	HAPLN1
chr5q31.1	PDLIM4
chr5q35	SQSTM1
chr6p21.3	COL11A2
chr6p21.3	SCUBE3
chr6p21.32	CMAH
chr6q24.2	236685_at
chr6p24-p23	BMP6
chr6q12-q14	COL9A1
chr6q21-q22	COL10A1
chr6q25	ULBP2
chr6q25.1	LRP11
chr6q25.3	SOD2
chr6q25.3	SYNJ2
chr6q25-q27	WTAP
chr7q32.1	HIG2
chr7q34	KIAA1718
chr7q36.3	FAM62B
chr7q36.3	UBE3C
chr8p21	TNFRSF10D
chr8p21.2	SLC25A37
chr8p21.3	ChGn
chr8p22-q21.13	RB1CC1
chr8q12.1	C8orf72
chr8q24	EIF2C2
chr8q24.12	HAS2
chr8q24.12	TRPS1
chr8q24.1-q24.3	WISP1
chr8q24.22	235821_at
chr8q24-qter	PTK2
chr9p13.2	ZCCHC7
chr9p21	RPS6
chr9p24.2	GLIS3
chr9q22.2	SLC28A3
chr9q31.1	1555841_at
chr9q31.1	MGC17337
chr9q31.3	EDG2
chr9q32	229242_at
chr9q32	COL27A1
chr10p11.2	ITGB1
chr10p13	C10orf49
chr10p14	YME1L1
chr10p15-p14	AKR1C2
chr10q22.1	CHST3
chr10q24	LOXL4
chr10q24.31	SFXN3
chr11p11.2	228910_at
chr11p13	CD44
chr11q13	FOSL1
chr11q13	RELA
chr11q22.3	MMP12
chr11q22.3	MMP13
chr11q22.3	MMP3
chr11q23.3	KIAA0999
chr11q24.1	ASAM
chr11q24.1	LOC399959
chr12p12.1	ETNK1
chr12p12.1	SOX5
chr12q	CHST11
chr12q13	ATF1
chr12q14.2	SRGAP1
chr12q21	DSPG3
chr12q21.33	LOC338758
chr12q23.1	KIAA0701
chr12q23.3	SLC41A2
chr12q24.31	RHOF
chr12q24.33	FZD10
chr13q12.13	NUPL1
chr13q12.13	USP12
chr13q13.3	UFM1
chr13q14-q21	LECT1
chr13q32	GPC6
chr14q22.1	ERO1L
chr14q32.1-q32.2	BDKRB1
chr15q21.1	SEMA6D
chr15q22.1	LACTB
chr15q24	ARIH1
chr15q24.2	CSPG4
chr15q26.1	AGC1
chr16p13.12	LOC283824
chr16p13.3	VASN
chr16q22.1	WWP2
chr17q11.2-q12	NOS2A
chr17q21.2	LOC201181
chr17q22	MSI2
chr17q24.2	PITPNC1
chr18p11.3	TGIF
chr19p13.11	1552288_at
chr19p13.11	1552289_a_at
chr19q13.1	ZNF146
chr19q13.32	RELB
chr19q13.32-q13.33	MIA
chr19q13.41	ZNF160
chr20p11	SNX5
chr20p12	BMP2
chr20p13-p12.1	RNF24
chr20q13.13	HSUP1
chr20q13.1-q13.2	MATN4
chr21q21.3	BIC
chr21q22.3	RUNX1
chr22q12.2	LIF
chr22q13.2	RP4-756G23.1
chrXp22.2-p22.1	RPS6KA3
chrXq21.33-q23	TNMD
chrXq26.2	RP6-213H19.1

### qRT-PCR validation

Quantitative RT-PCR was used to independently assess the tissue-selectivity of the genes identified in the microarray analysis. For each of the three microarray platforms, the probesets with a CV less than 50% were divided into 10% intervals (0–10% CV, 10–20% etc.) (Table 1), and one gene from the middle of each interval was selected for analysis by qRT-PCR.

All of the thirteen of genes analyzed demonstrated higher expression in cartilage than in the seven non-cartilage tissues studied (Table 3). Also, with one exception (*OSMR*), the selection threshold of at least five-fold higher expression in cartilage tissues as compared with the average expression among all non-cartilage tissues imposed for the microarray analysis, was observed. For most of the genes studied by qRT-PCR, there was little expression in the seven non-cartilage tissues (median Ct = 33.2), indicating that including the coefficient of variation in the ranking algorithm preferentially identifies genes selectively expressed in cartilage. Also, there was an inverse correlation between the gene rank and the standard deviation in expression level among non-cartilage tissues, indicating that genes with a higher rank were more selectively expressed in cartilage. Finally, there was a trend of decreasing cartilage selectivity moving from the U133A to U133B to U133 2.0 qRT-PCR validations, likely reflecting the decreasing robustness of comparison datasets in the respective platforms. Overall the qRT-PCR experiments replicated and validated findings derived from the comparative microarray data.

**Table 3:** Summary of qRT-PCR amplification of cartilage-selective genes in fetal cartilage and seven non-cartilage tissues.

**CV**			**Ct**		
**Interval**	**Symbol**	**Neg**	**NC**	**C**	**Fold**
**U133A**					
**0–10%**	**AGC1**	2	33	23	371
**10–20%**	**NOS2A1**	3	34	23	1158
**20–30%**	**DSPG3**	6	35	25	661
**30–40%**	**BDKRB1**	2	34	28	49
**40–50%**	**MMP3**	3	33	20	4988
**U133B**					

**10–20%**	**C10orf49**	7	35	23	Unique
**20–30%**	**KIAA1718**		30	27	8
**30–40%**	**LOC20118**	4	34	29	20
**40–50%**	**ASAM**		31	26	29
**U133 2.0**					

**10–20%**	**KIAA0701**		30	27	5
**20–30%**	**MGC17337**		30	27	5
**30–40%**	**OSMR**		28	27	3
**40–50%**	**HIG2**		30	25	21

Representative genes are listed from each validation platform in order of 10% CV interval. (Neg) Number of non-cartilage tissues in which amplification was not detected. Average Ct values for each gene were calculated for both cartilage (C) and non-cartilage (NC) tissues. Where no amplification was observed the maximum Ct value (i.e. 35) was used for calculations. Fold difference (Fold) is calculated from the difference in cartilage and non-cartilage Ct values.

## Discussion

Using genome-scale microarrays, gene expression in human fetal cartilage was compared with a robust set of other normal tissues. Hierarchical clustering showed remarkable similarity among the 18–22 week fetal cartilage expression profiles and demonstrated that a subset of the cartilage transcriptome is composed of a unique gene set not generally expressed in the other tissues studied. Using SAM, 2446 probesets measured preferential expression of 1712 genes with at least three-fold higher expression in cartilage as compared with other tissues. 1028 (42%) of these probesets matched genes identified in a cartilage growth plate cDNA library [4] validating their expression in cartilage via an independent dataset. The identification of genes known to have restricted patterns of expression in cartilage confirmed the presence of RNA derived from the reserve (*GREM1*), hypertrophic (*BMP6, COL10A1*), and terminally differentiated (*MMP13*) chondrocytes, in addition to genes expressed throughout all zones of the growth plate. This analysis suggested that there is differential transcriptional regulation of many genes in fetal cartilage and that the data could be used to identify genes selectively expressed in the tissue.

Tissue-selective genes have been previously defined as genes with enriched expression in a particular tissue [14] and characterized with algorithms dependent on the degree of differential expression relative to other tissues, including *t*-test [15], SAM [16], fold change [14,17,18], and enrichment scores [14]. While these approaches successfully identify tissue-selective genes, the reliance on fold change reduces the significance of many selectively expressed genes with low fold change. To compensate for this and identify cartilage-selective genes expressed at lower levels, the approach presented here placed increased significance on the preferentially expressed genes that showed the least variation of expression in non-cartilage tissues. This was made possible by the use of publicly-released reference gene expression data performed on the same platform and led to the reliable identification of genes with lower fold changes, but high cartilage selectivity. The impact of the use of coefficient of variation on the ranked gene list is apparent in Tables 1 and 2. In the U133A dataset, nine of the top 25 genes were ranked higher than 100 in significance in the SAM ranking. The average fold change of the probes for these nine genes was 10.7, while the average fold change of the probes for the other 14 of the top 25 genes was 42.2. One of these probes, *COL10A1 *was among the top four cartilage-selective genes using the CV algorithm but ranked at 284 by SAM (Table 1). In the U133B dataset, which contains a higher percentage of unannotated genes, 4 of the top 50 probes had a SAM ranking below 100, and the average SAM ranking was 576. Overall, to identify only the most cartilage-selective genes, a threshold of 50% coefficient of variation was used across all three platforms, yielding 161 cartilage-selective genes. A subset of 13 of the 161 cartilage-selective genes was studied by quantitative RT-PCR in cartilage and eight non-cartilage tissues to independently assess tissue selectivity. The data confirmed the cartilage-selectivity of genes with less than 50% CV, validating the selection procedure and suggesting that the gene expression patterns determined by microarray analysis are representative.

The coefficient of variation selection approach could, in theory, equally select for three different patterns of expression: cartilage-specific genes; genes with a consistent level of baseline expression in non-cartilage tissues; and genes with significant but equal expression in all tissues (e.g. housekeeping genes). In this data analysis, however, the most highly ranked genes consistently demonstrated little or no expression in non-cartilage tissues. The data thus demonstrate that incorporating coefficient of variation preferentially selected for genes not significantly expressed in non-cartilage tissues, yielding genes likely to have important and perhaps unique roles in cartilage.

Regardless of expression level, a cartilage-selective expression pattern suggests that the product of each identified gene may have a functional role in the development of the skeleton. Concordant with this hypothesis, mutations in 25 of the 161 selected genes have been associated with skeletal phenotypes in humans and/or mice. Included among them were the products of the well characterized genes encoding aggrecan and the cartilage-specific collagens, gene products known to have a prominent role in skeletal development and endochondral ossification. By this measure, the remaining genes may be candidate genes for skeletal dysplasias in which the disease gene has yet to be identified. As new skeletal dysplasia loci are defined, coincidence between a locus and a cartilage-selective gene may promote rapid identification of the disease gene. Knockout of the orthologous genes in mice would also facilitate exploring the role of each gene in skeletal development.

Classification of the biological roles of the products of the cartilage-selective gene set reveals genes with diverse functions including structural proteins of the cartilage extracellular matrix, enzymes that modify them, and 41 gene products with unannotated function. There were 65 genes that are components of signaling pathways, and only 43% of these were identified by sequence analysis of a comparable fetal cartilage cDNA library [4]. Among the genes were elements of the nitric oxide, VEGF, TNF/RANK, and gp130 pathways, all of which have known roles in the growth plate [19-23]. Mutations in the genes encoding some of the molecules in these pathways, including *RPS6SKA3*, *LIFR*, *TNFRSF11A *and *IKBKG*, have been associated with human skeletal dysplasias [12], again suggesting that the remaining genes may also serve critical roles in endochondral ossification.

Multiple genes encoding members of the LIF/gp130 signaling pathway met the definition of cartilage-selective genes. LIF is a cytokine that is expressed in terminally-differentiated growth plate chondrocytes [24] and signals through the gp130/LIFR complex. Homozygosity for loss of function mutations in the LIF receptor produces the recessively inherited skeletal dysplasia, Stuve-Wiedemann syndrome [25]. In addition to their skeletal features, these patients have cardiovascular, pulmonary, gastrointestinal, neurologic and metabolic abnormalities, likely attributable to the role that LIFR plays in embryonic or fetal development. Genes on the cartilage-selective gene list upstream of the receptor include *RELA *and *RELB*, NF-KB survival transcription factors that increase transcription of LIF [26], as well as the *LIF *gene itself. Through the LIFR/gp130 complex, LIF can regulate both the JAK/STAT and ERK MAP kinase pathways. Pathway components downstream of the receptor include ATF1, part of the ATF1/CREB transcription factor complex that participates in ERK MAP kinase signaling [27,28]. The ATF1/CREB complex is also regulated by phosphorylation by the product of the *RPS6SKA3 *gene [29,30], another gene in the MAPK/ERK pathway that is associated with a skeletal phenotype. The gene encoding RPS, a phosphorylation target of RPS6SKA3 [30], was also cartilage-selective, but the role of this protein in growth plate differentiation has yet to be determined. Finally, the gene encoding FOSL1, a FOS-like transcription factor activated by the ERK/MAPK pathway which binds cJUN to form a transcription complex [31,32], was among the cartilage-selective genes identified. Thus comparative microarray analysis has identified multiple components of a regulatory pathway that can be explored to further evaluate their importance in growth plate differentiation and endochondral ossification.

While this study has provided a deep set of genes that exhibit a cartilage-selective expression pattern, there are some limitations to the analysis. First, the study focused on total cartilage RNA, including all types of growth plate chondrocytes, as well as articular cartilage. As a result, it cannot be determined if the selected genes are expressed in all types of chondrocytes or only a subset of cells. In this context, nine of the cartilage selective genes have been shown to be more highly expressed in hypertrophic cells relative to proliferating chondrocytes in the rat and/or mouse [7,8]. Second, the cartilage samples were derived from a single anatomic site and a narrow window of fetal development, so it is unclear to what extent the observed gene expression pattern can be generalized. Third, neither all possible non-cartilage tissues nor each type of cell within each tissue were studied, so cartilage-selectivity could be affected if additional fetal and/or adult tissues that express the identified genes were found. This may be particularly important for other connective tissues such as bone, tendon and ligament which contain cells known to express some of the cartilage-selective genes identified here.

Not all genes selectively expressed in developing cartilage will necessarily be identified using this approach. For instance, the *COL2A1 *gene fell just below the rigorous 50% CV standard set to define cartilage-selectivity. The underlying reasons for this are complex. Probe performance as well as the known expression of *COL2A1 *in fetal liver and heart, are likely to have had an effect, as both factors could have contributed to the variation in expression in non-cartilage tissues. In addition, the approach presented here treated the three expression platforms, U133A, U133B and U133 2.0 equally from the viewpoint of the threshold for cartilage-selectivity. Because the comparative dataset of normal tissues was both broader and deeper for the 133A platform, additional genes from this platform, albeit with greater than 50% CV, could be considered to be tissue-selective (e.g. *COL2A1*). Thus, a platform independent threshold would likely yield additional genes of interest within the U133A dataset. Finally, tissue-specific genes were identified using only microarrays and a single generalized algorithm. Additional genes selectively expressed in cartilage could be identified by less stringent criteria or other methods.

## Conclusion

Genome-scale comparative expression analysis using human fetal cartilage and a broad set of normal human tissues has identified 161 cartilage-selective genes, including 27 uncharacterized genes. The data identify novel gene products that may provide essential roles in normal skeletogenesis and suggest new candidates for the over 100 inherited skeletal disorders in which the disease gene has not been identified. The results demonstrate that fetal cartilage is a complex and transcriptionally active tissue, and that the set of genes selectively expressed in cartilage has been greatly underestimated.

## Methods

A flow chart outlining methods and results as well as other supplemental information is provided in additional data file 1.

### Cartilage specimen collection and processing

**Figure 1:**
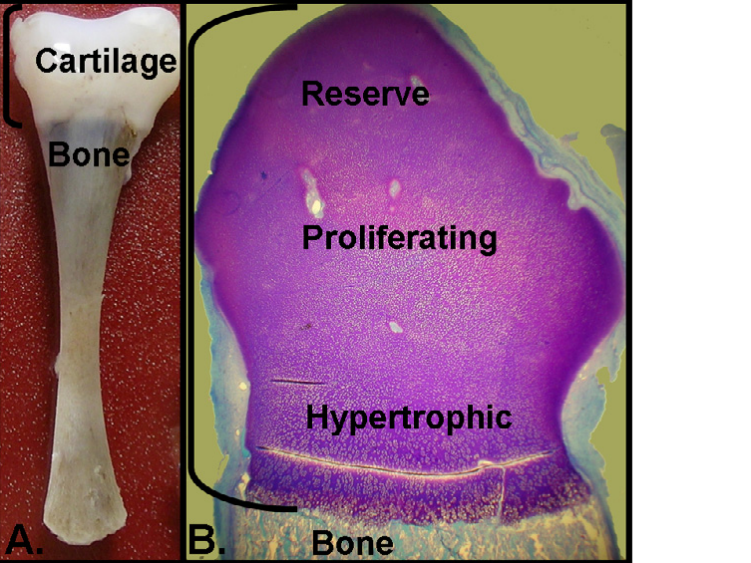
**Human fetal cartilage section dissected for RNA expression profiling**. (A) Normal distal femur cartilage from an 18–22 week fetus. Brackets define the cartilage that was dissected and used for RNA profiling. (B) Toluidine blue stained longitudinal section of the distal head of the femur magnified 8.5 fold. The dissected portion included chondrocytes from the articular, reserve, proliferating, and hypertrophic zones.

Seven independent 18–22 week normal human fetal cartilage samples were studied under an Institutional Review Board approved protocol. Cartilage from the distal femur was dissected to remove bone and any adherent non-cartilage tissue (Figure 1). RNA was isolated and purified as previously described [4] and the quality and quantity of RNA were confirmed using an Agilent 2100 bioanalyzer and a Nanodrop ND-1000 spectrophotometer, respectively. Probe labeling, microarray hybridization, washing and scanning were carried out as detailed in Affymetrix protocols [33]. Five samples were used to probe Affymetrix™ U133 Plus 2.0 microarrays; and two samples were used to probe the Affymetrix™ Human Genome U133A/B set. Annotations were from version 11/15/06. The data are publicly available in the GEO database series [GEO:GSE6565]. An additional sample was fixed in formalin, sectioned and stained with toluidine blue.

### Non-cartilage microarray data

This project made use of the Celsius database [34,35], which is a database of publicly available microarray datasets from Gene Expression Omnibus, Array Express, and individual databases. Only CEL files are entered into the database, permitting reprocessing using identical algorithms to enable experimental comparisons. Only data from Affymetrix™ Human Genome U133A/B and Plus 2.0 platforms that contained clear annotation that they were derived from normal human tissues were selected for this analysis.

### Microarray analysis

#### Data normalization and transformation

Raw data were normalized using the RMA algorithm with default parameters, available as part of the Bioconductor R library [36,37]. In brief, each CEL file was processed separately with an invariant pool of 50 arrays from a matching platform. Higher signal intensities observed in a subset of U133A non-cartilage samples from one provider [38] were additionally normalized by subtracting the median from other non-cartilage samples. After normalization, the training dataset was log_2 _transformed prior to analysis.

### Median derived analog of CV for cartilage-selectivity ranking

In highly expressed cartilage genes, the degree of cartilage selectivity was defined as a median derived analog of CV (average deviation/median) applied to expression of these genes in non-cartilage tissues. The analog of CV was used to allow for greater tolerance of gene expression in some cartilage containing tissues without affecting the cartilage-selective assessment. A CV of 50% was empirically determined as a mathematically acceptable threshold for cartilage selectivity for probes across all three validation datasets (U133A, U133B, and U133 Plus 2.0).

### Training

For the unsupervised analysis, probe intensities in all tissues were subtracted by the median probe intensity in cartilage, so all expression was defined relative to cartilage (i.e. the median of cartilage expression was set at zero) for each selected probe. Probesets with the greatest variation across all tissues and whose expression in at least two samples differed by two standard deviations from their mean expression across the entire set of samples were selected. Two-way hierarchical clustering was performed using Pearson's correlation to group genes and arrays based on the similarity of their expression patterns [39]. For the supervised analysis, the significance analysis of microarrays (SAM) two class method [40] was applied. 100 hundred permutations were used and at least three-fold variation between cartilage and non-cartilage expression was required.

### Validation

The probeset expression profiles for the 2,446 probesets identified in the training set (see Results) were acquired from 224 arrays representing 34 different tissues on three different platforms: Affymetrix U133A, U133B and U133 Plus 2.0. The data from the first platform, U133A, consisted of 1363 probeset profiles from 124 arrays, representing two normal fetal cartilage and 122 normal non-cartilage (32 tissues) samples (see additional data file 3, for the tissue distribution of samples used in this analysis). Arrays represented in this dataset were mostly from two large normal tissue expression profiling projects [38]. The second dataset, U133B, consisted of 882 expression profiles identified on 72 U133B microarrays from two normal fetal cartilage and 74 normal non-cartilage samples. These samples were primarily from the UCLA normal tissue microarray project (Chen, Day and Nelson, unpublished). The U133 2.0 dataset consisted of 201 expression profiles obtained from 26 Human Genome U133 Plus 2.0 arrays representing expression from five normal fetal cartilage and 21 non-cartilage (eight different tissue types) samples. The five cartilage arrays for the U133 2.0 platform were technical replicates of the arrays used in the training dataset. The non-cartilage arrays were a subset of the training dataset set aside for this validation only. From the 2446 probesets selected, probesets that exhibited at least a five-fold difference between the average cartilage intensity and the median signal intensity of all non-cartilage tissues, were selected. Cartilage-specificity was then determined using a median-derived analog of coefficient of variation (CV) as described above. Probesets with less than 50% CV were defined as reflecting cartilage-selective expression.

### qRT-PCR

One microgram of RNA from seven tissues (brain, prostate, kidney, liver, heart, thyroid, and testis) in the FirstChoice^® ^Human Total RNA survey panel (Ambion) was reverse transcribed using a high-capacity cDNA archive kit (ABI) and random primers. For cartilage, RNA from three independent cartilage samples was pooled and reverse transcribed. Amplification reactions were performed in triplicate using 100 ng of each cDNA. Thirty-five cycles of amplification were carried out in an ABI 7300 using the validated QuantiTect Gene Expression Assays and SYBR Green PCR kit (Qiagen). To assess specificity, amplification products were subjected to melting curve analysis and gel electrophoresis. The 2- [delta] [delta]Ct method was employed to calculate relative amplification. This was performed using an average of endogenous references (*18S*, *GAPDH*, and *HPRT1*) to improve normalization across the panel of tissues used [41]. For genes where no amplification was detected in a tissue, a Ct value of 35 was assigned, reflecting the maximum number of cycles carried out.

## Abbreviations

CV: coefficient of variation; SAM: significance analysis of microarrays

## Authors' contributions

VF designed and carried out the bioinformatics analysis and qRT-PCR, and drafted the manuscript. AD participated in data acquisition and normalization. DK obtained, dissected and isolated the cartilage RNA and critically revised the manuscript, ZAC participated in the bioinformatics analysis. ZC participated in generation of microarray data. SN participated in the design and coordination of the study and critically revised the manuscript. DC conceived the study, participated in its design and coordination, performed microarray analysis and critically revised the manuscript. All authors read and approved the final manuscript.

## Supplementary Material

Additional File 1 A flow chart illustrating a summary of the analysis. An unsupervised (black arrows) and supervised analysis (blue arrows) were performed with gene expression from 46 U133 2.0 Affymetrix arrays. An independent validation set comprised of 224 Affymetrix arrays (dashed arrows) was also used to test the 1713 genes for the most robust fetal cartilage selective genes. Click here for file

Additional File 2 Supervised analysis and summary of *in silico *validation. Ranked list of cartilage genes more highly expressed by at least three fold in cartilage than non-cartilage tissues in the training dataset (five fetal cartilage samples compared to 41 normal non-cartilage samples). Ranked order is based on expression profiles obtained from 46 U133 Plus 2.0 arrays analyzed with SAM 2 class analysis with 100 permutations and with a False Discovery Rate (FDR) of 0. (B) Each probeset was independently evaluated in the validation datasets five fold higher expression using independent samples and three independent platforms as outlined in methods. Present indicates probe is identified in validation platform; Enriched indicates gene is expressed five fold higher in cartilage than non-cartilage tissues; Cartilage selectivity indicates at least five fold higher expression in cartilage than non-cartilage with a CV score of < 50% in non-cartilage samples. (C) "X" denotes gene was identified in a fetal cartilage cDNA library. Click here for file

Additional File 3 Tissue distribution training and validation sets. (A) 31 Non-cartilage tissues and 124 arrays were used for the validation of cartilage selective genes identified on the U133A chip. Two fetal cartilage samples were compared against 122 non-cartilage samples. (B) 27 non-cartilage Tissues and 74 arrays were used for the validation of cartilage selective genes identified on the U133B chip. Two fetal cartilage samples were compared against 72 non-cartilage arrays (C) Eight non-cartilage tissues and 26 arrays used for the validation of cartilage selective genes identified on the U133B chip. Five fetal cartilage samples were compared against 72 non-cartilage arrays. Click here for file
