# Supplementary figures and images for: Cartilage-selective genes identified in genome-scale analysis of non-cartilage and cartilage gene expression
Source: BMC Genomics. 2007 Jun 12;8:165. doi: 10.1186/1471-2164-8-165 (PMC1906768; doi:10.1186/1471-2164-8-165)

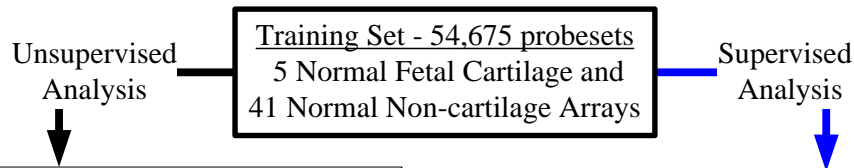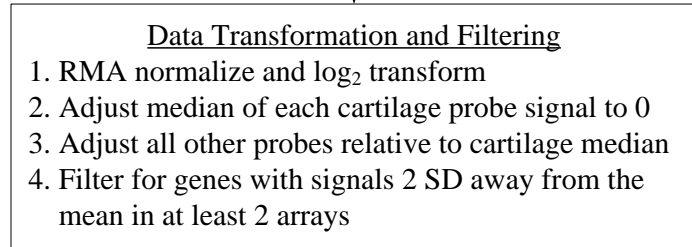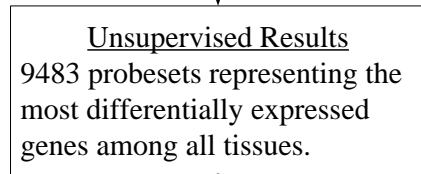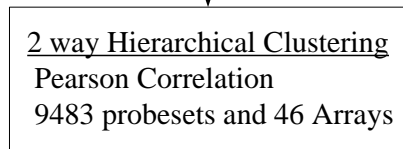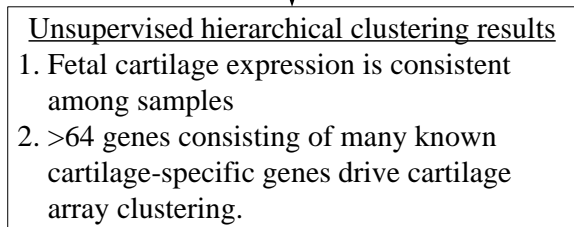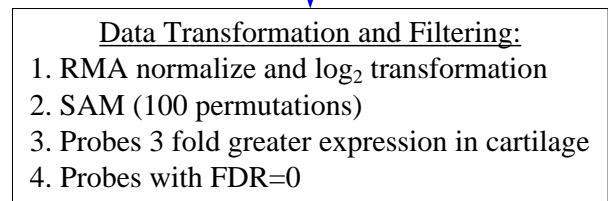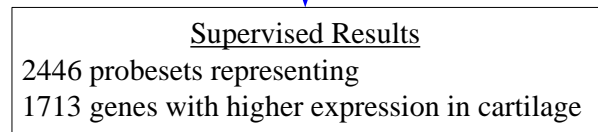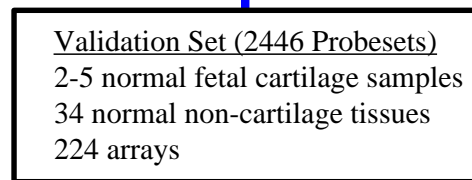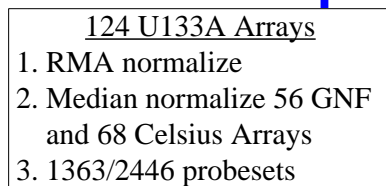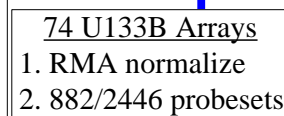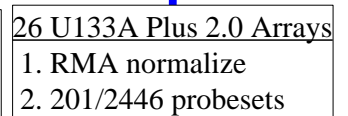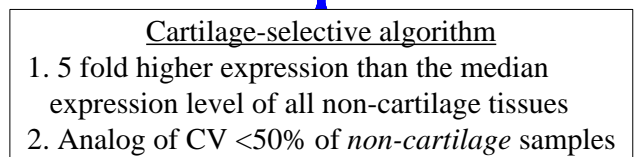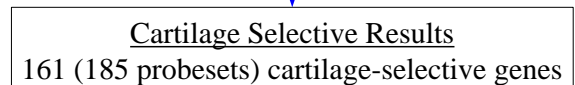

Supplement: Additional File 1 — A flow chart illustrating a summary of the analysis. An unsupervised (black arrows) and supervised analysis (blue arrows) were performed with gene expression from 46 U133 2.0 Affymetrix arrays. An independent validation set comprised of 224 Affymetrix arrays (dashed arrows) was also used to test the 1713 genes for the most robust fetal cartilage selective genes. [file 1471-2164-8-165-S1.pdf]
