# Supplementary material for: Cartilage-selective genes identified in genome-scale analysis of non-cartilage and cartilage gene expression
Source: BMC Genomics. 2007 Jun 12;8:165. doi: 10.1186/1471-2164-8-165 (PMC1906768; doi:10.1186/1471-2164-8-165)
Supplement: Additional File 2 — Supervised analysis and summary of in silico validation. Ranked list of cartilage genes more highly expressed by at least three fold in cartilage than non-cartilage tissues in the training dataset (five fetal cartilage samples compared to 41 normal non-cartilage samples). Ranked order is based on expression profiles obtained from 46 U133 Plus 2.0 arrays analyzed with SAM 2 class analysis with 100 permutations and with a False Discovery Rate (FDR) of 0. (B) Each probeset was independently evaluated in the validation datasets five fold higher expression using independent samples and three independent platforms as outlined in methods. Present indicates probe is identified in validation platform; Enriched indicates gene is expressed five fold higher in cartilage than non-cartilage tissues; Cartilage selectivity indicates at least five fold higher expression in cartilage than non-cartilage with a CV score of < 50% in non-cartilage samples. (C) "X" denotes gene was identified in a fetal cartilage cDNA library. [file 1471-2164-8-165-S2.pdf]

Supplemental Table 1.**Supervised Analysis**. (A) Ranked list of cartilage genes more highly expressed by at least three fold in cartilage than non-cartilage tissues in the training dataset (five fetal cartilage samples compared to 41 normal non-cartilage samples). Ranked order is based on expression profiles obtained from 46 U133 Plus 2.0 arrays analyzed with SAM 2 Class analysis with 100 permutations and with a False Discovery Rate of 0. (B) Each probeset was independently evaluated in the validation datasets five fold higher expression using independent samples and three independent platforms as outlined in methods. Present indicates probe is identified in validation platform; Enriched indicates gene is expressed five fold higher in cartilage than non-cartilage tissues; Cartilage selectivity indicates at least five fold higher expression in cartilage than non-cartilage with a CV score of <50% in non-cartilage samples. C. Gene was identified in a fetal cartilage cDNA library.

	**A. Training Dataset**				**B. Validation Dataset**									**C. cDNA library**
					**U133A**			**U133B**			**U1332.0**			
	Gene ID	Gene Name	Chromosome	q-value(%)	Present	Enriched	Selective	Present	Enriched	Selective	Present	Enriched	Selective	EST
1	205828_at	MMP3	chr11q22.3	0	x	x	x							x
2	204475_at	MMP1	chr11q22.3	0	x	x								x
3	229271_x_at	COL11A1	chr1p21	0				x	x	x				x
4	230895_at	---	---	0				x	x	x				x
5	206439_at	DSPG3	chr12q21	0	x	x	x							x
6	205959_at	MMP13	chr11q22.3	0	x	x	x							x
7	217404_s_at	COL2A1	chr12q13.11-q13.2	0	x	x								x
8	205523_at	HAPLN1	chr5q14.3	0	x	x	x							x
9	213492_at	COL2A1	chr12q13.11-q13.2	0	x	x								x
10	205680_at	MMP10	chr11q22.3	0	x	x								
11	213870_at	COL11A2	chr6p21.3	0	x	x	x							x
12	37892_at	COL11A1	chr1p21	0	x	x	x							x
13	236800_at	C10orf49	chr10p13	0				x	x	x				x
14	207692_s_at	AGC1	chr15q26.1	0	x	x	x							x
15	206796_at	WISP1	chr8q24.1-q24.3	0	x	x	x							
16	205524_s_at	HAPLN1	chr5q14.3	0	x	x	x							x
17	214297_at	CSPG4	chr15q24.2	0	x	x	x							
18	205679_x_at	AGC1	chr15q26.1	0	x	x	x							x
19	222008_at	COL9A1	chr6q12-q14	0	x	x	x							x
20	210037_s_at	NOS2A	chr17q11.2-q12	0	x	x	x							
21	232277_at	---	---	0				x	x					x
22	206905_s_at	MATN1	chr1p35	0	x	x	x							x
23	205713_s_at	COMP	chr19p13.1	0	x	x								x
24	232805_at	COL11A1	chr1p21	0				x						x
25	214632_at	NRP2	chr2q33.3	0	x									
26	230204_at	---	---	0				x	x	x				x
27	204298_s_at	LOX	chr5q23.2	0	x									x
28	213622_at	COL9A2	chr1p33-p32	0	x	x								x
29	204580_at	MMP12	chr11q22.3	0	x	x	x							x
30	204915_s_at	SOX11	chr2p25	0	x									
31	217161_x_at	AGC1	chr15q26.1	0	x	x	x							x
32	206091_at	MATN3	chr2p24-p23	0	x	x	x							x
33	215717_s_at	FBN2	chr5q23-q31	0	x									
34	206309_at	LECT1	chr13q14-q21	0	x	x	x							x
35	235821_at	---	---	0				x	x	x				x
36	206560_s_at	MIA	chr19q13.32-q13.33	0	x	x	x							
37	204724_s_at	COL9A3	chr20q13.3	0	x	x								x
38	227963_at	C17orf45	chr17p11.2	0				x						
39	218691_s_at	PDLIM4	chr5q31.1	0	x									x
40	220322_at	IL1F9	chr2q12-q21	0	x									

41	206766_at	ITGA10	chr1q21	0	x	x	x							x
42	205499_at	SRPX2	chrXq21.33-q23	0	x	x								x
43	206924_at	IL11	chr19q13.3-q13.4	0	x									
44	1568868_at	FLJ16008	chr2q14.3	0							x	x	x	
45	1555468_at	NRP2	chr2q33.3	0							x	x	x	
46	204736_s_at	CSPG4	chr15q24.2	0	x									
47	209270_at	LAMB3	chr1q32	0	x	x								
48	204913_s_at	SOX11	chr2p25	0	x									
49	215617_at	---	---	0	x									x
50	218468_s_at	GREM1	chr15q13-q15	0	x	x								x
51	219764_at	FZD10	chr12q24.33	0	x	x	x							
52	206805_at	SEMA3A	chr7p12.1	0	x									
53	213060_s_at	CHI3L2	chr1p13.3	0	x	x								x
54	219693_at	AGPAT4	chr6q26	0	x	x								x
55	211844_s_at	NRP2	chr2q33.3	0	x									
56	204926_at	INHBA	chr7p15-p13	0	x									x
57	1554292_a_at	KIAA0701	chr12q23.1	0							x	x	x	x
58	232701_at	NRP2	chr2q33.3	0				x	x	x				
59	207123_s_at	MATN4	chr20q13.1-q13.2	0	x	x	x							x
60	206176_at	BMP6	chr6p24-p23	0	x	x	x							x
61	216866_s_at	COL14A1	chr8q23	0	x									x
62	209589_s_at	EPHB2	chr1p36.1-p35	0	x									
63	61734_at	RCN3	chr19q13.33	0	x									
64	219454_at	EGFL6	chrXp22	0	x	x								
65	210286_s_at	SLC4A7	chr3p22	0	x									
66	215271_at	TNN	chr1q23-q24	0	x									
67	220518_at	ABI3BP	chr3q12	0	x									
68	228082_at	ASAM	chr11q24.1	0				x	x	x				x
69	216865_at	COL14A1	chr8q23	0	x									x
70	214702_at	FN1	chr2q34	0	x									x
71	241031_at	NLF1	chr15q22.2	0				x						
72	202311_s_at	COL1A1	chr17q21.33	0	x	x								x
73	204320_at	COL11A1	chr1p21	0	x	x	x							x
74	229802_at	---	---	0				x	x					x
75	227487_s_at	SERPINE2	chr2q33-q35	0				x						x
76	215078_at	SOD2	chr6q25.3	0	x	x	x							x
77	1563414_at	---	---	0							x	x	x	x
78	206814_at	NGFB	chr1p13.1	0	x	x	x							
79	235287_at	CDK6	chr7q21-q22	0				x						
80	236599_at	SERPINE2	chr2q33-q35	0				x	x	x				x
81	225566_at	NRP2	chr2q33.3	0				x	x					
82	205476_at	CCL20	chr2q33-q37	0	x	x								
83	223541_at	HAS3	chr16q22.1	0				x	x					
84	207379_at	EDIL3	chr5q14	0	x	x	x							x
85	1554008_at	OSMR	chr5p13.1	0							x	x	x	x
86	204421_s_at	FGF2	chr4q26-q27	0	x	x								x
87	227860_at	CPXM1	chr20p13-p12.3	0				x	x					
88	232453_at	GPC6	chr13q32	0				x	x					x
89	1558643_s_at	EDIL3	chr5q14	0							x	x		x
90	212938_at	COL6A1	chr21q22.3	0	x									x

91	206432_at	HAS2	chr8q24.12	0	x	x	x							x
92	215485_s_at	ICAM1	chr19p13.3-p13.2	0	x									x
93	1561886_a_at	SLC39A14	chr8p21.3	0							x	x		x
94	202638_s_at	ICAM1	chr19p13.3-p13.2	0	x	x								x
95	202998_s_at	LOXL2	chr8p21.3-p21.2	0	x	x								x
96	229225_at	NRP2	chr2q33.3	0				x	x	x				
97	232355_at	---	---	0				x						x
98	235235_s_at	FLJ36874	chr11q12.1	0				x						x
99	209955_s_at	FAP	chr2q23	0	x	x								
100	224508_at	MGC12916	chr17p11.2	0				x						
101	238542_at	ULBP2	chr6q25	0				x	x	x				
102	205290_s_at	BMP2	chr20p12	0	x	x								x
103	204879_at	PDPN	chr1p36.21	0	x	x	x							
104	239568_at	PLEKHH2	chr2p21	0				x						
105	1553321_a_at	SULT1C2	chr2q11.1-q11.2	0							x	x	x	
106	207433_at	IL10	chr1q31-q32	0	x									
107	1553740_a_at	IRAK2	chr3p25.3	0							x	x	x	x
108	223666_at	SNX5	chr20p11	0				x	x	x				
109	219367_s_at	NRP2	chr2q33.3	0	x	x								
110	212974_at	DENND3	chr8q24.3	0	x	x								
111	220898_at	---	---	0	x									x
112	239903_at	---	---	0				x						x
113	220065_at	TNMD	chrXq21.33-q23	0	x	x	x							x
114	215446_s_at	LOX	chr5q23.2	0	x									x
115	203698_s_at	FRZB	chr2qter	0	x	x								x
116	216993_s_at	COL11A2	chr6p21.3	0	x	x	x							x
117	207639_at	FZD9	chr7q11.23	0	x									
118	210261_at	KCNK2	chr1q41	0	x									
119	1554452_a_at	HIG2	chr7q32.1	0							x	x	x	
120	205266_at	LIF	chr22q12.2	0	x	x	x							
121	226210_s_at	MEG3	chr14q32	0				x	x					x
122	204914_s_at	SOX11	chr2p25	0	x									
123	230290_at	SCUBE3	chr6p21.3	0				x	x	x				x
124	224553_s_at	TNFRSF18	chr1p36.3	0				x	x	x				
125	226658_at	PDPN	chr1p36.21	0				x	x	x				
126	219998_at	HSPC159	chr2p14	0	x	x	x							x
127	1552288_at	---	---	0							x	x	x	x
128	235971_at	---	---	0				x						x
129	221085_at	TNFSF15	chr9q32	0	x									
130	211506_s_at	IL8	chr4q13-q21	0	x	x								x
131	202637_s_at	ICAM1	chr19p13.3-p13.2	0	x	x								x
132	1553300_a_at	DGKH	chr13q14.11	0							x			
133	231797_at	SIX4	chr14q23	0				x						
134	237945_at	LOC646590	chr14q32.31	0				x						
135	225292_at	COL27A1	chr9q32	0				x	x	x				x
136	206336_at	CXCL6	chr4q21	0	x									
137	218507_at	HIG2	chr7q32.1	0	x	x								
138	220241_at	TMCO3	chr13q34	0	x									
139	218484_at	NDUFA4L2	chr12q13.3	0	x	x								
140	206423_at	ANGPTL7	chr1p36.3-p36.2	0	x									

141	220254_at	LRP12	chr8q22.2-q23.1	0	x									
142	241592_at	---	---	0				x						x
143	221204_s_at	CRTAC1	chr10q22	0	x									
144	1552737_s_at	WWP2	chr16q22.1	0							x	x	x	
145	238858_at	TIFA	chr4q25	0				x						x
146	218469_at	GREM1	chr15q13-q15	0	x	x								x
147	233090_at	GPC6	chr13q32	0				x	x					x
148	219201_s_at	TWSG1	chr18p11.3	0	x									x
149	1557905_s_at	CD44 /// MAPK	chr11p13 /// chr4q22	0							x	x		
150	207154_at	DIO3	chr14q32	0	x									
151	219837_s_at	CYTL1	chr4p16-p15	0	x	x	x							
152	218810_at	ZC3H12A	chr1p34.3	0	x	x								x
153	239461_at	GALNTL2	chr3p24.3	0				x						
154	232771_at	NRK	chrXq22.3	0				x						
155	214954_at	SUSD5	chr3p22.3	0	x									
156	205207_at	IL6	chr7p21	0	x	x								x
157	244419_at	FRZB	chr2qter	0				x						x
158	231779_at	IRAK2	chr3p25.3	0				x	x	x				x
159	208025_s_at	HMGA2	chr12q15	0	x									
160	1559993_at	SFXN3	chr10q24.31	0							x	x	x	x
161	205503_at	PTPN14	chr1q32.2	0	x									
162	211564_s_at	PDLIM4	chr5q31.1	0	x	x								x
163	233039_at	SPRED1	chr15q14	0				x						
164	1552289_a_at	---	---	0							x	x	x	x
165	220504_at	KERA	chr12q22	0	x									
166	225681_at	CTHRC1	chr8q22.3	0				x	x					
167	203439_s_at	STC2	chr5q35.2	0	x	x								x
168	227452_at	LOC146795	chr17q24.3	0				x	x					
169	222877_at	NRP2	chr2q33.3	0				x	x					
170	228057_at	DDIT4L	chr4q23	0				x	x					x
171	1555675_at	BRCC2	chr11q24.1	0							x			
172	237215_s_at	TFRC	chr3q29	0				x						
173	241383_at	LOC201181	chr17q21.2	0				x	x	x				
174	223510_at	NRP2	chr2q33.3	0				x						
175	236982_at	---	---	0				x	x					x
176	229221_at	---	---	0				x	x	x				x
177	235106_at	MAML2	chr11q21	0				x						
178	1552364_s_at	MSI2	chr17q22	0							x	x	x	
179	211170_s_at	PDE10A	chr6q26	0	x									
180	226777_at	---	---	0				x						x
181	231766_s_at	COL12A1	chr6q12-q13	0				x	x					x
182	227390_at	MEG3	chr14q32	0				x						x
183	226834_at	---	---	0				x	x					x
184	242029_at	FNDC3B	chr3q26.31	0				x						x
185	242699_at	---	---	0				x						x
186	206307_s_at	FOXD1	chr5q12-q13	0	x									
187	224093_at	IFNK	---	0				x						
188	237496_at	PAPSS2	chr10q23-q24	0				x						
189	207850_at	CXCL3	chr4q21	0	x	x								
190	215286_s_at	PHTF2	chr7q11.23-q21	0	x									x

191	1557352_at	SQLE	chr8q24.1	0							x			x
192	1555527_at	COL9A1	chr6q12-q14	0							x			x
193	236947_at	SEMA3C	chr7q21-q31	0				x						x
194	220117_at	ZNF659	chr3p24.3	0	x									
195	209010_s_at	TRIO	chr5p15.1-p14	0	x									
196	231720_s_at	JAM3	chr11q25	0				x						x
197	235686_at	FLJ37953	chr2q33.1	0				x						
198	239787_at	KCTD4	chr13q14.12	0				x						
199	220240_s_at	TMCO3	chr13q34	0	x									
200	242064_at	SDK2	chr17q25.1	0				x	x					
201	207535_s_at	NFKB2	chr10q24	0	x									
202	219516_at	TRPV4	chr12q24.1	0	x									
203	210511_s_at	INHBA	chr7p15-p13	0	x	x								x
204	202709_at	FMOD	chr1q32	0	x									x
205	235831_at	---	---	0				x	x					x
206	224848_at	CDK6	chr7q21-q22	0				x	x					
207	206569_at	IL24	chr1q32	0	x									
208	219985_at	HS3ST3A1	chr17p12-p11.2	0	x									
209	244439_at	SPRED1	chr15q14	0				x						
210	209277_at	TFPI2	chr7q22	0	x									x
211	238796_at	YTHDC1	chr4q13.2	0				x						
212	214175_x_at	PDLIM4	chr5q31.1	0	x	x	x							x
213	243555_at	EDNRA	chr4q31.23	0				x	x	x				
214	1557123_a_at	RP4-756G23.1	chr22q13.2	0							x	x	x	
215	241780_at	CDH11	chr16q22.1	0				x						
216	207992_s_at	AMPD3	chr11p15	0	x									x
217	203325_s_at	COL5A1	chr9q34.2-q34.3	0	x	x								
218	221898_at	PDPN	chr1p36.21	0	x									
219	210916_s_at	CD44 /// MAPK	chr11p13 /// chr4q22	0	x	x								x
220	242856_at	---	---	0				x	x					x
221	233668_at	EDIL3	chr5q14	0				x	x	x				x
222	210881_s_at	IGF2 /// INS-IG	chr11p15.5	0	x	x								x
223	207822_at	FGFR1	chr8p11.2-p11.1	0	x									
224	201642_at	IFNGR2	chr21q22.11	0	x									
225	234073_at	---	---	0				x						x
226	1555778_a_at	POSTN	chr13q13.3	0							x	x		
227	222348_at	---	---	0	x	x	x							x
228	1560274_at	WTAP /// LOC	chr6q25-q27 /// chr6	0							x	x	x	
229	202267_at	LAMC2	chr1q25-q31	0	x									
230	241495_at	CCNL1	chr3q25.32	0				x						
231	223851_s_at	TNFRSF18	chr1p36.3	0				x						
232	1561073_at	TRPS1	chr8q24.12	0							x			x
233	1552439_s_at	MEGF11	chr15q22.31	0							x			
234	205796_at	TCP11L1	chr11p13	0	x									
235	209722_s_at	SERPINB9	chr6p25	0	x									
236	207361_at	HBP1	chr7q22-q31	0	x									
237	209545_s_at	RIPK2	chr8q21	0	x									
238	1561048_at	RARSL	chr6q16.1	0							x			
239	207510_at	BDKRB1	chr14q32.1-q32.2	0	x	x	x							
240	206002_at	GPR64	chrXp22.13	0	x									

241	223740_at	C6orf59	chr6q26	0				x						
242	210841_s_at	NRP2	chr2q33.3	0	x									
243	204470_at	CXCL1	chr4q21	0	x	x								
244	204596_s_at	STC1	chr8p21-p11.2	0	x									
245	233801_s_at	SEMA6D	chr15q21.1	0				x	x	x				
246	242100_at	CSS3	chr5q23.3	0				x						
247	1561394_s_at	RP5-1054A22.	chr20q11.23	0							x			
248	233222_at	GPC6	chr13q32	0				x						x
249	201250_s_at	SLC2A1	chr1p35-p31.3	0	x									
250	210405_x_at	TNFRSF10B	chr8p22-p21	0	x									x
251	230275_at	ARSI	chr5q33.1	0				x						
252	202966_at	CAPN6	chrXq23	0	x									
253	244841_at	SEC24A /// FL	chr5q31.1 /// chr15q2	0				x						
254	216005_at	TNC	chr9q33	0	x									x
255	203059_s_at	PAPSS2	chr10q23-q24	0	x	x								
256	207468_s_at	SFRP5	chr10q24.1	0	x									x
257	230357_at	GMDS	chr6p25	0				x						x
258	1568949_at	PITPNC1	chr17q24.2	0							x	x	x	x
259	233364_s_at	LOC399959	chr11q24.1	0				x	x					x
260	227145_at	LOXL4	chr10q24	0				x	x	x				
261	219973_at	ARSJ	chr4q26	0	x									
262	215209_at	SEC24D	chr4q26	0	x									
263	221959_at	C8orf72	chr8q12.1	0	x									
264	226211_at	MEG3	chr14q32	0				x	x					x
265	202434_s_at	CYP1B1	chr2p21	0	x									x
266	1556656_at	FMNL2	chr2q23.3	0							x			
267	212014_x_at	CD44	chr11p13	0	x									x
268	224507_s_at	MGC12916	chr17p11.2	0				x						
269	1568623_a_at	SLC35E4	chr22q12.2	0							x			
270	202997_s_at	LOXL2	chr8p21.3-p21.2	0	x									x
271	1554997_a_at	PTGS2	chr1q25.2-q25.3	0							x	x		x
272	229604_at	CMAH	chr6p21.32	0				x	x	x				
273	212488_at	COL5A1	chr9q34.2-q34.3	0	x	x								
274	1557637_at	PPP3CA	chr4q21-q24	0							x			
275	212902_at	SEC24A	chr5q31.1	0	x									x
276	221583_s_at	KCNMA1	chr10q22.3	0	x									
277	1555009_a_at	SYNJ2	chr6q25.3	0							x	x	x	x
278	205431_s_at	BMP5	chr6p12.1	0	x	x								
279	219523_s_at	ODZ3	chr4q35.1	0	x									
280	223019_at	C9orf88	chr9q34.11	0				x						
281	1559282_at	PLAGL1	chr6q24-q25	0							x			x
282	243000_at	CDK6	chr7q21-q22	0				x						
283	206942_s_at	PMCH	chr12q23-q24	0	x									
284	217428_s_at	COL10A1	chr6q21-q22	0	x	x	x							x
285	223321_s_at	FGFRL1	chr4p16	0				x						
286	233882_s_at	SEMA6D	chr15q21.1	0				x	x					
287	1567032_s_at	ZNF160	chr19q13.41	0							x			
288	205729_at	OSMR	chr5p13.1	0	x									x
289	232579_at	---	---	0				x						x
290	236361_at	GALNTL2	chr3p24.3	0				x	x	x				

291	220988_s_at	C1QTNF3	---	0	x	x	x							
292	204562_at	IRF4	chr6p25-p23	0	x									
293	214452_at	BCAT1	chr12pter-q12	0	x									
294	221730_at	COL5A2	chr2q14-q32	0	x	x								x
295	232090_at	DNM3	chr1q24.3	0				x						
296	220475_at	SLC28A3	chr9q22.2	0	x	x	x							
297	1557527_at	RUNX1	chr21q22.3	0							x	x	x	x
298	1560141_at	LOC642962	chr3p25.2	0							x			
299	234103_at	KCNT2	chr1q31.3	0				x	x	x				
300	1569490_at	FNDC3B	chr3q26.31	0							x			x
301	210367_s_at	PTGES	chr9q34.3	0	x									
302	203697_at	FRZB	chr2qter	0	x	x								x
303	212937_s_at	COL6A1	chr21q22.3	0	x									x
304	229796_at	SIX4	chr14q23	0				x						
305	1560739_a_at	UBE3C	chr7q36.3	0							x	x	x	
306	230773_at	---	---	0				x	x					x
307	201866_s_at	NR3C1	chr5q31.3	0	x	x								x
308	224767_at	RPL37	chr5p13	0				x						x
309	202332_at	CSNK1E	chr22q13.1	0	x									x
310	226368_at	CHST11	chr12q	0				x	x	x				x
311	209278_s_at	TFPI2	chr7q22	0	x	x								x
312	226824_at	CPXM2	chr10q26.13	0				x						x
313	224407_s_at	RP6-213H19.1	chrXq26.2	0				x	x	x				
314	209636_at	NFKB2	chr10q24	0	x									
315	206027_at	S100A3	chr1q21	0	x									
316	52651_at	COL8A2	chr1p34.2	0	x									
317	205870_at	BDKRB2	chr14q32.1-q32.2	0	x									
318	209835_x_at	CD44	chr11p13	0	x									x
319	202828_s_at	MMP14	chr14q11-q12	0	x									
320	233771_at	TRIO	chr5p15.1-p14	0				x						
321	241716_at	HSPD1	chr2q33.1	0				x						
322	203417_at	MFAP2	chr1p36.1-p35	0	x	x								
323	228481_at	POSTN	chr13q13.3	0				x						
324	215561_s_at	IL1R1	chr2q12	0	x									x
325	228923_at	S100A6	chr1q21	0				x						
326	233691_at	ABTB2	chr11p13	0				x						x
327	232472_at	FNDC3B	chr3q26.31	0				x	x	x				x
328	233106_at	C14orf82	chr14q22.1	0				x						
329	228667_at	AGPAT4	chr6q26	0				x	x					x
330	229242_at	---	---	0				x	x	x				x
331	1554835_a_at	B3GNT5	chr3q28	0							x	x	x	
332	210145_at	PLA2G4A	chr1q25	0	x									
333	228441_s_at	PET112L	chr4q27-q28	0				x	x	x				
334	201188_s_at	ITPR3	chr6p21	0	x									
335	209294_x_at	TNFRSF10B	chr8p22-p21	0	x									x
336	235407_at	---	---	0				x						x
337	204490_s_at	CD44	chr11p13	0	x									x
338	210861_s_at	WISP3	chr6q21	0	x									
339	1555963_x_at	B3GNT7	chr2q37.1	0							x	x	x	
340	208361_s_at	POLR3D	chr8q21	0	x									x

341	224847_at	CDK6	chr7q21-q22	0				x	x					
342	221900_at	COL8A2	chr1p34.2	0	x	x								
343	235705_at	TRIO	chr5p15.1-p14	0				x						x
344	210594_x_at	MPZL1	chr1q24.2	0	x									x
345	206026_s_at	TNFAIP6	chr2q23.3	0	x	x								x
346	215411_s_at	TRAF3IP2	chr6q21	0	x	x								x
347	219102_at	RCN3	chr19q13.33	0	x									
348	233109_at	COL12A1	chr6q12-q13	0				x						x
349	214095_at	SHMT2	chr12q12-q14	0	x									x
350	212489_at	COL5A1	chr9q34.2-q34.3	0	x	x								
351	231879_at	COL12A1	chr6q12-q13	0				x	x					x
352	1567224_at	HMGA2	chr12q15	0							x			
353	217430_x_at	COL1A1	chr17q21.33	0	x	x								x
354	205379_at	CBR3	chr21q22.2	0	x									x
355	241453_at	PTK2	chr8q24-qter	0				x	x	x				
356	203990_s_at	UTX	chrXp11.2	0	x									
357	210457_x_at	HMGA1	chr6p21	0	x									x
358	220634_at	TBX4	chr17q21-q22	0	x									
359	215672_s_at	KIAA0828	chr7q32.1	0	x									
360	219122_s_at	ICF45	chr5q33.3	0	x									
361	222942_s_at	TIAM2	chr6q25.2	0				x	x					x
362	211756_at	PTHLH	chr12p12.1-p11.2	0	x									
363	227345_at	TNFRSF10D	chr8p21	0				x	x	x				
364	237452_at	COL27A1	chr9q32	0				x						x
365	229790_at	TERF2	chr16q22.1	0				x						
366	217494_s_at	PTENP1	chr9p21	0	x									
367	232113_at	LOC399959	chr11q24.1	0				x	x					x
368	236829_at	TMF1	chr3p21-p12	0				x						
369	235911_at	MFI2	chr3q28-q29	0				x	x					
370	227371_at	BAIAP2L1	chr7q21.3	0				x	x					
371	244219_at	WTAP	chr6q25-q27	0				x	x	x				
372	202410_x_at	IGF2 /// INS-IG	chr11p15.5	0	x	x								
373	1555561_a_at	UGCGL2	chr13q32.1	0							x			
374	1569312_at	ZNF146	chr19q13.1	0							x	x	x	x
375	235868_at	MGEA5	chr10q24.1-q24.3	0				x						
376	210794_s_at	MEG3 /// LOC	chr14q32 /// chr14q3	0	x									
377	228501_at	GALNTL2	chr3p24.3	0				x	x					
378	230110_at	MCOLN2	chr1p22	0				x	x					
379	204345_at	COL16A1	chr1p35-p34	0	x									x
380	1566901_at	TGIF	chr18p11.3	0							x	x	x	x
381	1554474_a_at	MOXD1	chr6q23.1-23.3	0							x	x		x
382	40016_g_at	MAST4	chr5q12.3	0	x	x								x
383	213790_at	---	---	0	x									x
384	232636_at	SLITRK4	chrXq27.3	0				x	x					
385	208384_s_at	MID2	chrXq22	0	x									
386	223079_s_at	GLS	chr2q32-q34	0				x	x					
387	219936_s_at	GPR87	chr3q24	0	x									x
388	213696_s_at	MED8	chr1p34.2	0	x									
389	213167_s_at	SLC5A3	chr21q22.12	0	x									
390	205205_at	RELB	chr19q13.32	0	x	x	x							

391	213497_at	ABTB2	chr11p13	0	x	x							x
392	209220_at	GPC3	chrXq26.1	0	x	x							x
393	1561180_at	LRP11	chr6q25.1	0						x	x	x	
394	240145_at	DGKH	chr13q14.11	0			x						
395	205226_at	PDGFRL	chr8p22-p21.3	0	x	x							x
396	1555962_at	B3GNT7	chr2q37.1	0						x	x	x	
397	219155_at	PITPNC1	chr17q24.2	0	x								x
398	227044_at	TBC1D22A	chr22q13.3	0			x						
399	206173_x_at	GABPB2	chr15q21.2	0	x								
400	232668_at	C8orf72	chr8q12.1	0			x	x	x				
401	205579_at	HRH1	chr3p25	0	x								x
402	205903_s_at	KCNN3	chr1q21.3	0	x								
403	206571_s_at	MAP4K4	chr2q11.2-q12	0	x	x							
404	209396_s_at	CHI3L1	chr1q32.1	0	x	x							
405	221704_s_at	VPS37B	chr12q24.31	0	x								x
406	206025_s_at	TNFAIP6	chr2q23.3	0	x	x							x
407	1562208_a_at	SLC41A2	chr12q23.3	0						x	x	x	
408	232717_at	KALRN	chr3q21.1-q21.2	0			x						x
409	227816_at	NTN1	chr17p13-p12	0			x						x
410	209921_at	SLC7A11	chr4q28-q32	0	x								x
411	1559064_at	NUP153	chr6p22.3	0						x			x
412	215101_s_at	CXCL5	chr4q12-q13	0	x								
413	217584_at	NPC1	chr18q11-q12	0	x								
414	235392_at	IRS1	chr2q36	0			x	x					
415	218898_at	FAM57A	chr17p13.3	0	x								
416	213170_at	GPX7	chr1p32	0	x	x							
417	1565639_a_at	PMP22	chr17p12-p11.2	0						x			x
418	219332_at	MICAL-L2	chr7p22.3	0	x								
419	234932_s_at	CDCP1	chr3p21.31	0			x						x
420	223842_s_at	SCARA3	chr8p21	0			x						
421	207386_at	CYP7B1	chr8q21.3	0	x								
422	238803_at	HECTD2	chr10q23.32	0			x						
423	225288_at	---	---	0			x	x					x
424	213640_s_at	LOX	chr5q23.2	0	x								x
425	235311_at	FKBP14	chr7p15.1	0			x						
426	220134_x_at	C1orf78	chr1p34.3	0	x								x
427	202656_s_at	SERTAD2	chr2p14	0	x	x							x
428	215062_at	FMNL2	chr2q23.3	0	x								
429	204933_s_at	TNFRSF11B	chr8q24	0	x	x							x
430	213290_at	COL6A2	chr21q22.3	0	x								x
431	242457_at	PAM	chr5q14-q21	0			x						
432	220628_s_at	SDK2	chr17q25.1	0	x								
433	1554097_a_at	LOC554202	chr9p21.3	0						x			
434	1555875_at	---	---	0						x			x
435	212338_at	MYO1D	chr17q11-q12	0	x								
436	206506_s_at	SUPT3H	chr6p21.1-p21.3	0	x								
437	242176_at	MEF2A	chr15q26	0			x						
438	202679_at	NPC1	chr18q11-q12	0	x								
439	219390_at	FKBP14	chr7p15.1	0	x								
440	220512_at	DLC1	chr8p22	0	x								x

441	216915_s_at	PTPN12	chr7q11.23	0	x									
442	202619_s_at	PLOD2	chr3q23-q24	0	x	x								x
443	209321_s_at	ADCY3	chr2p24-p22	0	x									
444	214240_at	GAL	chr11q13.2	0	x									
445	236289_at	---	---	0				x	x	x				x
446	220658_s_at	ARNTL2	chr12p12.2-p11.2	0	x									
447	201981_at	PAPPA	chr9q33.2	0	x									x
448	242422_at	---	---	0				x						x
449	232535_at	---	---	0				x						x
450	208047_s_at	NAB1	chr2q32.3-q33	0	x									x
451	202855_s_at	SLC16A3	chr17q25	0	x	x								
452	230372_at	---	---	0				x						x
453	209708_at	MOXD1	chr6q23.1-23.3	0	x	x								x
454	1555199_at	GOSR1	chr17q11	0							x			
455	235952_at	DGKH	chr13q14.11	0				x						
456	244022_at	FNDC3B	chr3q26.31	0				x	x	x				x
457	215033_at	TM4SF1	chr3q21-q25	0	x									x
458	204420_at	FOSL1	chr11q13	0	x	x	x							
459	210538_s_at	BIRC3	chr11q22	0	x									
460	224218_s_at	TRPS1	chr8q24.12	0				x	x					x
461	229865_at	FNDC3B	chr3q26.31	0				x						x
462	235272_at	SBSN	chr19q13.13	0				x						
463	238933_at	---	---	0				x						x
464	216841_s_at	SOD2	chr6q25.3	0	x									x
465	220334_at	RGS17	chr6q25.3	0	x									
466	209716_at	CSF1	chr1p21-p13	0	x									
467	232816_s_at	DDX11 /// LOC	chr12p11 /// chr12p1	0				x						
468	223774_at	C1orf79	chr1p35.3	0				x						
469	1552931_a_at	PDE8A	chr15q25.3	0							x			
470	1555097_a_at	PTGFR	chr1p31.1	0							x	x	x	
471	217787_s_at	GALNT2	chr1q41-q42	0	x									
472	230867_at	LOC131873	chr3q22.1	0				x	x					
473	230380_at	THAP2	chr12q21.1	0				x						
474	206748_s_at	SPAG9	chr17q21.33	0	x									
475	205398_s_at	SMAD3	chr15q22.33	0	x									
476	203936_s_at	MMP9	chr20q11.2-q13.1	0	x	x								
477	214075_at	NENF	chr1q32.3	0	x									
478	204243_at	RLF	chr1p32	0	x	x	x							
479	211302_s_at	PDE4B	chr1p31	0	x	x								x
480	206987_x_at	FGF18	chr5q34	0	x									
481	232779_at	GMDS	chr6p25	0				x						x
482	235116_at	TRAF1	chr9q33-q34	0				x						
483	235756_at	SAMD4A	chr14q22.2	0				x						
484	226709_at	ROBO2	chr3p12.3	0				x						
485	202747_s_at	ITM2A	chrXq13.3-Xq21.2	0	x									x
486	240815_at	SEMA3C	chr7q21-q31	0				x						x
487	221710_x_at	C1orf78	chr1p34.3	0	x									x
488	228088_at	---	---	0				x						x
489	208502_s_at	PITX1	chr5q31	0	x									
490	243050_at	PMP22	chr17p12-p11.2	0				x						x

491	213816_s_at	MET	chr7q31	0	x								x
492	236144_at	CPXM2	chr10q26.13	0			x						x
493	202965_s_at	CAPN6	chrXq23	0	x	x							
494	204794_at	DUSP2	chr2q11	0	x								
495	1555785_a_at	XRN1	chr3q23	0						x			
496	224851_at	CDK6	chr7q21-q22	0			x	x					
497	209324_s_at	RGS16	chr1q25-q31	0	x	x							
498	215990_s_at	BCL6	chr3q27	0	x								x
499	232581_x_at	HIVEP3	chr1p34	0			x						
500	202952_s_at	ADAM12	chr10q26.3	0	x								
501	217100_s_at	UBXD7	chr3q29	0	x								
502	207012_at	MMP16	chr8q21	0	x								
503	218766_s_at	WARS2	chr1p13.3-p13.1	0	x								
504	235631_at	---	---	0			x						x
505	218978_s_at	SLC25A37	chr8p21.2	0	x								
506	212975_at	DENND3	chr8q24.3	0	x								
507	204864_s_at	IL6ST	chr5q11	0	x								x
508	212942_s_at	KIAA1199	chr15q24	0	x	x							
509	206036_s_at	REL	chr2p13-p12	0	x								
510	228906_at	CXXC6	chr10q21	0			x						
511	223730_at	GPC6	chr13q32	0			x	x	x				x
512	222806_s_at	C11orf30	chr11q13.5	0			x						
513	238009_at	---	---	0			x	x					x
514	213006_at	CEBPD	chr8p11.2-p11.1	0	x								x
515	228407_at	SCUBE3	chr6p21.3	0			x	x	x				x
516	1555253_at	COL25A1	chr4q25	0						x	x	x	
517	242436_at	PLEKHH2	chr2p21	0			x						
518	233888_s_at	SRGAP1	chr12q14.2	0			x						
519	227484_at	---	---	0			x						x
520	1555465_at	MCOLN2	chr1p22	0						x	x	x	
521	244570_at	---	---	0			x						x
522	1569706_at	MYSM1	chr1p32.1	0						x			
523	219634_at	CHST11	chr12q	0	x								x
524	213541_s_at	ERG	chr21q22.3	0	x								x
525	223192_at	SLC25A28	chr10q23-q24	0			x						
526	236105_at	---	---	0			x						x
527	226407_at	---	---	0			x						x
528	1566551_at	PDZRN3	chr3p13	0						x	x		
529	241460_at	RPS6KA3	chrXp22.2-p22.1	0			x	x	x				
530	201875_s_at	MPZL1	chr1q24.2	0	x								x
531	239266_at	C6orf160	chr6q14.3	0			x						
532	204760_s_at	THRA /// NR1I	chr17q11.2	0	x	x							
533	1565269_s_at	ATF1	chr12q13	0						x	x	x	
534	227415_at	LOC283508	chr13q13.3	0			x	x					
535	213035_at	ANKRD28	chr3p24.3	0	x								x
536	203927_at	NFKBIE	chr6p21.1	0	x	x							
537	203935_at	ACVR1	chr2q23-q24	0	x	x							x
538	240983_s_at	CARS	chr11p15.5	0			x						x
539	1560763_at	DIP2C	chr10p15.3	0						x	x		
540	238469_at	C6orf155	chr6q13	0			x						

541	211139_s_at	NAB1	chr2q32.3-q33	0	x								x
542	1561042_at	ITGB1	chr10p11.2	0						x	x	x	x
543	201189_s_at	ITPR3	chr6p21	0	x	x							
544	220738_s_at	RPS6KA6	chrXq21	0	x								
545	1559060_a_at	KIAA1961	chr5q23.3	0						x			
546	205397_x_at	SMAD3	chr15q22.33	0	x								
547	224792_at	TNKS1BP1	chr11q12.1	0			x						
548	230680_at	---	---	0			x	x					x
549	204668_at	RNF24	chr20p13-p12.1	0	x								
550	228750_at	COL14A1	chr8q23	0			x	x					x
551	1562606_a_at	LOC440028	chr11p15.4	0						x			
552	210612_s_at	SYNJ2	chr6q25.3	0	x								x
553	212865_s_at	COL14A1	chr8q23	0	x	x							x
554	226372_at	CHST11	chr12q	0			x	x	x				x
555	201783_s_at	RELA	chr11q13	0	x								x
556	214441_at	STX6	chr1q25.3	0	x								x
557	223991_s_at	GALNT2	chr1q41-q42	0			x						
558	241100_at	---	---	0			x						x
559	1562102_at	AKR1C2	chr10p15-p14	0						x	x	x	
560	204875_s_at	GMDS	chr6p25	0	x								x
561	205013_s_at	ADORA2A	chr22q11.23	0	x								
562	1559529_at	PTK2	chr8q24-qter	0						x	x	x	
563	208129_x_at	RUNX1	chr21q22.3	0	x								x
564	1569952_x_at	---	---	0						x			x
565	228355_s_at	NDUFA12L	chr5q12.1	0			x						
566	207604_s_at	SLC4A7	chr3p22	0	x								
567	204699_s_at	C1orf107	chr1q32.2	0	x								
568	205501_at	---	---	0	x								x
569	213562_s_at	SQLE	chr8q24.1	0	x								x
570	220153_at	ENTPD7	---	0	x								
571	229437_at	BIC	chr21q21.3	0			x	x	x				
572	229441_at	PRSS23	chr11q14.1	0			x						x
573	203072_at	MYO1E	chr15q21-q22	0	x								
574	211165_x_at	EPHB2	chr1p36.1-p35	0	x								
575	233400_at	CCDC82	chr11q21	0			x						
576	228587_at	LOC650803	---	0			x						
577	209180_at	RABGGTB	chr1p31	0	x								x
578	204833_at	ATG12	chr5q21-q22	0	x								x
579	204983_s_at	GPC4	chrXq26.1	0	x								
580	229795_at	---	---	0			x						x
581	210654_at	TNFRSF10D	chr8p21	0	x								
582	221423_s_at	YIPF5	chr5q32	0	x								x
583	37408_at	MRC2	chr17q23.2	0	x								
584	221778_at	KIAA1718	chr7q34	0	x								x
585	1555680_a_at	SMOX	chr20p13	0						x			x
586	231756_at	ZP4	chr1q43	0			x						
587	207492_at	NGLY1	chr3p24.2	0	x								
588	222223_s_at	IL1F5	chr2q14	0	x								
589	217678_at	SLC7A11	chr4q28-q32	0	x								x
590	216952_s_at	LMNB2	chr19p13.3	0	x								

591	205289_at	BMP2	chr20p12	0	x	x	x					x
592	207464_at	AHCYL1	chr1p13.2	0	x							
593	225867_at	VASN	chr16p13.3	0				x	x	x		x
594	207536_s_at	TNFRSF9	chr1p36	0	x							
595	217626_at	AKR1C2	chr10p15-p14	0	x	x	x					x
596	232653_at	TRPS1	chr8q24.12	0				x				x
597	1554543_at	SPAG9	chr17q21.33	0							x	
598	224739_at	PIM3	chr22q13	0				x				x
599	207388_s_at	PTGES	chr9q34.3	0	x							
600	203058_s_at	PAPSS2	chr10q23-q24	0	x	x						
601	204037_at	EDG2 /// LOC6	chr9q31.3	0	x							
602	236778_at	ATRX	chrXq13.1-q21.1	0				x				
603	219275_at	PDCD5	chr19q12-q13.1	0	x							
604	220326_s_at	FLJ10357	chr14q11.2	0	x							
605	1562307_at	RNF24	chr20p13-p12.1	0							x	
606	205114_s_at	CCL3 /// CCL3	chr17q11-q21 /// chr1	0	x	x						
607	1568900_a_at	ZNF568	chr19q13.12	0							x	
608	237169_at	---	---	0				x				x
609	228976_at	ICOSLG	chr21q22.3	0				x				
610	240407_at	---	---	0				x				x
611	242612_at	NPEPPS	chr17q21	0				x				
612	234894_at	ITIH5L	chrXp11.21	0				x				
613	1569449_a_at	CRSP2	chrXp11.4-p11.2	0							x	
614	227486_at	NT5E	chr6q14-q21	0				x				
615	239653_at	RTKN	chr2p13.1	0				x				x
616	213406_at	WSB1	chr17q11.1	0	x							x
617	222611_s_at	PSPC1	chr13q12.11	0				x				
618	221923_s_at	NPM1	chr5q35	0	x							x
619	212758_s_at	TCF8	chr10p11.2	0	x							
620	211485_s_at	FGF18	chr5q34	0	x							
621	227059_at	GPC6	chr13q32	0				x	x			x
622	235629_at	FN1	chr2q34	0				x	x	x		x
623	224396_s_at	ASPN	chr9q22	0				x				
624	242669_at	UFM1	chr13q13.3	0				x	x	x		x
625	241392_at	TMEM39A	chr3q13.33	0				x				
626	220574_at	SEMA6D	chr15q21.1	0	x							
627	219675_s_at	UXS1	chr2q12.2	0	x							
628	236028_at	IBSP	chr4q21-q25	0				x	x	x		
629	220750_s_at	LEPRE1	chr1p34.1	0	x							
630	228910_at	---	---	0				x	x	x		x
631	223614_at	C8orf57	chr8q21.3	0				x	x			x
632	39402_at	IL1B	chr2q14	0	x							x
633	239672_at	LOC399959	chr11q24.1	0				x	x	x		x
634	214701_s_at	FN1	chr2q34	0	x							x
635	221729_at	COL5A2	chr2q14-q32	0	x	x						x
636	213329_at	SRGAP2	chr1q32.1	0	x							
637	202323_s_at	ACBD3	chr1q42.12	0	x							
638	209395_at	CHI3L1	chr1q32.1	0	x							
639	203499_at	EPHA2	chr1p36	0	x							x
640	204621_s_at	NR4A2	chr2q22-q23	0	x							

641	233276_at	FBXL7	chr5p15.1	0				x						x
642	225613_at	MAST4	chr5q12.3	0				x	x	x				x
643	202657_s_at	SERTAD2	chr2p14	0	x	x								x
644	216056_at	CD44	chr11p13	0	x									x
645	215671_at	PDE4B	chr1p31	0	x									x
646	232711_at	PKNOX2	---	0				x						
647	203788_s_at	SEMA3C	chr7q21-q31	0	x									x
648	202013_s_at	EXT2	chr11p12-p11	0	x									x
649	241272_at	TNC	chr9q33	0				x						x
650	212013_at	PXDN	chr2p25	0	x	x								x
651	225142_at	KIAA1718	chr7q34	0				x	x	x				x
652	1569854_at	QKI	chr6q26-q27	0							x			x
653	219239_s_at	ZNF654	chr3p11.2	0	x									
654	202859_x_at	IL8	chr4q13-q21	0	x	x								x
655	202610_s_at	CRSP2	chrXp11.4-p11.2	0	x									
656	204970_s_at	MAFG	chr17q25.3	0	x									
657	215223_s_at	SOD2	chr6q25.3	0	x	x								x
658	1554783_s_at	ARHGEF2	chr1q21-q22	0							x			
659	1552485_at	LACTB	chr15q22.1	0							x	x	x	
660	242243_at	TMF1	chr3p21-p12	0				x						
661	210557_x_at	CSF1	chr1p21-p13	0	x									
662	222344_at	C5orf13	chr5q22.1	0	x									x
663	218801_at	UGCGL2	chr13q32.1	0	x									
664	239439_at	AFF4	chr5q31	0				x						
665	219845_at	BARX1	chr9q12	0	x									
666	1562948_at	SMC5L1	chr9q21.11	0							x			
667	228463_at	FOXA3	chr19q13.2-q13.4	0				x						
668	223798_at	SLC41A2	chr12q23.3	0				x						
669	212091_s_at	COL6A1	chr21q22.3	0	x	x								x
670	204310_s_at	NPR2	chr9p21-p12	0	x									
671	207595_s_at	BMP1	chr8p21	0	x									
672	231274_s_at	---	---	0				x	x					x
673	240557_at	TSC22D2	chr3q25.1	0				x						
674	223018_at	NOB1	chr16q22.3	0				x						
675	232204_at	EBF	chr5q34	0				x						
676	216202_s_at	SPTLC2	chr14q24.3-q31	0	x									
677	210087_s_at	MPZL1	chr1q24.2	0	x									x
678	234978_at	SLC36A4	chr11q21	0				x						
679	212926_at	SMC5	chr9q21.11	0	x									
680	205396_at	SMAD3	chr15q22.33	0	x									
681	226766_at	ROBO2	chr3p12.3	0				x	x					
682	225963_at	KLHDC5	chr12p11.22	0				x						
683	1558143_a_at	BCL2L11	chr2q13	0							x	x	x	
684	205941_s_at	COL10A1	chr6q21-q22	0	x	x	x							x
685	244038_at	WDR89	chr14q23.2	0				x						
686	209359_x_at	RUNX1	chr21q22.3	0	x									x
687	211804_s_at	CDK2 /// BCD(	chr12q13 /// chr11q2	0	x									
688	210089_s_at	LAMA4	chr6q21	0	x									
689	207031_at	BAPX1	chr4p16.1	0	x									
690	1552277_a_at	MGC17337	chr9q31.1	0							x	x	x	

691	232354_at	VPS37B	chr12q24.31	0				x						x
692	234122_at	HNRPA1	chr12q13.1	0				x						x
693	220407_s_at	TGFB2	chr1q41	0	x									
694	225285_at	BCAT1	chr12pter-q12	0				x	x					
695	206157_at	PTX3	chr3q25	0	x	x								x
696	239295_at	SRrp35	chr6q15	0				x						
697	203823_at	RGS3	chr9q32	0	x									x
698	203530_s_at	STX4	chr16p11.2	0	x									
699	230850_at	FMNL3	chr12q13.12	0				x						x
700	207196_s_at	TNIP1	chr5q32-q33.1	0	x									
701	201747_s_at	SAFB	chr19p13.3-p13.2	0	x									
702	210651_s_at	EPHB2	chr1p36.1-p35	0	x									
703	219081_at	ANKHD1	chr5q31.3	0	x									
704	1554539_a_at	RHOF	chr12q24.31	0							x	x	x	
705	1555434_a_at	SLC39A14	chr8p21.3	0							x	x		x
706	232458_at	COL3A1	chr2q31	0				x						
707	204601_at	N4BP1	chr16q12.1	0	x									
708	242210_at	ZNF24	chr18q12	0				x						x
709	206869_at	CHAD	chr17q21.33	0	x									x
710	203068_at	KLHL21	chr1p36.31	0	x									x
711	240399_at	TRIO	chr5p15.1-p14	0				x						
712	232716_at	EDG2	chr9q31.3	0				x	x	x				
713	226676_at	ZNF521	chr18q11.2	0				x						
714	207626_s_at	SLC7A2	chr8p22-p21.3	0	x									
715	223196_s_at	SESN2	chr1p35.3	0				x						x
716	209561_at	THBS3	chr1q21	0	x	x	x							
717	209239_at	NFKB1	chr4q24	0	x	x								x
718	211668_s_at	PLAU	chr10q24	0	x									
719	209909_s_at	---	---	0	x									x
720	238729_x_at	LOC646561	chr4q31.22	0				x						
721	226188_at	HSPC159	chr2p14	0				x	x					x
722	1554638_at	ZFYVE16	chr5p15.2-q14.3	0							x	x	x	
723	210285_x_at	WTAP	chr6q25-q27	0	x									
724	206074_s_at	HMGA1	chr6p21	0	x									x
725	212722_s_at	PTDSR	chr17q25	0	x									x
726	65588_at	LOC388796	chr20q11.23	0	x									x
727	1556744_a_at	---	---	0							x	x		x
728	202012_s_at	EXT2	chr11p12-p11	0	x									x
729	241903_at	KCTD3	chr1q41	0				x						
730	209560_s_at	DLK1	chr14q32	0	x	x								x
731	1558517_s_at	LRRC8C	chr1p22.2	0							x	x	x	
732	211401_s_at	FGFR2	chr10q26	0	x									x
733	240450_at	PAPPA	chr9q33.2	0				x						x
734	236438_at	RABGEF1	chr7q11.21	0				x						x
735	214761_at	ZNF423	chr16q12	0	x	x								
736	220768_s_at	CSNK1G3	chr5q23	0	x									
737	213112_s_at	SQSTM1	chr5q35	0	x									x
738	223143_s_at	C6orf166	chr6q15	0				x						
739	1560813_at	FBXL7	chr5p15.1	0							x			x
740	224915_x_at	TALDO1 /// C2	chr11p15.5-p15.4 ///	0				x	x					

741	208693_s_at	GARS	chr7p15	0	x									x
742	216977_x_at	SNRPA1	chr15q26.3	0	x									x
743	222690_s_at	TMEM39A	chr3q13.33	0				x						
744	227971_at	NRK	chrXq22.3	0				x	x					
745	239654_at	CHD9	chr16q12.2	0				x						
746	203939_at	NT5E	chr6q14-q21	0	x									
747	222692_s_at	FNDC3B	chr3q26.31	0				x	x	x				x
748	1554863_s_at	DOK5	chr20q13.2	0							x	x		
749	232061_at	SDK2	chr17q25.1	0				x						
750	230440_at	ZNF469	chr16q24	0				x						
751	219479_at	KDELC1	chr13q33	0	x									
752	244533_at	---	---	0				x	x	x				x
753	221432_s_at	SLC25A28	chr10q23-q24	0	x									
754	213120_at	KIAA0701	chr12q23.1	0	x									x
755	222846_at	RAB8B	chr15q22.2	0				x						
756	214649_s_at	MTMR2	chr11q22	0	x									x
757	204422_s_at	FGF2	chr4q26-q27	0	x									x
758	1552625_a_at	TRNT1	---	0							x			x
759	1555562_a_at	ZCCHC7	chr9p13.2	0							x			
760	218319_at	PELI1	chr2p13.3	0	x									x
761	202310_s_at	COL1A1	chr17q21.33	0	x	x								x
762	234985_at	---	---	0				x						x
763	244579_at	TRPS1	chr8q24.12	0				x	x					x
764	206007_at	PRG4	chr1q25-q31	0	x									
765	242335_at	SLC25A37	chr8p21.2	0				x	x	x				
766	1565579_at	---	---	0							x			x
767	205599_at	TRAF1	chr9q33-q34	0	x									
768	1565651_at	ARF1	chr1q42	0							x	x	x	x
769	1554697_at	ADAMTS9	chr3p14.3-p14.2	0							x	x	x	
770	215722_s_at	SNRPA1	chr15q26.3	0	x									x
771	219474_at	C3orf52	chr3q13.2	0	x									x
772	235065_at	---	---	0				x						x
773	52255_s_at	COL5A3	chr19p13.2	0	x									x
774	210706_s_at	RNF24	chr20p13-p12.1	0	x	x	x							
775	205100_at	GFPT2	chr5q34-q35	0	x	x								x
776	214437_s_at	SHMT2	chr12q12-q14	0	x	x								x
777	239876_at	NFKB1	chr4q24	0				x						x
778	1553252_a_at	BRWD3	chrXq21.1	0							x			
779	210958_s_at	MAST4	chr5q12.3	0	x									x
780	212800_at	STX6	chr1q25.3	0	x									x
781	235963_at	EPAS1	chr2p21-p16	0				x						x
782	203045_at	NINJ1	chr9q22	0	x	x								
783	225699_at	C7orf40	chr7p13	0				x						
784	239258_at	RHOQ	chr2p21	0				x	x	x				
785	235012_at	LRCH1	chr13q14.13-q14.2	0				x						x
786	204748_at	PTGS2	chr1q25.2-q25.3	0	x	x								x
787	236375_at	TCEA1	chr8q11.2	0				x						x
788	205425_at	HIP1	chr7q11.23	0	x									
789	204595_s_at	STC1	chr8p21-p11.2	0	x	x								
790	218508_at	DCP1A	chr3p21.1	0	x									

791	205574_x_at	BMP1	chr8p21	0	x								
792	232748_at	PAPPA	chr9q33.2	0			x						x
793	229900_at	CD109	chr6q13	0			x						
794	236179_at	---	---	0			x						x
795	222101_s_at	DCHS1	chr11p15.4	0	x								
796	225007_at	---	---	0			x						x
797	213118_at	KIAA0701	chr12q23.1	0	x								x
798	1554339_a_at	COG3	chr13q14.12	0						x			x
799	225664_at	COL12A1	chr6q12-q13	0			x	x					x
800	203708_at	PDE4B	chr1p31	0	x	x							x
801	205578_at	ROR2	chr9q22	0	x								
802	223987_at	CHRDL2	chr11q14	0			x						
803	227526_at	CDON	chr11q23-q24	0			x						
804	234730_s_at	RIPK4	chr21q22.3	0			x						
805	228468_at	MASTL	chr10p12.1	0			x						
806	210783_x_at	CLEC11A	chr19q13.3	0	x								
807	237492_at	PDE10A	chr6q26	0			x						
808	218340_s_at	UBE1L2	chr4q13.2	0	x								
809	1554473_at	SRGAP1	chr12q14.2	0						x	x	x	
810	243502_at	GJA7	chr17q21.31	0			x						
811	212641_at	HIVEP2	chr6q23-q24	0	x	x							
812	1554726_at	ZNF655	chr7q22.1	0						x	x		
813	1552274_at	PXK	chr3p14.3	0						x			
814	203184_at	FBN2	chr5q23-q31	0	x	x							
815	219218_at	BAHCC1	chr17q25.3	0	x								
816	208803_s_at	SRP72	chr4q11	0	x								
817	226227_x_at	TALDO1 /// C2	chr11p15.5-p15.4 ///	0			x						
818	237460_x_at	LOC283551	chr14q22.1	0			x						
819	226621_at	---	---	0			x	x					x
820	229307_at	ANKRD28	chr3p24.3	0			x	x	x				x
821	233303_at	UBE2D3	chr4q24	0			x						x
822	217097_s_at	PHTF2	chr7q11.23-q21	0	x								x
823	231721_at	JAM3	chr11q25	0			x						x
824	205681_at	BCL2A1	chr15q24.3	0	x								
825	232004_at	HNRPR	chr1p36.12	0			x						x
826	210056_at	RND1	chr12q12-q13	0	x								
827	39548_at	NPAS2	chr2q11.2	0	x								
828	204384_at	GOLGA2	chr9q34.11	0	x								
829	201261_x_at	BGN	chrXq28	0	x								x
830	243388_at	SLC25A32	chr8q22.3	0			x						
831	232451_at	---	---	0			x						x
832	223533_at	LRRC8C	chr1p22.2	0			x						
833	206631_at	PTGER2	chr14q22	0	x								x
834	227368_at	C6orf166	chr6q15	0			x						
835	222326_at	PDE4B	chr1p31	0	x								x
836	232365_at	SIAH1	chr16q12	0			x						x
837	1555830_s_at	FAM62B	chr7q36.3	0						x	x	x	
838	1566191_at	SUZ12	chr17q11.2	0						x			
839	1569106_s_at	SETD5	chr3p25.3	0						x	x	x	
840	223271_s_at	CTDSPL2	chr15q15.3	0			x						

841	234351_x_at	TRPS1	chr8q24.12	0			x	x	x				x
842	217791_s_at	ALDH18A1	chr10q24.3	0	x								x
843	1569387_at	ChGn	chr8p21.3	0						x	x	x	
844	206632_s_at	APOBEC3B	chr22q13.1-q13.2	0	x								
845	204776_at	THBS4	chr5q13	0	x	x							
846	204507_s_at	PPP3R1	chr2p15	0	x								
847	205548_s_at	BTG3	chr21q21.1-q21.2	0	x	x							x
848	220712_at	C8orf60	chr8q24.3	0	x								
849	202570_s_at	DLGAP4	chr20q11.23	0	x								x
850	203476_at	TPBG	chr6q14-q15	0	x	x							x
851	213139_at	SNAI2	chr8q11	0	x	x							x
852	218379_at	RBM7	chr11q23.1-q23.2	0	x								x
853	221903_s_at	CYLD	chr16q12.1	0	x	x							
854	1563468_at	ZAK	chr2q24.2	0						x			
855	212658_at	LHFPL2	chr5q14.1	0	x								
856	241438_at	RLF	chr1p32	0			x						
857	202728_s_at	LTBP1	chr2p22-p21	0	x	x							x
858	211171_s_at	PDE10A	chr6q26	0	x								
859	225119_at	CHMP4B	chr20q11.22	0			x						
860	1553613_s_at	FOXC1	chr6p25	0						x	x		x
861	239451_at	HSP90B1	chr12q24.2-q24.3	0			x						
862	221291_at	ULBP2	chr6q25	0	x								
863	202510_s_at	TNFAIP2	chr14q32	0	x	x							
864	226835_s_at	TALDO1 /// C2	chr11p15.5-p15.4 ///	0			x	x					
865	210845_s_at	PLAUR	chr19q13	0	x								x
866	223575_at	KIAA1549	chr7q34	0			x						
867	203665_at	HMOX1	chr22q12|22q13.1	0	x	x							x
868	223834_at	CD274	chr9p24	0			x						
869	210350_x_at	ING1	chr13q34	0	x								
870	205580_s_at	HRH1	chr3p25	0	x								x
871	215641_at	SEC24D	chr4q26	0	x								
872	230258_at	GLIS3	chr9p24.2	0			x	x	x				
873	222528_s_at	SLC25A37	chr8p21.2	0			x	x					
874	240258_at	ENO1	chr1p36.3-p36.2	0			x						x
875	214319_at	FRY	chr13q13.1	0	x								
876	33304_at	ISG20	chr15q26	0	x	x							
877	212022_s_at	MKI67	chr10q25-qter	0	x								
878	211924_s_at	PLAUR	chr19q13	0	x								x
879	229540_at	RBPSUH /// L(	chr4p15.2	0			x						x
880	229814_at	---	---	0			x						x
881	218975_at	COL5A3	chr19p13.2	0	x								x
882	213159_at	PCNX	chr14q24.2	0	x								
883	209372_x_at	TUBB2A /// TU	chr6p25	0	x								
884	210805_x_at	RUNX1	chr21q22.3	0	x								x
885	238389_s_at	---	---	0			x						x
886	209747_at	TGFB3	chr14q24	0	x								
887	238735_at	TCF12	chr15q21	0			x						
888	229991_s_at	SYTL4	---	0			x						
889	235874_at	PRSS35	chr6q14.2	0			x						
890	235016_at	REEP3	chr10q21.3	0			x						

891	205854_at	TULP3	chr12p13.3	0	x							
892	220606_s_at	C17orf48	chr17p13.1	0	x							
893	222164_at	FGFR1	chr8p11.2-p11.1	0	x							
894	215092_s_at	NFAT5	chr16q22.1	0	x							
895	205459_s_at	NPAS2	chr2q11.2	0	x							
896	224942_at	PAPPA	chr9q33.2	0			x					x
897	1559862_at	COPA	chr1q23-q25	0						x		
898	229487_at	---	---	0			x					x
899	232693_s_at	ZNF395 /// FB	chr8p21.1	0			x					
900	230864_at	MGC42105	chr5p12	0			x					
901	212670_at	ELN	chr7q11.23	0	x							
902	218472_s_at	PELO	chr5q11.2	0	x	x						
903	203553_s_at	MAP4K5	chr14q11.2-q21	0	x							
904	1556332_at	---	---	0						x		x
905	202643_s_at	TNFAIP3	chr6q23	0	x	x						x
906	202827_s_at	MMP14	chr14q11-q12	0	x	x						
907	209927_s_at	C1orf77	chr1q21.3	0	x							
908	239058_at	FOXC2	chr16q22-16q24	0			x					
909	203843_at	RPS6KA3	chrXp22.2-p22.1	0	x							
910	209491_s_at	AMPD3 /// PP	chr11p15 /// chr22q1	0	x							
911	203904_x_at	CD82	chr11p11.2	0	x							x
912	232916_at	UBE2E2	chr3p24.2	0			x					
913	201645_at	TNC	chr9q33	0	x	x						x
914	230002_at	CLCC1	chr1p13.3	0			x					x
915	1552508_at	KCNE4	chr2q36.3	0						x		
916	209307_at	SWAP70	chr11p15	0	x							
917	221571_at	TRAF3	chr14q32.32	0	x							
918	202503_s_at	KIAA0101	chr15q22.31	0	x							
919	201502_s_at	NFKBIA	chr14q13	0	x							x
920	233442_at	PDZRN3	chr3p13	0			x					
921	1554053_at	SPTLC1	chr9q22.2	0						x		
922	217455_s_at	SSTR2	chr17q24	0	x							
923	207517_at	LAMC2	chr1q25-q31	0	x							
924	203125_x_at	SLC11A2	chr12q13	0	x							
925	205430_at	BMP5	chr6p12.1	0	x							
926	218360_at	RAB22A	chr20q13.32	0	x							
927	204618_s_at	GABPB2	chr15q21.2	0	x							
928	210882_s_at	TRO	chrXp11.22-p11.21	0	x							
929	232213_at	PELI1	chr2p13.3	0			x	x				x
930	1555800_at	ZNF533	chr2q31.2-q31.3	0						x	x	
931	244470_at	RNF12	chrXq13-q21	0			x					
932	230438_at	TBX15	chr1p11.1	0			x	x	x			x
933	225077_at	LOC283680	chr15q26.1	0			x					
934	214106_s_at	GMDS	chr6p25	0	x							x
935	200999_s_at	CKAP4	chr12q23.3	0	x							x
936	214374_s_at	PPFIBP1	chr12p11.23-p11.22	0	x							x
937	228914_at	---	---	0			x					x
938	205123_s_at	TMEFF1	chr9q31	0	x							
939	210048_at	NAPG	chr18p11.22	0	x							x
940	224453_s_at	ETNK1	chr12p12.1	0			x					

941	204285_s_at	PMAIP1	chr18q21.32	0	x	x							x
942	222874_s_at	CLN8	chr8p23	0				x					
943	202805_s_at	ABCC1	chr16p13.1	0	x								
944	201125_s_at	ITGB5	chr3q21.2	0	x	x							x
945	224610_at	STX5	chr11q12.3	0				x					x
946	1558783_at	WTAP	chr6q25-q27	0						x	x	x	
947	235549_at	IBRDC2	chr6p22.3	0				x					
948	210180_s_at	SFRS10	chr3q26.2-q27	0	x								x
949	227372_s_at	BAIAP2L1	chr7q21.3	0				x	x				
950	224941_at	PAPPA	chr9q33.2	0				x	x				x
951	235190_at	CALM2	chr2p21	0				x					x
952	224325_at	FZD8	chr10p11.21	0				x					
953	227751_at	PDCD5	chr19q12-q13.1	0				x					
954	204908_s_at	BCL3	chr19q13.1-q13.2	0	x	x							
955	202132_at	WWTR1	chr3q23-q24	0	x								
956	230640_at	PRPF40B	chr12q	0				x					
957	223641_at	MIA3	chr1q41	0				x					
958	227405_s_at	FZD8	chr10p11.21	0				x					
959	236883_at	TMEM77	chr1p13.3	0				x					
960	211067_s_at	GAS7	chr17p13.1	0	x	x							x
961	223836_at	KSP37	chr4p16	0				x	x				
962	206221_at	RASA3	chr13q34	0	x								
963	203099_s_at	CDYL	chr6p25.1	0	x								
964	203492_x_at	CEP57	chr11q21	0	x								
965	239930_at	GALNT2	chr1q41-q42	0				x					
966	242321_at	---	---	0				x					x
967	202667_s_at	SLC39A7	chr6p21.3	0	x								
968	1552275_s_at	PXK	chr3p14.3	0						x	x		
969	238794_at	C10orf78	chr10q25.1	0				x					
970	243012_at	RAPH1	chr2q33	0				x					x
971	209878_s_at	RELA	chr11q13	0	x	x	x						x
972	212626_x_at	HNRPC	chr14q11.2	0	x								x
973	218193_s_at	GOLT1B	chr12p12.1	0	x								
974	226517_at	BCAT1	chr12pter-q12	0				x	x				
975	1559296_at	---	---	0						x			x
976	1558561_at	HM13	chr20q11.21	0						x			
977	225293_at	COL27A1	chr9q32	0				x	x				x
978	219357_at	GTPBP1	chr22q13.1	0	x								
979	223984_s_at	NUPL1	chr13q12.13	0				x					x
980	215163_at	---	---	0	x								x
981	224950_at	PTGFRN	chr1p13.1	0				x					x
982	212828_at	SYNJ2	chr6q25.3	0	x	x							x
983	210357_s_at	SMOX	chr20p13	0	x								x
984	240370_at	PKD2	chr4q21-q23	0				x					
985	201655_s_at	HSPG2	chr1p36.1-p34	0	x								
986	210230_at	---	---	0	x								x
987	222142_at	CYLD	chr16q12.1	0	x								
988	209325_s_at	RGS16	chr1q25-q31	0	x								
989	232961_at	DNAPTP6	chr2q33.1	0				x					x
990	201892_s_at	IMPDH2	chr3p21.2	0	x								x

991	226545_at	CD109	chr6q13	0			x	x					
992	223503_at	TMEM163	chr2q21.3	0			x	x					
993	201469_s_at	SHC1	chr1q21	0	x								x
994	224574_at	C17orf49 /// M	chr17p13.1	0			x						
995	205905_s_at	MICA /// MICB	chr6p21.3	0	x								
996	224469_s_at	C14orf151	chr14q32.33	0			x						
997	211312_s_at	WISP1	chr8q24.1-q24.3	0	x								
998	238893_at	LOC338758	chr12q21.33	0			x	x	x				
999	212531_at	LCN2	chr9q34	0	x								
1000	235540_at	---	---	0			x						x
1001	225464_at	FRMD6	chr14q22.1	0			x	x					x
1002	208044_s_at	PPARD	chr6p21.2-p21.1	0	x								x
1003	235749_at	UGCGL2	chr13q32.1	0			x						
1004	1553603_s_at	ARL6IP2	chr2p22.2-p22.1	0						x			
1005	227721_at	CPAMD8	chr19p13.11	0			x						
1006	227262_at	HAPLN3	chr15q26.1	0			x						
1007	204698_at	ISG20	chr15q26	0	x	x							
1008	222527_s_at	RBM22	chr5q33.1	0			x						
1009	218561_s_at	C6orf149	chr6p25.1	0	x								
1010	216976_s_at	RYK	chr3q22	0	x								x
1011	212344_at	SULF1	chr8q13.2-q13.3	0	x								x
1012	244457_at	ITPR2	chr12p11	0			x	x					
1013	214446_at	ELL2	chr5q15	0	x	x							x
1014	1557257_at	BCL10	chr1p22	0						x	x	x	
1015	206552_s_at	TAC1	chr7q21-q22	0	x								
1016	214460_at	LSAMP	chr3q13.2-q21	0	x								
1017	1564746_at	LOC133308	chr4q24	0						x	x		
1018	203123_s_at	SLC11A2	chr12q13	0	x								
1019	209318_x_at	PLAGL1	chr6q24-q25	0	x	x							x
1020	202756_s_at	GPC1	chr2q35-q37	0	x								
1021	221477_s_at	MGC5618	---	0	x								x
1022	202465_at	PCOLCE	chr7q22	0	x	x							x
1023	218815_s_at	TMEM51	chr1p36.21	0	x								x
1024	205460_at	NPAS2	chr2q11.2	0	x								
1025	1565868_at	CD44	chr11p13	0						x	x	x	x
1026	227844_at	FMNL3	chr12q13.12	0			x						x
1027	237056_at	INSC	chr11p15.2	0			x						
1028	222863_at	ZBTB10	chr8q13-q21.1	0			x						
1029	201169_s_at	BHLHB2	chr3p26	0	x	x							
1030	203026_at	ZBTB5	chr9p13.2	0	x	x							
1031	201295_s_at	WSB1 /// LOC	chr17q11.1	0	x								
1032	224898_at	WDR26	chr1q42.11	0			x						
1033	209012_at	TRIO	chr5p15.1-p14	0	x								
1034	211535_s_at	FGFR1	chr8p11.2-p11.1	0	x								
1035	226552_at	IER5L	chr9q34.11	0			x						
1036	234976_x_at	SLC4A5	chr2p13	0			x	x	x				x
1037	212504_at	DIP2C	chr10p15.3	0	x	x							
1038	202435_s_at	CYP1B1	chr2p21	0	x								x
1039	209212_s_at	KLF5	chr13q22.1	0	x								x
1040	218875_s_at	FBXO5	chr6q25-q26	0	x								x

1041	205240_at	GPSM2	chr1p13.3	0	x								x
1042	242726_at	ACSL3	chr2q34-q35	0				x					x
1043	229557_at	MEG3	chr14q32	0				x					x
1044	204489_s_at	CD44	chr11p13	0	x								x
1045	1555317_at	POLK	chr5q13	0						x			x
1046	243213_at	STAT3	chr17q21.31	0				x					x
1047	218854_at	SART2	chr6q22	0	x	x							x
1048	1554464_a_at	CRTAP	chr3p22.3	0						x	x		
1049	226311_at	---	---	0				x					x
1050	204114_at	NID2	chr14q21-q22	0	x	x							x
1051	214597_at	SSTR2	chr17q24	0	x								
1052	203502_at	BPGM	chr7q31-q34	0	x								
1053	1557538_at	THRAP2	chr12q24.21	0						x			
1054	1553227_s_at	BRWD1	chr21q22.2	0						x			
1055	41037_at	TEAD4	chr12p13.2-p13.3	0	x								
1056	1555420_a_at	KLF7	chr2q32	0						x	x	x	
1057	242797_x_at	ZFR	chr5p13.3	0				x					x
1058	225301_s_at	MYO5B	chr18q21	0				x	x				
1059	222263_at	SLC35E1	chr19p13.11	0	x								x
1060	235548_at	APCDD1L	chr20q13.32	0				x					
1061	202369_s_at	TRAM2	chr6p21.1-p12	0	x	x							x
1062	244597_at	DNAPTP6	chr2q33.1	0				x					x
1063	220253_s_at	LRP12	chr8q22.2-q23.1	0	x								
1064	225796_at	PXK	chr3p14.3	0				x	x				
1065	206941_x_at	SEMA3E	chr7q21.11	0	x								
1066	211180_x_at	RUNX1	chr21q22.3	0	x								x
1067	214845_s_at	CALU	chr7q32	0	x								x
1068	210994_x_at	TRIM23	chr5q12.3	0	x								
1069	205475_at	SCRG1	chr4q31-q32	0	x	x							
1070	205479_s_at	PLAU	chr10q24	0	x	x							
1071	213310_at	EIF2C2	chr8q24	0	x	x	x						x
1072	224025_s_at	ATG7	chr3p25.3-p25.2	0				x					
1073	213909_at	LRRC15	chr3q29	0	x								x
1074	207570_at	SHOX	chrXpter-p22.32;Yp1	0	x								
1075	1569269_s_at	SRGAP1	chr12q14.2	0						x			
1076	223121_s_at	SFRP2	chr4q31.3	0				x					
1077	213338_at	TMEM158	chr3p21.3	0	x								
1078	228699_at	NRP2	chr2q33.3	0				x					
1079	224940_s_at	PAPPA	chr9q33.2	0				x	x				x
1080	230505_at	LOC145474	chr14q24.1	0				x					
1081	204286_s_at	PMAIP1	chr18q21.32	0	x								x
1082	205067_at	IL1B	chr2q14	0	x								x
1083	219197_s_at	SCUBE2	chr11p15.3	0	x								
1084	207079_s_at	MED6	chr14q24.2	0	x								
1085	205376_at	INPP4B	chr4q31.21	0	x								x
1086	1559362_at	KIAA0999	chr11q23.3	0						x			x
1087	230728_at	---	---	0				x					x
1088	234863_x_at	FBXO5	chr6q25-q26	0				x					x
1089	215357_s_at	POLDIP3	chr22q13.2	0	x								x
1090	202458_at	PRSS23	chr11q14.1	0	x	x							x

1091	202223_at	STT3A	chr11q23.3	0	x							
1092	204924_at	TLR2	chr4q32	0	x							
1093	211671_s_at	NR3C1	chr5q31.3	0	x							x
1094	226542_at	---	---	0			x					x
1095	210809_s_at	POSTN	chr13q13.3	0	x	x						
1096	214866_at	PLAUR	chr19q13	0	x							x
1097	214697_s_at	ROD1	chr9q32	0	x							
1098	201761_at	MTHFD2	chr2p13.1	0	x	x						x
1099	209862_s_at	CEP57	chr11q21	0	x							
1100	204119_s_at	ADK	chr10q22|10q11-q24	0	x	x						
1101	207920_x_at	ZFX	chrXp21.3	0	x							
1102	215513_at	HYMAI	chr6q24	0	x							
1103	242542_at	PHACTR2	chr6q24.2	0			x					
1104	225541_at	RPL22L1 /// L(	chr3q26.2	0			x	x				
1105	231576_at	ETNK1	chr12p12.1	0			x					
1106	232007_at	AGPAT5	chr8p23.1	0			x					x
1107	222675_s_at	BAIAP2L1	chr7q21.3	0			x					
1108	218618_s_at	FNDC3B	chr3q26.31	0	x	x						x
1109	202912_at	ADM	chr11p15.4	0	x							
1110	204932_at	TNFRSF11B	chr8q24	0	x							x
1111	218730_s_at	OGN	chr9q22	0	x							x
1112	202181_at	KIAA0247	chr14q24.1	0	x							x
1113	218615_s_at	TMEM39A	chr3q13.33	0	x							
1114	202558_s_at	STCH	chr21q11.1|21q11	0	x							x
1115	207172_s_at	CDH11	chr16q22.1	0	x							
1116	242403_at	UBE2D3	chr4q24	0			x					x
1117	225611_at	---	---	0			x	x	x			x
1118	1568957_x_at	SRGAP2	chr1q32.1	0						x		
1119	227080_at	ZNF697	chr1p12	0			x	x	x			
1120	225030_at	FAM44B	chr5q35.2	0			x					
1121	215305_at	PDGFRA /// TI	chr4q11-q13 /// chr1:	0	x							
1122	205560_at	PCSK5	chr9q21.3	0	x							
1123	226913_s_at	SOX8	chr16p13.3	0			x					
1124	210540_s_at	B4GALT4	chr3q13.3	0	x							
1125	207191_s_at	ISLR	chr15q23-q24	0	x	x						
1126	203868_s_at	VCAM1	chr1p32-p31	0	x	x						x
1127	235077_at	MEG3	chr14q32	0			x					x
1128	205168_at	DDR2	chr1q12-q23	0	x							
1129	239808_at	PITPNC1	chr17q24.2	0			x	x	x			x
1130	209356_x_at	EFEMP2	chr11q13	0	x							x
1131	204512_at	HIVEP1	chr6p24-p22.3	0	x							
1132	206667_s_at	SCAMP1	chr5q13.3-q14.1	0	x							x
1133	1565703_at	SMAD4	chr18q21.1	0						x	x	x
1134	213459_at	RPL37A	chr2q35	0	x							
1135	232180_at	---	---	0			x					x
1136	217403_s_at	ZNF227	---	0	x							
1137	221617_at	TAF9B	chrXq13.1-q21.1	0	x							
1138	201294_s_at	WSB1	chr17q11.1	0	x							x
1139	213446_s_at	IQGAP1	chr15q26.1	0	x							
1140	218056_at	BFAR	chr16p13.12	0	x							x

1141	214390_s_at	BCAT1	chr12pter-q12	0	x						
1142	229435_at	GLIS3	chr9p24.2	0			x	x			
1143	238070_at	CHD1L	chr1q12	0			x				
1144	212396_s_at	KIAA0090	chr1p36.13	0	x						
1145	238951_at	PRDX6	chr1q25.1	0			x				
1146	215084_s_at	LRRC42	chr1p33-p32.1	0	x						
1147	230499_at	BIRC3	chr11q22	0			x				
1148	211029_x_at	FGF18	chr5q34	0	x						
1149	211623_s_at	FBL	chr19q13.1	0	x						
1150	228128_x_at	PAPPA	chr9q33.2	0			x	x			x
1151	225076_s_at	ZNFX1	chr20q13.13	0			x				
1152	207543_s_at	P4HA1	chr10q21.3-q23.1	0	x						x
1153	238909_at	S100A10	chr1q21	0			x				
1154	212481_s_at	TPM4	chr19p13.1	0	x						x
1155	202402_s_at	CARS	chr11p15.5	0	x						x
1156	1552626_a_at	TMEM163	chr2q21.3	0					x	x	
1157	213134_x_at	BTG3	chr21q21.1-q21.2	0	x						x
1158	212940_at	COL6A1	chr21q22.3	0	x						x
1159	233376_at	C6orf160	chr6q14.3	0			x				
1160	1565149_at	DYNC2H1	chr11q21-q22.1	0					x		
1161	231001_at	LOC387758	chr11p14.2	0			x				x
1162	232865_at	AFF4	chr5q31	0			x				
1163	216504_s_at	SLC39A8	chr4q22-q24	0	x						x
1164	1559490_at	LRCH3	chr3q29	0					x		
1165	238908_at	CALU	chr7q32	0			x				x
1166	228253_at	LOXL3	chr2p13	0			x				
1167	232523_at	MEGF10	chr5q33	0			x				
1168	219257_s_at	SPHK1	chr17q25.2	0	x						
1169	218451_at	CDCP1	chr3p21.31	0	x						x
1170	241843_at	SNORA28	chr14q32.32	0			x				
1171	227295_at	IKIP	chr12q23.1	0			x				x
1172	236462_at	YEATS2	chr3q27.1	0			x				
1173	223216_x_at	ZNF395 /// FB	chr8p21.1	0			x				
1174	216109_at	THRAP2	chr12q24.21	0	x						
1175	244312_at	AFTIPHILIN	chr2p14	0			x				
1176	203479_s_at	OTUD4	chr4q31.21	0	x						x
1177	201631_s_at	IER3	chr6p21.3	0	x	x					x
1178	55093_at	CSGlcA-T	chr7q36.1	0	x						
1179	213332_at	PAPPA2	chr1q23-q25	0	x						
1180	241705_at	ABCA5	chr17q24.3	0			x				
1181	244646_at	MSI2	chr17q22	0			x				
1182	1553105_s_at	DSG2	chr18q12.1	0					x	x	x
1183	210389_x_at	TUBD1	chr17q23.1	0	x						
1184	235111_at	---	---	0			x				x
1185	213137_s_at	PTPN2	chr18p11.3-p11.2	0	x						x
1186	1556067_a_at	JMJD3	chr17p13.1	0					x		x
1187	232591_s_at	TMEM30A	chr6q14.1	0			x				x
1188	217297_s_at	MYO9B	chr19p13.1	0	x						
1189	203315_at	NCK2	chr2q12	0	x						
1190	221964_at	TULP3	chr12p13.3	0	x						

1191	203819_s_at	IGF2BP3	chr7p11	0	x					
1192	212268_at	SERPINB1	chr6p25	0	x	x				
1193	227077_at	ZNF286	chr17p11.2	0			x			
1194	209067_s_at	HNRPDL	chr4q13-q21	0	x					
1195	219869_s_at	SLC39A8	chr4q22-q24	0	x					x
1196	215034_s_at	TM4SF1	chr3q21-q25	0	x	x				x
1197	201262_s_at	BGN	chrXq28	0	x					x
1198	232476_at	DDEF2	chr2p25|2p24	0			x			
1199	220266_s_at	KLF4	chr9q31	0	x					x
1200	1569362_at	ALCAM	chr3q13.1	0					x	
1201	219631_at	LRP12	chr8q22.2-q23.1	0	x					
1202	204156_at	KIAA0999	chr11q23.3	0	x					x
1203	201865_x_at	NR3C1	chr5q31.3	0	x					x
1204	225742_at	MDM4	chr1q32	0			x			
1205	206904_at	MATN1	chr1p35	0	x					x
1206	202134_s_at	WWTR1	chr3q23-q24	0	x					
1207	243134_at	LOC440309	chr15q26.1	0			x			
1208	204725_s_at	NCK1	chr3q21	0	x					x
1209	201792_at	AEBP1	chr7p13	0	x					
1210	227850_x_at	CDC42EP5	chr19q13.42	0			x	x		
1211	203650_at	PROCR	chr20q11.2	0	x					
1212	222651_s_at	TRPS1	chr8q24.12	0			x	x		x
1213	229016_s_at	TRERF1	chr6p21.1-p12.1	0			x			
1214	231989_s_at	LOC23117 /// I	chr16p12.1	0			x			
1215	202278_s_at	SPTLC1	chr9q22.2	0	x					
1216	238644_at	MYSM1	chr1p32.1	0			x			
1217	206220_s_at	RASA3	chr13q34	0	x					
1218	208536_s_at	BCL2L11	chr2q13	0	x					
1219	232776_at	PDZRN3	chr3p13	0			x			
1220	207714_s_at	SERPINH1	chr11q13.5	0	x	x				x
1221	239404_at	TOP1	chr20q12-q13.1	0			x			
1222	225698_at	C5orf26	chr5q21-q22	0			x			
1223	242366_at	KIAA0701	chr12q23.1	0			x			x
1224	1554340_a_at	C1orf187	chr1p36.22	0					x	
1225	223217_s_at	NFKBIZ	chr3p12-q12	0			x	x		x
1226	233931_at	ELF1	chr13q13	0			x			
1227	239329_at	---	---	0			x			x
1228	201543_s_at	SAR1A	chr10q22.1	0	x					
1229	221765_at	---	---	0	x					x
1230	222574_s_at	DHX40	chr17q23.1	0			x			
1231	212048_s_at	YARS	chr1p35.1	0	x					x
1232	208698_s_at	NONO	chrXq13.1	0	x					x
1233	225547_at	SNHG6	chr8q13	0			x			
1234	201124_at	ITGB5	chr3q21.2	0	x					x
1235	241820_at	RIF1	chr2q23.3	0			x			x
1236	216222_s_at	MYO10	chr5p15.1-p14.3	0	x					x
1237	238017_at	RDHE2	chr8q12.1	0			x			
1238	227125_at	IL10RB	chr21q22.1-q22.2|21	0			x			x
1239	223304_at	SLC37A3	chr7q34	0			x			x
1240	1560443_at	SMCHD1	chr18p11.32	0					x	

1241	221989_at	RPL10 /// LOC	chrXq28 /// chr19p13	0	x								
1242	224601_at	---	---	0				x					x
1243	218438_s_at	MED28	chr4p16	0	x								
1244	243108_at	---	---	0				x					x
1245	227140_at	---	---	0				x	x				x
1246	221447_s_at	GLT8D2	chr12q	0	x	x							x
1247	210904_s_at	IL13RA1	chrXq24	0	x								x
1248	202662_s_at	ITPR2	chr12p11	0	x								
1249	203108_at	GPRC5A	chr12p13-p12.3	0	x	x							x
1250	207336_at	SOX5	chr12p12.1	0	x	x	x						x
1251	220246_at	CAMK1D	chr10p13	0	x								x
1252	204163_at	EMILIN1	chr2p23.3-p23.2	0	x								
1253	1558365_at	PGK1	chrXq13	0						x			
1254	214172_x_at	RYK	chr3q22	0	x								x
1255	201623_s_at	DARS	chr2q21.3	0	x								x
1256	217057_s_at	GNAS	chr20q13.3	0	x								x
1257	243010_at	MSI2	chr17q22	0				x					
1258	209393_s_at	EIF4E2	chr2q37.1	0	x								
1259	223333_s_at	ANGPTL4	chr19p13.3	0				x					
1260	214004_s_at	VGLL4	chr3p25.2	0	x								
1261	209773_s_at	RRM2	chr2p25-p24	0	x								
1262	244112_x_at	FLJ10159	chr6q21	0				x	x				
1263	218708_at	NXT1	chr20p12-p11.2	0	x								
1264	1558710_at	ARIH1	chr15q24	0						x	x	x	
1265	224763_at	RPL37	chr5p13	0				x					x
1266	241387_at	PTK2	chr8q24-qter	0				x					
1267	205131_x_at	CLEC11A	chr19q13.3	0	x								
1268	211700_s_at	TRO	chrXp11.22-p11.21	0	x								
1269	221584_s_at	KCNMA1	chr10q22.3	0	x								
1270	1558199_at	FN1	chr2q34	0						x	x		x
1271	217952_x_at	PHF3	chr6q12	0	x								
1272	220576_at	PGAP1	chr2q33.1	0	x								
1273	201179_s_at	GNAI3	chr1p13	0	x								
1274	210995_s_at	TRIM23	chr5q12.3	0	x								
1275	212470_at	SPAG9	chr17q21.33	0	x								
1276	228482_at	CDRT4	chr17p12	0				x					
1277	218136_s_at	SLC25A37	chr8p21.2	0	x								
1278	211947_s_at	BAT2D1	chr1q23.3	0	x								x
1279	244455_at	KCNT2	chr1q31.3	0				x	x				
1280	213668_s_at	SOX4	chr6p22.3	0	x								
1281	215191_at	---	---	0	x								x
1282	201897_s_at	CKS1B	chr1q21.2	0	x								
1283	222562_s_at	TNKS2	chr10q23.3	0				x					
1284	220066_at	CARD15	chr16q21	0	x								
1285	244860_at	---	---	0				x					x
1286	221123_x_at	ZNF395	chr8p21.1	0	x	x							
1287	213905_x_at	BGN /// TSHZ1	chrXq28 /// chr18q22	0	x	x							
1288	1555167_s_at	PBEF1	chr7q22.2	0						x	x		
1289	232307_at	THRAP2	chr12q24.21	0				x					
1290	214023_x_at	TUBB2B	chr6p25	0	x	x							

1291	229908_s_at	---	---	0			x				x
1292	210260_s_at	TNFAIP8	chr5q23.1	0	x	x					x
1293	202014_at	PPP1R15A	chr19q13.2	0	x	x					x
1294	230068_s_at	PEG3	chr19q13.4	0			x				x
1295	202436_s_at	CYP1B1	chr2p21	0	x	x					x
1296	204472_at	GEM	chr8q13-q21	0	x	x					x
1297	220937_s_at	ST6GALNAC4	chr9q34	0	x						
1298	206499_s_at	RCC1	chr1p36.1	0	x						
1299	229514_at	C14orf118	chr14q22.1-q24.3	0			x				
1300	1552618_at	STX6	chr1q25.3	0					x		x
1301	58780_s_at	FLJ10357	chr14q11.2	0	x						
1302	235662_at	CCDC104	chr2p16.1	0			x				
1303	217523_at	CD44	chr11p13	0	x						x
1304	209281_s_at	ATP2B1	chr12q21.3	0	x						x
1305	232528_at	UBE2E3	chr2q32.1	0			x	x			
1306	226769_at	LOC387758	chr11p14.2	0			x				x
1307	228171_s_at	PLEKHG4	chr16q22.1	0			x				
1308	224523_s_at	C3orf26	chr3q12.1	0			x				
1309	226432_at	ETNK1 /// LOC	chr12p12.1	0			x				
1310	238568_s_at	C18orf8	chr18q11.2	0			x				
1311	224807_at	GRAMD1A	chr19q13.11	0			x				
1312	222812_s_at	RHOF	chr12q24.31	0			x				
1313	238623_at	---	---	0			x				x
1314	204022_at	WWP2	chr16q22.1	0	x						
1315	235421_at	MAP3K8	chr10p11.23	0			x				x
1316	225303_at	KIRREL	chr1q21-q25	0			x				
1317	242853_at	---	---	0			x	x			x
1318	202235_at	SLC16A1	chr1p12	0	x						
1319	235745_at	ERN1	chr17q24.2	0			x				
1320	232431_at	NR3C1	chr5q31.3	0			x	x			x
1321	212521_s_at	PDE8A	chr15q25.3	0	x						
1322	1553995_a_at	NT5E	chr6q14-q21	0					x	x	
1323	218310_at	RABGEF1	chr7q11.21	0	x						x
1324	219501_at	RP11-301I17.1	chr13q14.11	0	x						
1325	210692_s_at	SLC43A3	chr11q11	0	x						
1326	204038_s_at	EDG2	chr9q31.3	0	x						
1327	238649_at	PITPNC1	chr17q24.2	0			x				x
1328	222235_s_at	GALNACT-2 //	chr10q11.21 /// chrX	0	x						
1329	238848_at	OTUD4	chr4q31.21	0			x				x
1330	215493_x_at	BTN2A1	chr6p22.1	0	x						
1331	212732_at	MEG3	chr14q32	0	x						x
1332	206825_at	OXTR	chr3p25	0	x						
1333	244803_at	DAP3	chr1q21-q22	0			x				x
1334	1555233_at	RHOJ	chr14q23.2	0					x	x	
1335	236168_at	ACSL3	chr2q34-q35	0			x				x
1336	236640_at	LOC399959	chr11q24.1	0			x				x
1337	213034_at	KIAA0999	chr11q23.3	0	x						x
1338	210041_s_at	PGM3	chr6q14.1-q15	0	x						
1339	226348_at	---	---	0			x				x
1340	237640_at	C14orf138	chr14q22.1	0			x				

1341	1566869_at	LOC645380	chr14q22.1	0							x		
1342	200998_s_at	CKAP4	chr12q23.3	0	x								x
1343	1555854_at	---	---	0							x	x	x
1344	204281_at	TEAD4	chr12p13.2-p13.3	0	x								
1345	213700_s_at	PKM2	chr15q22	0	x								x
1346	212642_s_at	HIVEP2	chr6q23-q24	0	x								
1347	226825_s_at	TMEM165	chr4q12	0				x					
1348	212208_at	THRAP2	chr12q24.21	0	x								
1349	204669_s_at	RNF24	chr20p13-p12.1	0	x	x	x						
1350	208097_s_at	TXNDC	chr14q22.1	0	x								
1351	228952_at	---	---	0				x					x
1352	215012_at	ZNF451	chr6p12.1	0	x								
1353	239096_at	C14orf32	chr14q22.3	0				x					
1354	208453_s_at	XPNPEP1	chr10q25.3	0	x								
1355	202200_s_at	SRPK1	chr6p21.3-p21.2	0	x								
1356	200779_at	ATF4	chr22q13.1	0	x								x
1357	1558170_at	---	---	0							x	x	x
1358	222646_s_at	ERO1L	chr14q22.1	0				x	x	x			
1359	232902_s_at	RARSL	chr6q16.1	0				x					
1360	225857_s_at	LOC388796	chr20q11.23	0				x					x
1361	211599_x_at	MET	chr7q31	0	x								x
1362	238311_at	KIAA0776	chr6q16.1	0				x					
1363	222443_s_at	RBM8A	chr1q12	0				x					x
1364	1570507_at	CTNNB1	chr3p21	0							x		
1365	226897_s_at	ZC3H7A	chr16p13-p12	0				x					x
1366	206359_at	SOCS3	chr17q25.3	0	x								x
1367	202765_s_at	FBN1	chr15q21.1	0	x								x
1368	231973_s_at	ANAPC1	chr2q12.1	0				x					
1369	1569022_a_at	PIK3C2A	chr11p15.5-p14	0							x		x
1370	202072_at	HNRPL	chr19q13.2	0	x								
1371	224741_x_at	GAS5	chr1q25.1	0				x					x
1372	218717_s_at	LEPREL1	chr3q28	0	x	x							x
1373	214280_x_at	HNRPA1	chr12q13.1	0	x								x
1374	218362_s_at	KIAA1008	chr13q22.1	0	x								x
1375	209883_at	GLT25D2	chr1q25	0	x								
1376	1554780_a_at	PHTF2	chr7q11.23-q21	0							x		x
1377	229947_at	PI15	chr8q21.11	0				x	x				
1378	216624_s_at	MLL	chr11q23	0	x								
1379	202887_s_at	DDIT4	chr10pter-q26.12	0	x								x
1380	219978_s_at	NUSAP1	chr15q15.1	0	x								
1381	212290_at	SLC7A1	chr13q12-q14	0	x								
1382	39549_at	NPAS2	chr2q11.2	0	x								
1383	210302_s_at	MAB21L2	chr4q31	0	x	x							
1384	222807_at	C11orf30	chr11q13.5	0				x					
1385	1558290_a_at	PVT1 /// LOC4	chr8q24 /// chr8q24.2	0							x		
1386	226050_at	TMCO3	chr13q34	0				x					
1387	214096_s_at	SHMT2	chr12q12-q14	0	x								x
1388	216248_s_at	NR4A2	chr2q22-q23	0	x								
1389	222644_s_at	GLT25D1	chr19p13.11	0				x					
1390	202368_s_at	TRAM2	chr6p21.1-p12	0	x								x

1391	209156_s_at	COL6A2	chr21q22.3	0	x	x						x
1392	205027_s_at	MAP3K8	chr10p11.23	0	x							x
1393	224339_s_at	ANGPTL1	chr1q25.2	0				x				
1394	222693_at	FNDC3B	chr3q26.31	0				x	x	x		x
1395	225230_at	TMEM77	chr1p13.3	0				x				
1396	201668_x_at	MARCKS	chr6q22.2	0	x							x
1397	205321_at	EIF2S3	chrXp22.2-p22.1	0	x							
1398	210095_s_at	IGFBP3	chr7p13-p12	0	x	x						x
1399	209267_s_at	SLC39A8	chr4q22-q24	0	x							x
1400	219017_at	ETNK1	chr12p12.1	0	x	x	x					
1401	214212_x_at	PLEKHC1	chr14q22.2	0	x							x
1402	37028_at	PPP1R15A	chr19q13.2	0	x							x
1403	214913_at	ADAMTS3	chr4q13.3	0	x							
1404	203304_at	BAMBI	chr10p12.3-p11.2	0	x							
1405	214967_at	SAMD4A	chr14q22.2	0	x							
1406	209885_at	RHOD	chr11q14.3	0	x	x						
1407	208726_s_at	EIF2S2	chr20pter-q12	0	x							
1408	205120_s_at	SGCB	chr4q12	0	x							
1409	233186_s_at	BANP	chr16q24.2-q24.3	0				x				
1410	240903_at	L3MBTL4	chr18p11.31	0				x				
1411	211571_s_at	CSPG2	chr5q14.3	0	x							x
1412	223650_s_at	NRBF2	chr10q21.2	0				x				
1413	203149_at	PVRL2	chr19q13.2	0	x							
1414	205817_at	SIX1	chr14q23.1	0	x							
1415	204011_at	SPRY2	chr13q31.1	0	x							x
1416	208296_x_at	TNFAIP8	chr5q23.1	0	x	x						x
1417	209387_s_at	TM4SF1	chr3q21-q25	0	x							x
1418	63305_at	PKNOX2	---	0	x							
1419	211952_at	RANBP5	chr13q32.2	0	x							
1420	202771_at	FAM38A	chr16q24.3	0	x							
1421	223028_s_at	SNX9	chr6q25.1-q26	0				x				x
1422	213470_s_at	HNRPH1	chr5q35.3	0	x							
1423	1565681_s_at	DIP2C	chr10p15.3	0							x	
1424	213394_at	MAPKBP1	chr15q15.1	0	x							x
1425	238906_s_at	RHOJ	chr14q23.2	0				x				
1426	229999_at	---	---	0				x				x
1427	202466_at	POLS	chr5p15	0	x							
1428	222262_s_at	ETNK1	chr12p12.1	0	x							
1429	1555247_a_at	RAPGEF6	chr5q31.1	0							x	
1430	243183_at	LSAMP	chr3q13.2-q21	0				x				
1431	200924_s_at	SLC3A2	chr11q13	0	x							x
1432	211944_at	BAT2D1	chr1q23.3	0	x							x
1433	213725_x_at	LOC283824	chr16p13.12	0	x	x	x					x
1434	219305_x_at	FBXO2	chr1p36.22	0	x							
1435	244826_at	PITPNB	chr22q12.1	0				x				
1436	208116_s_at	MAN1A1	chr6q22	0	x							x
1437	202644_s_at	TNFAIP3	chr6q23	0	x	x						x
1438	1558877_at	ELF2	chr4q28	0							x	
1439	238824_at	---	---	0				x				x
1440	244804_at	SQSTM1	chr5q35	0				x	x	x		x

1441	211297_s_at	CDK7	chr5q12.1	0	x						
1442	57163_at	ELOVL1	chr1p34.2	0	x						
1443	1554577_a_at	PSMD10	chrXq22.3	0						x	
1444	213791_at	PENK	chr8q23-q24	0	x	x					
1445	210188_at	GABPA /// GAI	chr21q21-q22.1|21q2	0	x						
1446	225481_at	FRMD6	chr14q22.1	0			x	x			x
1447	212012_at	PXDN	chr2p25	0	x	x					x
1448	243857_at	MORF4L2	chrXq22	0			x				x
1449	202516_s_at	DLG1	chr3q29	0	x						x
1450	206170_at	ADRB2	chr5q31-q32	0	x	x					
1451	224841_x_at	GAS5	chr1q25.1	0			x				x
1452	203742_s_at	TDG	chr12q24.1	0	x						
1453	230424_at	C5orf13	chr5q22.1	0			x				x
1454	206472_s_at	TLE3	chr15q22	0	x						
1455	222529_at	SLC25A37	chr8p21.2	0			x	x			
1456	228758_at	BCL6	chr3q27	0			x	x			x
1457	211701_s_at	TRO	chrXp11.22-p11.21	0	x						
1458	218728_s_at	CNIH4	chr1q42.11	0	x						
1459	215512_at	38782	chr5p15.2	0	x						
1460	236251_at	ITGAV	chr2q31-q32	0			x				
1461	238931_at	METT10D	chr17p13.3	0			x				
1462	230503_at	SAMD4A	chr14q22.2	0			x				
1463	203574_at	NFIL3	chr9q22	0	x						x
1464	1554079_at	GALNTL4	chr11p15.3	0						x	
1465	235204_at	COX15	chr10q24	0			x				x
1466	209204_at	LMO4	chr1p22.3	0	x						
1467	209715_at	CBX5	chr12q13.13	0	x						x
1468	1556066_at	JMJD3	chr17p13.1	0						x	x
1469	216392_s_at	SEC23IP	chr10q25-q26	0	x						x
1470	224725_at	MIB1	chr18q11.2	0			x	x			
1471	210872_x_at	GAS7	chr17p13.1	0	x						x
1472	233002_at	KIAA1622	chr14q32.13	0			x	x			
1473	241769_at	ITGAV	chr2q31-q32	0			x				
1474	203737_s_at	PPRC1	chr10q24.32	0	x						
1475	1557512_at	RSN	chr12q24.3	0						x	x
1476	221539_at	EIF4EBP1	chr8p12	0	x						
1477	226697_at	LOC92689	chr4p14	0			x				
1478	211559_s_at	CCNG2	chr4q21.1	0	x						
1479	222728_s_at	JOSD3	chr11q21	0			x	x			
1480	202191_s_at	GAS7	chr17p13.1	0	x						x
1481	219558_at	ATP13A3	chr3q29	0	x						x
1482	205298_s_at	BTN2A2	chr6p22.1	0	x						
1483	208095_s_at	SRP72	chr4q11	0	x						
1484	214974_x_at	CXCL5	chr4q12-q13	0	x						
1485	225406_at	TWSG1	chr18p11.3	0			x	x			x
1486	219118_at	FKBP11	chr12q13.12	0	x						
1487	230998_at	CBX3	chr7p15.2	0			x				x
1488	224827_at	DC-UbP	chr5q35.1	0			x	x			x
1489	237626_at	RB1CC1	chr8p22-q21.13	0			x	x	x		
1490	207629_s_at	ARHGEF2	chr1q21-q22	0	x						

1491	223054_at	DNAJB11	chr3q27.3	0			x				x
1492	213524_s_at	G0S2	chr1q32.2-q41	0	x	x					
1493	217593_at	ZNF447	chr19q13.43	0	x						
1494	226345_at	---	---	0			x				x
1495	209578_s_at	POFUT2	chr21q22.3	0	x						x
1496	226763_at	SESTD1	chr2q31.2	0			x				
1497	226663_at	ANKRD10	chr13q34	0			x				
1498	210470_x_at	NONO	chrXq13.1	0	x						x
1499	219377_at	FAM59A	chr18q12.1	0	x	x					
1500	230836_at	ST8SIA4	chr5q21	0			x	x			
1501	228314_at	---	---	0			x	x	x		x
1502	241701_at	ARHGAP21	chr10p12.1|10p12.3	0			x				
1503	209733_at	LOC286440	chrXq22.3	0	x						
1504	1568815_a_at	DDX50	chr10q21.3	0						x	
1505	1554351_a_at	TIPRL	chr1q23.2	0						x	
1506	227787_s_at	THRAP6	chr8q24.11	0			x				
1507	216088_s_at	PSMA7	chr20q13.33	0	x						x
1508	228245_s_at	HELZ /// OVOS	chr17q24.2 /// chr12p	0			x	x			
1509	225186_at	RAPH1	chr2q33	0			x				x
1510	201471_s_at	SQSTM1	chr5q35	0	x						x
1511	202016_at	MEST	chr7q32	0	x	x					x
1512	235927_at	THRB	chr3p24.2	0			x				
1513	226416_at	THEX1	chr8p23.1	0			x				
1514	218515_at	C21orf66	chr21q21.3	0	x						
1515	234645_at	MAML2	chr11q21	0			x				
1516	225827_at	EIF2C2	chr8q24	0			x				x
1517	223430_at	SNF1LK2	chr11q23.1	0			x				
1518	212143_s_at	IGFBP3	chr7p13-p12	0	x	x					x
1519	229247_at	FLJ37440	chr2q13	0			x				
1520	235484_at	PTAR1	chr9q21.11	0			x				
1521	219179_at	DACT1	chr14q23.1	0	x	x					
1522	213146_at	JMJD3	chr17p13.1	0	x						x
1523	219922_s_at	LTBP3	chr11q12	0	x						x
1524	202611_s_at	CRSP2	chrXp11.4-p11.2	0	x						
1525	207943_x_at	PLAGL1	chr6q24-q25	0	x						x
1526	219514_at	ANGPTL2	chr9q34	0	x	x					x
1527	209566_at	INSIG2	chr2q14.1	0	x						
1528	235234_at	FLJ36874	chr11q12.1	0			x				x
1529	225553_at	---	---	0			x				x
1530	200973_s_at	TSPAN3	chr15q24.3	0	x						
1531	203231_s_at	ATXN1	chr6p23	0	x						x
1532	232134_at	POLS	chr5p15	0			x				
1533	211681_s_at	PDLIM5	chr4q22	0	x						x
1534	200956_s_at	SSRP1	chr11q12	0	x						x
1535	217951_s_at	PHF3	chr6q12	0	x						
1536	211031_s_at	CYLN2	chr7q11.23	0	x						
1537	224721_at	WDR75	chr2q32.2	0			x				
1538	211709_s_at	CLEC11A	chr19q13.3	0	x	x					
1539	207332_s_at	TFRC	chr3q29	0	x						
1540	1559949_at	TRPS1	chr8q24.12	0						x	x

1541	201874_at	MPZL1	chr1q24.2	0	x								x
1542	218028_at	ELOVL1	chr1p34.2	0	x								
1543	209127_s_at	SART3	chr12q24.1	0	x								
1544	225520_at	MTHFD1L	chr6q25.1	0				x					
1545	1555996_s_at	EIF4A2	chr3q28	0							x	x	x
1546	205066_s_at	ENPP1	chr6q22-q23	0	x	x							x
1547	232098_at	DST	chr6p12-p11	0				x					
1548	203944_x_at	BTN2A1	chr6p22.1	0	x								
1549	202568_s_at	MARK3	chr14q32.3	0	x								
1550	202954_at	PAK3 /// UBE2	chrXq22.3-q23 /// chr	0	x								
1551	203921_at	CHST2	chr3q24	0	x								
1552	203720_s_at	ERCC1	chr19q13.2-q13.3	0	x								x
1553	219045_at	RHOF	chr12q24.31	0	x								
1554	224834_at	DC-UbP	chr5q35.1	0				x					x
1555	236685_at	---	---	0				x	x	x			x
1556	205891_at	ADORA2B	chr17p12-p11.2	0	x								
1557	207782_s_at	PSEN1	chr14q24.3	0	x								
1558	223185_s_at	BHLHB3	chr12p11.23-p12.1	0				x					
1559	218238_at	GTPBP4	chr10p15-p14	0	x								
1560	32094_at	CHST3	chr10q22.1	0	x	x	x						
1561	213113_s_at	SLC43A3	chr11q11	0	x								
1562	216278_at	KIAA0256	chr15q21.1	0	x								
1563	221920_s_at	SLC25A37	chr8p21.2	0	x								
1564	210513_s_at	VEGF	chr6p12	0	x								x
1565	232095_at	SRGAP2	chr1q32.1	0				x					
1566	214737_x_at	HNRPC	chr14q11.2	0	x								x
1567	204157_s_at	KIAA0999	chr11q23.3	0	x								x
1568	222916_s_at	HDLBP	chr2q37	0				x					
1569	1555240_s_at	GNG12	chr1p31.3	0							x		x
1570	224859_at	CD276	chr15q23-q24	0				x					
1571	226582_at	LOC400043	chr12q13.13	0				x					
1572	203354_s_at	PSD3	chr8pter-p23.3	0	x								x
1573	220197_at	ATP6V0A4	chr7q33-q34	0	x								
1574	221050_s_at	GTPBP2	chr6p21-p12	0	x								x
1575	232746_at	CMKOR1	chr2q37.3	0				x					
1576	215195_at	PRKCA	chr17q22-q23.2	0	x								x
1577	239164_at	---	---	0				x					x
1578	232141_at	U2AF1	chr21q22.3	0				x					x
1579	220890_s_at	DDX47	chr12p13.1	0	x								
1580	203124_s_at	SLC11A2	chr12q13	0	x								
1581	209027_s_at	ABI1	chr10p11.2	0	x								x
1582	201654_s_at	HSPG2	chr1p36.1-p34	0	x								
1583	211527_x_at	VEGF	chr6p12	0	x	x							x
1584	217208_s_at	DLG1 /// TNFF	chr3q29 /// chr8q24	0	x								
1585	221530_s_at	BHLHB3	chr12p11.23-p12.1	0	x								
1586	239704_at	IBRDC2	chr6p22.3	0				x					
1587	241879_at	---	---	0				x					x
1588	1560342_at	ZFAND3	chr6pter-p22.3	0							x		
1589	201108_s_at	THBS1	chr15q15	0	x	x							x
1590	220610_s_at	LRRFIP2	chr3p22.2	0	x								x

1591	201627_s_at	INSIG1	chr7q36	0	x							x
1592	201995_at	EXT1	chr8q24.11-q24.13	0	x							
1593	212468_at	SPAG9	chr17q21.33	0	x							
1594	232304_at	PELI1	chr2p13.3	0			x	x				x
1595	205010_at	GNL3L	chrXp11.22	0	x							
1596	227226_at	C6orf117	chr6q14.3	0			x					x
1597	224933_s_at	JMJD1C	chr10q21.2	0			x					x
1598	222763_s_at	WDR33	chr2q14.3	0			x					x
1599	210284_s_at	MAP3K7IP2	chr6q25.1-q25.3	0	x							x
1600	1565804_at	ATXN1	chr6p23	0					x			x
1601	207011_s_at	PTK7	chr6p21.1-p12.2	0	x							
1602	228325_at	KIAA0146	chr8q11.21	0			x					
1603	213804_at	INPP5B	chr1p34	0	x							
1604	225275_at	EDIL3	chr5q14	0			x	x				x
1605	231822_at	CTTNBP2NL	chr1p13.2	0			x					
1606	1555841_at	---	---	0					x	x	x	x
1607	222418_s_at	TMEM43	chr3p25.1	0			x					x
1608	214104_at	GPR161	chr1q24.2	0	x							
1609	205443_at	SNAPC1	chr14q22	0	x							x
1610	212104_s_at	RBM9	chr22q13.1	0	x							x
1611	242230_at	ATXN1	chr6p23	0			x					x
1612	219416_at	SCARA3	chr8p21	0	x							
1613	242201_at	PMS2L1	chr7q22.1	0			x					
1614	1553133_at	C9orf72	chr9p21.2	0					x			
1615	237554_at	ACVR1	chr2q23-q24	0			x					x
1616	202301_s_at	FLJ11021	chr12q24.31	0	x							
1617	229090_at	LOC220930	chr10p11.23	0			x					x
1618	235526_at	SOX6	chr11p15.3	0			x					
1619	208152_s_at	DDX21	chr10q21	0	x							
1620	201571_s_at	DCTD	chr4q35.1	0	x							x
1621	219312_s_at	ZBTB10	chr8q13-q21.1	0	x							
1622	1555460_a_at	SLC39A6	chr18q12.2	0					x	x		
1623	203639_s_at	FGFR2	chr10q26	0	x							x
1624	229272_at	FNBP4	chr11p11.2	0			x					x
1625	210701_at	CFDP1	chr16q22.2-q22.3	0	x							x
1626	206377_at	FOXF2	chr6p25.3	0	x							
1627	238436_s_at	LOC390980	chr19q13.43	0			x					
1628	209699_x_at	AKR1C2	chr10p15-p14	0	x	x						
1629	202147_s_at	IFRD1	chr7q22-q31	0	x							x
1630	203992_s_at	UTX	chrXp11.2	0	x							
1631	241457_at	FBXL7	chr5p15.1	0			x	x				x
1632	239794_at	IARS	chr9q21	0			x					x
1633	206686_at	PDK1	chr2q31.1	0	x							
1634	201478_s_at	DKC1	chrXq28	0	x							
1635	205573_s_at	SNX7	chr1p21.3	0	x							x
1636	236836_at	TBCA	chr5q14.1	0			x					
1637	215558_at	UBR2	chr6p21.1	0	x							
1638	205603_s_at	DIAPH2	chrXq22	0	x							
1639	229490_s_at	---	---	0			x					x
1640	233899_x_at	ZBTB10	chr8q13-q21.1	0			x	x				

1641	201389_at	ITGA5	chr12q11-q13	0	x						
1642	229218_at	COL1A2	chr7q22.1	0				x			x
1643	203816_at	DGUOK	chr2p13	0	x						
1644	221699_s_at	DDX50	chr10q21.3	0	x						
1645	204688_at	SGCE	chr7q21-q22	0	x						x
1646	201386_s_at	DHX15	chr4p15.3	0	x						
1647	222406_s_at	LOC653105	---	0				x			
1648	225416_at	RNF12	chrXq13-q21	0				x			
1649	229050_s_at	SNHG7	chr9q34.3	0				x			
1650	223383_at	ZNRF1	chr16q23.1	0				x			x
1651	236635_at	ZNF667	chr19q13.43	0				x			
1652	231861_at	LRP10	chr14q11.2	0				x			
1653	217795_s_at	TMEM43	chr3p25.1	0	x						x
1654	227210_at	---	---	0				x			x
1655	239771_at	CAND1	chr12q14	0				x			x
1656	200652_at	SSR2	chr1q21-q23	0	x						x
1657	204132_s_at	FOXO3A	chr6q21	0	x						x
1658	225805_at	HNRPU	chr1q44	0				x			x
1659	205446_s_at	ATF2	chr2q32	0	x						
1660	207704_s_at	GAS7	chr17p13.1	0	x						x
1661	204679_at	KCNK1	chr1q42-q43	0	x						
1662	212769_at	TLE3	chr15q22	0	x						
1663	239571_at	MEF2A	chr15q26	0				x			
1664	228365_at	CPNE8	chr12q12	0				x			
1665	213428_s_at	COL6A1	chr21q22.3	0	x						x
1666	201751_at	JOSD1	chr22q13.1	0	x						x
1667	1552486_s_at	LACTB	chr15q22.1	0						x	
1668	200872_at	S100A10	chr1q21	0	x						
1669	229070_at	C6orf105	chr6p24.1	0				x	x		
1670	223382_s_at	ZNRF1	chr16q23.1	0				x			x
1671	213554_s_at	CDV3	chr3q22.1	0	x						
1672	203060_s_at	PAPSS2	chr10q23-q24	0	x	x					
1673	226347_at	---	---	0				x			x
1674	216449_x_at	HSP90B1	chr12q24.2-q24.3	0	x						
1675	220673_s_at	KIAA1622	chr14q32.13	0	x						
1676	203851_at	IGFBP6	chr12q13	0	x						
1677	211612_s_at	IL13RA1	chrXq24	0	x						x
1678	222464_s_at	C10orf119	chr10q26.11	0				x			x
1679	212008_at	UBXD2	chr2q21.3	0	x						x
1680	204435_at	NUPL1	chr13q12.13	0	x	x	x				x
1681	225140_at	KLF3	chr4p14	0				x			x
1682	225381_at	LOC399959	chr11q24.1	0				x	x		x
1683	225868_at	TRIM47	---	0				x			x
1684	236620_at	RIF1	chr2q23.3	0				x			x
1685	205841_at	JAK2	chr9p24	0	x						
1686	225442_at	---	---	0				x			x
1687	214336_s_at	COPA	chr1q23-q25	0	x						
1688	1569788_at	ST8SIA1	chr12p12.1-p11.2	0						x	
1689	217850_at	GNL3	chr3p21.1	0	x						x
1690	208930_s_at	ILF3	chr19p13.2	0	x						x

1691	224719_s_at	C12orf57	chr12p13.31	0			x					
1692	227058_at	C13orf33	chr13q12.3	0			x					
1693	1557505_a_at	SRP54	chr14q13.2	0						x		
1694	243591_at	LAMC1	chr1q31	0			x					
1695	242712_x_at	LOC653086 ///	chr2q13 /// chr2q12.	0			x					
1696	212350_at	TBC1D1	chr4p14	0	x							
1697	216594_x_at	AKR1C1	chr10p15-p14	0	x	x						
1698	207173_x_at	CDH11	chr16q22.1	0	x	x						
1699	239175_at	AFTIPHILIN	chr2p14	0			x					
1700	224013_s_at	SOX7	---	0			x					
1701	202382_s_at	GNPDA1	chr5q21	0	x							x
1702	203313_s_at	TGIF	chr18p11.3	0	x							x
1703	238913_at	CPSF6	chr12q15	0			x					x
1704	214375_at	PPFIBP1	chr12p11.23-p11.22	0	x							x
1705	231199_at	MAK3	chr3q13.2	0			x					x
1706	213004_at	ANGPTL2	chr9q34	0	x							x
1707	219375_at	CEPT1	chr1p13.3	0	x							
1708	242374_at	HIAT1	chr1p21.2	0			x					
1709	236439_at	BCL6	chr3q27	0			x					x
1710	1555801_s_at	ZNF533	chr2q31.2-q31.3	0						x	x	
1711	203687_at	CX3CL1	chr16q13	0	x							
1712	215009_s_at	SEC31L1	chr4q21.22	0	x							x
1713	222792_s_at	CCDC59	chr12q21.31	0			x					
1714	226337_at	SCYL1BP1	chr1q24.2	0			x	x	x			
1715	235167_at	LOC23117	chr16p12.1	0			x					
1716	222759_at	SUV420H1	chr11q13.2	0			x					x
1717	1554696_s_at	TYMS	chr18p11.32	0						x	x	
1718	226320_at	THOC4	chr17q25.3	0			x					
1719	219825_at	CYP26B1	chr2p13.3	0	x	x						
1720	200784_s_at	LRP1	chr12q13-q14	0	x							
1721	212171_x_at	VEGF	chr6p12	0	x							x
1722	218750_at	JOSD3	chr11q21	0	x							
1723	218096_at	AGPAT5	chr8p23.1	0	x							x
1724	238949_at	---	---	0			x					x
1725	223158_s_at	NEK6	chr9q33.3-q34.11	0			x					
1726	229228_at	CREB5	chr7p15.1	0			x	x				
1727	239576_at	MTUS1	chr8p22	0			x					x
1728	213461_at	NUDT21	chr16q13	0	x							
1729	225032_at	FNDC3B	chr3q26.31	0			x	x				x
1730	228115_at	---	---	0			x					x
1731	242920_at	KIAA0999	chr11q23.3	0			x	x	x			x
1732	225150_s_at	RTKN	chr2p13.1	0			x					x
1733	219569_s_at	TMEM22	chr3q22.3	0	x							x
1734	211974_x_at	RBPSUH	chr4p15.2	0	x							x
1735	221269_s_at	SH3BGRL3	chr1p35-p34.3	0	x							x
1736	209055_s_at	CDC5L	chr6p21	0	x							x
1737	221220_s_at	SCYL2	chr12q23.1	0	x							
1738	223623_at	C2orf40	chr2q12.2	0			x	x				x
1739	205429_s_at	MPP6	chr7p15	0	x							
1740	208777_s_at	PSMD11	chr17q11.2	0	x							x

1741	219935_at	ADAMTS5	chr21q21.3	0	x							
1742	1552610_a_at	JAK1	chr1p32.3-p31.3	0						x	x	
1743	206113_s_at	RAB5A	chr3p24-p22	0	x							x
1744	215253_s_at	DSCR1	chr21q22.1-q22.2|21	0	x							x
1745	206022_at	NDP	chrXp11.4	0	x							
1746	224883_at	PLDN	chr15q21.1	0			x					x
1747	201069_at	MMP2	chr16q13-q21	0	x	x						x
1748	202528_at	GALE	chr1p36-p35	0	x							x
1749	226121_at	DHRS13	chr17q11.2	0			x					
1750	208790_s_at	PTRF	chr17q21.31	0	x							x
1751	200898_s_at	MGEA5	chr10q24.1-q24.3	0	x							
1752	202076_at	BIRC2	chr11q22	0	x							x
1753	243046_at	UBE2D3	chr4q24	0			x					x
1754	223395_at	ABI3BP	chr3q12	0			x	x				
1755	224646_x_at	H19	chr11p15.5	0			x	x				x
1756	203010_at	STAT5A	chr17q11.2	0	x							x
1757	230097_at	GART	chr21q22.1|21q22.11	0			x					x
1758	203293_s_at	LMAN1	chr18q21.3-q22	0	x							
1759	236131_at	---	---	0			x					x
1760	204851_s_at	DCX	chrXq22.3-q23	0	x							
1761	1552978_a_at	SCAMP1	chr5q13.3-q14.1	0						x		x
1762	232323_s_at	TTC17	chr11p12-p11.2	0			x					
1763	218412_s_at	GTF2IRD1	chr7q11.23	0	x							x
1764	200744_s_at	GNB1	chr1p36.33	0	x							x
1765	221691_x_at	NPM1	chr5q35	0	x							x
1766	229404_at	TWIST2	chr2q37.3	0			x					
1767	202450_s_at	CTSK	chr1q21	0	x							
1768	205184_at	GNG4	chr1q42.3	0	x							
1769	1554948_at	---	---	0						x		x
1770	212085_at	SLC25A6	chrXp22.32 and Yp1	0	x							x
1771	214820_at	BRWD1	chr21q22.2	0	x							
1772	212018_s_at	RSL1D1	chr16p13.13	0	x							
1773	207769_s_at	PQBP1	chrXp11.23	0	x							x
1774	231202_at	ALDH1L2	chr12q23.3	0			x					
1775	217492_s_at	PTEN	chr10q23.3	0	x							
1776	236975_at	USP12	chr13q12.13	0			x	x	x			
1777	225955_at	MED25 /// ME	chr19q13.3 /// chr17c	0			x					
1778	241734_at	SRFBP1	chr5q23.1	0			x					x
1779	217823_s_at	UBE2J1	chr6q15	0	x							x
1780	227041_at	---	---	0			x					x
1781	228483_s_at	TAF9B	chrXq13.1-q21.1	0			x					
1782	225299_at	MYO5B	chr18q21	0			x	x				
1783	224710_at	RAB34	chr17q11.2	0			x					
1784	204151_x_at	AKR1C1	chr10p15-p14	0	x							
1785	219602_s_at	FAM38B	chr18p11.22	0	x							
1786	205542_at	STEAP1	chr7q21	0	x							
1787	214527_s_at	PQBP1	chrXp11.23	0	x							x
1788	242691_at	---	---	0			x					x
1789	203294_s_at	LMAN1	chr18q21.3-q22	0	x							
1790	227249_at	NDE1	chr16p13.11	0			x					x

1791	1557360_at	LRPPRC	chr2p21	0					x	x	
1792	212753_at	PCGF3	chr4p16.3	0	x						
1793	212190_at	SERPINE2	chr2q33-q35	0	x						x
1794	233286_at	PDZRN3	chr3p13	0			x				
1795	216115_at	NF1	chr17q11.2	0	x						
1796	215203_at	GOLGA4	chr3p22-p21.3	0	x	x					
1797	221580_s_at	JOSD3	chr11q21	0	x						x
1798	224311_s_at	CAB39	chr2q37.1	0			x				
1799	235122_at	---	---	0			x				x
1800	238299_at	LASS5	chr12q13.13	0			x				
1801	226492_at	SEMA6D	chr15q21.1	0			x	x			
1802	203438_at	STC2	chr5q35.2	0	x						x
1803	212840_at	UBXD7	chr3q29	0	x						
1804	64432_at	C12orf47	chr12q24.12	0	x						
1805	215204_at	SENP6	chr6q13-q14.3	0	x						x
1806	234033_at	RAPGEF2	chr4q32.1	0			x				
1807	225339_at	---	---	0			x				x
1808	204103_at	CCL4	chr17q12	0	x						
1809	227828_s_at	TMEM166	chr2p12	0			x	x			x
1810	203214_x_at	CDC2	chr10q21.1	0	x						x
1811	242394_at	CNKSR3	chr6q25.2	0			x				x
1812	240038_at	ELL2	chr5q15	0			x				x
1813	228425_at	LOC654433	---	0			x				
1814	216274_s_at	SEC11L1	chr15q25.3	0	x						
1815	213594_x_at	FUSIP1 /// LO(	chr1p36.11	0	x						
1816	218613_at	LOC653754	chr8p22	0	x	x					
1817	226930_at	FNDC1	chr6q25	0			x	x			
1818	225562_at	RASA3	chr13q34	0			x				
1819	220755_s_at	C6orf48	chr6p21.3	0	x						
1820	227501_at	WSB1	chr17q11.1	0			x				x
1821	205842_s_at	JAK2	chr9p24	0	x						
1822	230183_at	---	---	0			x				x
1823	228347_at	SIX1	chr14q23.1	0			x				
1824	209471_s_at	FNTA	chr8p22-q11	0	x						
1825	204712_at	WIF1	chr12q14.3	0	x						
1826	232033_at	USP37	chr2q35	0			x				
1827	221806_s_at	SETD5	chr3p25.3	0	x						x
1828	202404_s_at	COL1A2	chr7q22.1	0	x	x					x
1829	242725_at	KDELR2	chr7p22.1	0			x				x
1830	202089_s_at	SLC39A6	chr18q12.2	0	x						
1831	203137_at	WTAP	chr6q25-q27	0	x						
1832	234650_at	LGR4	chr11p14-p13	0			x				x
1833	212445_s_at	NEDD4L	chr18q21	0	x						
1834	235680_at	STAT3	chr17q21.31	0			x				x
1835	232744_x_at	ATXN1	chr6p23	0			x				x
1836	212016_s_at	PTBP1	chr19p13.3	0	x						x
1837	204790_at	SMAD7	chr18q21.1	0	x	x					
1838	208800_at	SRP72	chr4q11	0	x	x					
1839	239228_at	CSNK2A1	chr20p13	0			x				
1840	225265_at	RBMS1 /// C2(	chr2q24.2 /// chr2q24	0			x				

1841	1557689_at	NPAS2	chr2q11.2	0					x	
1842	218039_at	NUSAP1	chr15q15.1	0	x					
1843	243648_at	---	---	0			x			x
1844	244010_at	ABP1	chr7q34-q36	0			x			
1845	211084_x_at	PRKD3	chr2p21	0	x					x
1846	204969_s_at	RDX	chr11q23	0	x					
1847	228723_at	NPTN	chr15q22	0			x			
1848	202388_at	RGS2	chr1q31	0	x					x
1849	203729_at	EMP3	chr19q13.3	0	x	x				
1850	226282_at	---	---	0			x			x
1851	209908_s_at	TGFB2	chr1q41	0	x					
1852	214844_s_at	DOK5	chr20q13.2	0	x					
1853	239243_at	ZNF638	chr2p13.2-p13.1	0			x			
1854	204036_at	EDG2	chr9q31.3	0	x					
1855	203232_s_at	ATXN1	chr6p23	0	x					x
1856	228776_at	GJA7	chr17q21.31	0			x	x		
1857	230744_at	---	---	0			x			x
1858	201476_s_at	RRM1	chr11p15.5	0	x					x
1859	212428_at	KIAA0368	chr9q31.3	0	x					
1860	208999_at	38968	chr5q31	0	x					x
1861	202733_at	P4HA2	chr5q31	0	x	x				
1862	209293_x_at	ID4	chr6p22-p21	0	x					
1863	205687_at	UBPH	chr16p12	0	x					
1864	201926_s_at	CD55	chr1q32	0	x					
1865	240458_at	ITPR2	chr12p11	0			x			
1866	213807_x_at	MET	chr7q31	0	x					x
1867	218499_at	RP6-213H19.1	chrXq26.2	0	x	x				x
1868	227709_at	---	---	0			x			x
1869	219270_at	CHAC1	chr15q15.1	0	x					x
1870	1554015_a_at	CHD2	chr15q26	0					x	
1871	210568_s_at	RECQL	chr12p12	0	x					x
1872	210210_at	MPZL1	chr1q24.2	0	x					x
1873	218409_s_at	DNAJC1	chr10p12.31	0	x					
1874	240128_at	NT5C3	chr7p14.3	0			x			
1875	234942_s_at	DNTTIP1	chr20q13.12	0			x			
1876	1560271_at	PTBP2	chr1p22.1-p21.3	0					x	
1877	210559_s_at	CDC2	chr10q21.1	0	x					x
1878	228121_at	TGFB2	chr1q41	0			x	x		
1879	225220_at	SNORA24	chr4q26	0			x			
1880	238994_at	ZA20D1	chr1q21.2	0			x			
1881	233555_s_at	SULF2	chr20q12-q13.2	0			x			
1882	218943_s_at	DDX58	chr9p12	0	x					
1883	221972_s_at	SDF4	chr1p36.33	0	x					
1884	209028_s_at	ABI1	chr10p11.2	0	x	x				x
1885	212977_at	CMKOR1	chr2q37.3	0	x	x				
1886	202431_s_at	MYC	chr8q24.12-q24.13	0	x					
1887	214318_s_at	FRY	chr13q13.1	0	x					
1888	209306_s_at	SWAP70	chr11p15	0	x					
1889	31845_at	ELF4	chrXq26	0	x					
1890	217730_at	TMBIM1	chr2p24.3-p24.1	0	x					x

1891	235299_at	---	---	0			x						x
1892	204078_at	SC65	chr17q21.2	0	x								
1893	238151_at	TUBB6	chr18p11.21	0			x						x
1894	232231_at	RUNX2	chr6p21	0			x	x					
1895	207121_s_at	MAPK6	chr15q21	0	x								
1896	240165_at	---	---	0			x						x
1897	239954_at	ZNF160	chr19q13.41	0			x	x	x				
1898	204678_s_at	KCNK1	chr1q42-q43	0	x								
1899	225612_s_at	B3GNT5	chr3q28	0			x	x					x
1900	200776_s_at	BZW1 /// LOC	chr2q33 /// chr3q13.3	0	x								
1901	243496_at	---	---	0			x						x
1902	209305_s_at	GADD45B	chr19p13.3	0	x								x
1903	206668_s_at	SCAMP1	chr5q13.3-q14.1	0	x								x
1904	225201_s_at	MRPL14	chr6p21.3	0			x						
1905	228218_at	---	---	0			x	x					x
1906	1556543_at	ZCCHC7	chr9p13.2	0						x	x	x	
1907	823_at	CX3CL1	chr16q13	0	x								
1908	220273_at	IL17B	chr5q32-34	0	x								
1909	221905_at	CYLD	chr16q12.1	0	x								
1910	203140_at	BCL6	chr3q27	0	x								x
1911	201107_s_at	THBS1	chr15q15	0	x								x
1912	202539_s_at	HMGCR	chr5q13.3-q14	0	x								
1913	225384_at	DOCK7	chr1p31.3	0			x						
1914	238736_at	REV3L	chr6q21	0			x						x
1915	209011_at	TRIO	chr5p15.1-p14	0	x								
1916	201109_s_at	THBS1	chr15q15	0	x	x							x
1917	210178_x_at	FUSIP1 /// LO	chr1p36.11	0	x								
1918	204369_at	PIK3CA	chr3q26.3	0	x								
1919	201993_x_at	HNRPDL	chr4q13-q21	0	x								
1920	204881_s_at	UGCG	chr9q31	0	x								
1921	225739_at	RAB11FIP4	---	0			x						
1922	238156_at	RPS6	chr9p21	0			x	x	x				x
1923	210655_s_at	FOXO3A	chr6q21	0	x								x
1924	207002_s_at	PLAGL1	chr6q24-q25	0	x								x
1925	1555920_at	CBX3	chr7p15.2	0						x			x
1926	227646_at	---	---	0			x						x
1927	207643_s_at	TNFRSF1A	chr12p13.2	0	x								x
1928	225516_at	SLC7A2	chr8p22-p21.3	0			x	x					
1929	232542_at	COL9A2	chr1p33-p32	0			x	x	x				x
1930	225106_s_at	OGFOD1	chr16q13	0			x						
1931	203484_at	SEC61G	chr7p11.2	0	x								
1932	231880_at	FAM40B	chr7q32.1	0			x						
1933	1554678_s_at	HNRPDL	chr4q13-q21	0						x			
1934	227481_at	CNKSR3	chr6q25.2	0			x	x					x
1935	210875_s_at	TCF8	chr10p11.2	0	x								
1936	212444_at	---	---	0	x	x							x
1937	204194_at	BACH1	chr21q22.11	0	x								
1938	218847_at	IGF2BP2	chr3q27.2	0	x	x							x
1939	224744_at	IMPAD1	chr8q12.1	0			x						x
1940	218020_s_at	ZFAND3	chr6pter-p22.3	0	x								x

1941	203065_s_at	CAV1	chr7q31.1	0	x	x					x
1942	209803_s_at	PHLDA2	chr11p15.5	0	x						x
1943	228330_at	C6orf113	chr6q22.1	0			x				x
1944	221009_s_at	ANGPTL4	chr19p13.3	0	x						
1945	222034_at	GNB2L1	chr5q35.3	0	x						x
1946	203597_s_at	WBP4	chr13q14.11	0	x						
1947	229019_at	ZNF533	chr2q31.2-q31.3	0			x				
1948	204440_at	CD83	chr6p23	0	x						
1949	244811_at	IRAK1BP1	chr6q14-q15	0			x				
1950	236696_at	SR140	chr3q23	0			x				
1951	218880_at	FOSL2	chr2p23.3	0	x						
1952	216511_s_at	TCF7L2	chr10q25.3	0	x						x
1953	227561_at	LOC651619	---	0			x	x			
1954	234660_s_at	KIAA1008	chr13q22.1	0			x				x
1955	226051_at	SELM	chr22q12.2	0			x				
1956	226077_at	FLJ31951	chr5q33.3	0			x				
1957	229083_at	HNRPA0	chr5q31	0			x				
1958	225468_at	FLJ36874	chr11q12.1	0			x				x
1959	218104_at	TEX10	chr9q31.1	0	x						x
1960	235000_at	---	---	0			x				x
1961	223027_at	SNX9	chr6q25.1-q26	0			x				x
1962	222722_at	OGN	chr9q22	0			x	x			x
1963	226907_at	PPP1R14C	chr6q24.3-q25.3	0			x	x			
1964	222532_at	SRPRB	chr3q22.1	0			x				
1965	1555950_a_at	CD55	chr1q32	0					x	x	
1966	207526_s_at	IL1RL1	chr2q12	0	x	x					
1967	202067_s_at	LDLR	chr19p13.3	0	x	x					
1968	240926_at	LRCH3	chr3q29	0			x				
1969	219522_at	FJX1	chr11p13	0	x						
1970	235242_at	---	---	0			x				x
1971	241905_at	PIK3C2A	chr11p15.5-p14	0			x				x
1972	201309_x_at	C5orf13	chr5q22.1	0	x	x					x
1973	213136_at	PTPN2	chr18p11.3-p11.2	0	x						x
1974	210762_s_at	DLC1	chr8p22	0	x						x
1975	228790_at	C8orf72	chr8q12.1	0			x				
1976	219918_s_at	ASPM	chr1q31	0	x						x
1977	205572_at	ANGPT2	chr8p23.1	0	x						
1978	243631_at	LOC642333	chr12p13.31	0			x				
1979	1557918_s_at	SLC16A1	chr1p12	0					x	x	
1980	208066_s_at	GTF2B	chr1p22-p21	0	x						
1981	228105_at	SAPS3	chr11q13	0			x				
1982	226117_at	TIFA	chr4q25	0			x	x			x
1983	235959_at	ARID4B	chr1q42.1-q43	0			x				
1984	212542_s_at	PHIP	chr6q14	0	x						
1985	204622_x_at	NR4A2	chr2q22-q23	0	x						
1986	244000_at	DARS	chr2q21.3	0			x				x
1987	226795_at	LRCH1	chr13q14.13-q14.2	0			x				x
1988	212220_at	PSME4	chr2p16.2	0	x						
1989	225946_at	RASSF8	chr12p12.3	0			x	x			
1990	207265_s_at	KDELR3	chr22q13.1	0	x	x					

1991	219532_at	ELOVL4	chr6q14	0	x							
1992	1568954_s_at	C16orf72	chr16p13.2	0						x	x	
1993	222601_at	UBE1L2	chr4q13.2	0			x					
1994	229899_s_at	HSUP1	chr20q13.13	0			x	x	x			
1995	215716_s_at	ATP2B1	chr12q21.3	0	x							x
1996	217826_s_at	UBE2J1	chr6q15	0	x							x
1997	213353_at	ABCA5	chr17q24.3	0	x							
1998	204964_s_at	SSPN	chr12p11.2	0	x							
1999	200782_at	ANXA5	chr4q26-q28|4q28-q3	0	x							x
2000	236545_at	PPP3CA	chr4q21-q24	0			x	x				
2001	236402_at	---	---	0			x					x
2002	203234_at	UPP1	chr7p12.3	0	x							
2003	242438_at	---	---	0			x					x
2004	221019_s_at	COLEC12	chr18pter-p11.3	0	x							
2005	210235_s_at	PPFIA1	chr11q13.3	0	x							x
2006	227458_at	---	---	0			x	x				x
2007	202557_at	STCH	chr21q11.1|21q11	0	x							x
2008	232017_at	TJP2	chr9q13-q21	0			x					x
2009	202234_s_at	SLC16A1	chr1p12	0	x							
2010	201625_s_at	INSIG1	chr7q36	0	x							x
2011	229491_at	LOC133308	chr4q24	0			x					
2012	222600_s_at	UBE1L2	chr4q13.2	0			x					
2013	213260_at	---	---	0	x							x
2014	210372_s_at	TPD52L1	chr6q22-q23	0	x							x
2015	231243_s_at	BHLHB3	chr12p11.23-p12.1	0			x					
2016	212971_at	CARS	chr11p15.5	0	x							x
2017	1552867_at	---	---	0						x		x
2018	202403_s_at	COL1A2	chr7q22.1	0	x							x
2019	214321_at	NOV	chr8q24.1	0	x							
2020	218258_at	POLR1D	chr13q12.2	0	x							
2021	208228_s_at	FGFR2	chr10q26	0	x							x
2022	224654_at	DDX21	chr10q21	0			x					
2023	218156_s_at	TSR1	chr17p13.3	0	x							
2024	236322_at	FLJ31951	chr5q33.3	0			x					
2025	213342_at	YAP1	chr11q13	0	x							
2026	203893_at	TAF9	chr5q11.2-q13.1	0	x							
2027	1553959_a_at	B3GALT6	chr1p36.33	0						x		
2028	208801_at	SRP72	chr4q11	0	x							
2029	209360_s_at	RUNX1	chr21q22.3	0	x							x
2030	235020_at	TAF4B	chr18q11.2	0			x					
2031	203510_at	MET	chr7q31	0	x	x						x
2032	201305_x_at	ANP32B	chr9q22.32	0	x							
2033	219421_at	OSRF	chr5p15.2-p12	0	x							
2034	201116_s_at	CPE	chr4q32.3	0	x							x
2035	212107_s_at	DHX9	chr1q25	0	x							x
2036	238478_at	BNC2	chr9p22.3-p22.2	0			x					
2037	201846_s_at	RYBP	chr3p13	0	x							x
2038	209446_s_at	C7orf44	chr7p13	0	x							
2039	216235_s_at	EDNRA	chr4q31.23	0	x							
2040	200917_s_at	SRPR	chr11q24.3	0	x							x

2041	213901_x_at	RBM9	chr22q13.1	0	x					x
2042	1556416_s_at	SEC31L1	chr4q21.22	0					x	x
2043	218502_s_at	TRPS1	chr8q24.12	0	x					x
2044	204224_s_at	GCH1	chr14q22.1-q22.2	0	x	x				
2045	224720_at	MIB1	chr18q11.2	0			x			
2046	242943_at	ST8SIA4	chr5q21	0			x			
2047	227474_at	PAX8	chr2q12-q14	0			x			x
2048	201329_s_at	ETS2	chr21q22.3|21q22.2	0	x					x
2049	201888_s_at	IL13RA1	chrXq24	0	x					x
2050	205193_at	MAFF	chr22q13.1	0	x					x
2051	201263_at	TARS	chr5p13.2	0	x					x
2052	215167_at	CRSP2	chrXp11.4-p11.2	0	x					
2053	202686_s_at	AXL	chr19q13.1	0	x	x				
2054	202006_at	PTPN12	chr7q11.23	0	x					
2055	225195_at	ZCSL2	chr3p24.3	0			x			
2056	227260_at	ANKRD10	chr13q34	0			x			
2057	204252_at	CDK2 /// BCD(	chr12q13 /// chr11q2	0	x					
2058	210092_at	MAGOH	chr1p34-p33	0	x					
2059	228812_at	---	---	0			x	x		x
2060	213327_s_at	USP12	chr13q12.13	0	x					
2061	209287_s_at	CDC42EP3	chr2p21	0	x					x
2062	211016_x_at	HSPA4	chr5q31.1-q31.2	0	x					
2063	219280_at	BRWD1	chr21q22.2	0	x					
2064	213021_at	GOSR1	chr17q11	0	x					
2065	210018_x_at	MALT1	chr18q21	0	x					
2066	228603_at	LOC440900	chr2q14.1	0			x			
2067	226732_at	RBM33	chr7q36.3	0			x			
2068	205047_s_at	ASNS	chr7q21.3	0	x					x
2069	218686_s_at	RHBDF1	chr16p13.3	0	x					
2070	223416_at	SF3B14	chr2pter-p25.1	0			x			
2071	202647_s_at	NRAS	chr1p13.2	0	x					
2072	239144_at	---	---	0			x			x
2073	213001_at	ANGPTL2	chr9q34	0	x					x
2074	201666_at	TIMP1	chrXp11.3-p11.23	0	x					x
2075	201844_s_at	RYBP	chr3p13	0	x					x
2076	229398_at	RAB18	chr10p12.1	0			x			
2077	236114_at	RUNX1	chr21q22.3	0			x			x
2078	221873_at	ZNF143	chr11p15.4	0	x					
2079	211653_x_at	AKR1C2	chr10p15-p14	0	x					
2080	238496_at	WHSC1L1	chr8p11.2	0			x			x
2081	211450_s_at	MSH6	chr2p16	0	x					x
2082	201662_s_at	ACSL3	chr2q34-q35	0	x					x
2083	207983_s_at	STAG2	chrXq25	0	x					
2084	226926_at	DMKN	chr19q13.12	0			x			
2085	207108_s_at	NIPBL	chr5p13.2	0	x					
2086	213164_at	SLC5A3	chr21q22.12	0	x	x				
2087	201180_s_at	GNAI3	chr1p13	0	x					
2088	215177_s_at	ITGA6	chr2q31.1	0	x					
2089	209034_at	PNRC1	chr6q15	0	x					x
2090	204175_at	ZNF593	chr1p36.11	0	x					

2091	221479_s_at	BNIP3L	chr8p21	0	x						x
2092	233365_at	PRKG1	chr10q11.2	0			x				
2093	203358_s_at	EZH2	chr7q35-q36	0	x						
2094	230710_at	---	---	0			x				x
2095	209373_at	MALL	chr2q13	0	x						x
2096	201446_s_at	TIA1	chr2p13	0	x						
2097	204351_at	S100P	chr4p16	0	x						
2098	218149_s_at	ZNF395	chr8p21.1	0	x						
2099	208092_s_at	FAM49A	chr2p24.3	0	x						
2100	222815_at	RNF12	chrXq13-q21	0			x				
2101	205436_s_at	H2AFX	chr11q23.2-q23.3	0	x						x
2102	201852_x_at	COL3A1	chr2q31	0	x	x					
2103	239619_at	ZNF395	chr8p21.1	0			x				
2104	202734_at	TRIP10	chr19p13.3	0	x						
2105	227361_at	---	---	0			x	x			x
2106	242059_at	ETNK1	chr12p12.1	0			x				
2107	221007_s_at	FIP1L1	chr4q12	0	x						
2108	227180_at	ELOVL7	chr5q12.1	0			x	x			
2109	222395_s_at	UBE2Z	chr17q21.32	0			x				
2110	211948_x_at	BAT2D1	chr1q23.3	0	x						x
2111	205486_at	TESK2	chr1p32	0	x						
2112	1552381_at	USP32 /// SRr	chr17q23.2 /// chr6q1	0					x		
2113	222634_s_at	TBL1XR1	chr3q26.32	0			x				
2114	212599_at	AUTS2	chr7q11.22	0	x						
2115	202376_at	SERPINA3	chr14q32.1	0	x	x					x
2116	226107_at	---	---	0			x				x
2117	1554768_a_at	MAD2L1	chr4q27	0					x	x	
2118	224252_s_at	FXYD5	chr19q12-q13.1	0			x				x
2119	208864_s_at	TXN	chr9q31	0	x						
2120	202421_at	IGSF3	chr1p13	0	x						
2121	225043_at	SLC15A4	chr12q24.32	0			x				
2122	218371_s_at	PSPC1	chr13q12.11	0	x						
2123	225018_at	SPIRE1	chr18p11.21	0			x				x
2124	225606_at	BCL2L11	chr2q13	0			x				
2125	201510_at	ELF3	chr1q32.2	0	x						
2126	214720_x_at	38970	chr2q13	0	x						x
2127	218009_s_at	PRC1	chr15q26.1	0	x						x
2128	233878_s_at	XRN2	chr20p11.2-p11.1	0			x				
2129	210220_at	FZD2	chr17q21.1	0	x						
2130	204955_at	SRPX	chrXp21.1	0	x						x
2131	232032_x_at	SDF4	chr1p36.33	0			x				
2132	219469_at	DYNC2H1	chr11q21-q22.1	0	x						
2133	1554691_a_at	PACSIN2	chr22q13.2-13.33	0					x		
2134	241425_at	NUPL1	chr13q12.13	0			x				x
2135	204188_s_at	RARG	chr12q13	0	x						x
2136	226814_at	ADAMTS9	chr3p14.3-p14.2	0			x				
2137	209243_s_at	PEG3	chr19q13.4	0	x						x
2138	207276_at	CDR1	chrXq27.1-q27.2	0	x						x
2139	238988_at	TOP1	chr20q12-q13.1	0			x				
2140	201914_s_at	SEC63	chr6q21	0	x						x

2141	201514_s_at	G3BP	chr5q33.1	0	x					x
2142	218268_at	TBC1D15	chr12q21.1	0	x					
2143	1556551_s_at	SLC39A6	chr18q12.2	0				x	x	
2144	216971_s_at	PLEC1	chr8q24	0	x					
2145	201426_s_at	VIM	chr10p13	0	x					x
2146	210337_s_at	ACLY	chr17q12-q21	0	x					
2147	202409_at	IGF2 /// INS-IG	chr11p15.5	0	x	x				x
2148	215931_s_at	RARA /// ARF(	chr17q21 /// chr20q1	0	x					
2149	222393_s_at	NAT13	chr3q13.2	0			x			
2150	224796_at	DDEF1	chr8q24.1-q24.2	0			x			
2151	201412_at	LRP10	chr14q11.2	0	x					
2152	230229_at	DLG1	chr3q29	0			x			x
2153	221803_s_at	NRBF2	chr10q21.2	0	x					
2154	212930_at	ATP2B1	chr12q21.3	0	x	x				x
2155	201861_s_at	LRRFIP1	chr2q37.3	0	x					x
2156	229011_at	---	---	0			x			x
2157	238651_at	CPSF6	chr12q15	0			x			x
2158	209149_s_at	TM9SF1	chr14q11.2	0	x					
2159	201506_at	TGFBI	chr5q31	0	x					
2160	200751_s_at	HNRPC	chr14q11.2	0	x					x
2161	212522_at	PDE8A	chr15q25.3	0	x					
2162	226925_at	ACPL2	chr3q23	0			x			x
2163	205021_s_at	CHES1	chr14q31.3	0	x					x
2164	200596_s_at	EIF3S10	chr10q26	0	x					x
2165	205273_s_at	PITRM1	chr10p15.2	0	x					x
2166	213418_at	HSPA6	chr1q23	0	x					x
2167	213943_at	TWIST1	chr7p21.2	0	x					
2168	226911_at	EGFLAM	chr5p13.2-p13.1	0			x			
2169	222427_s_at	LARS	chr5q32	0			x			x
2170	219064_at	ITIH5	chr10p14	0	x	x				
2171	217805_at	ILF3	chr19p13.2	0	x					x
2172	227252_at	LRP10	chr14q11.2	0			x			
2173	203736_s_at	PPFIBP1	chr12p11.23-p11.22	0	x					x
2174	235566_at	---	---	0			x			x
2175	243338_at	CSNK1A1	chr5q32	0			x			x
2176	1554433_a_at	ZNF146	chr19q13.1	0				x		x
2177	210756_s_at	NOTCH2	chr1p13-p11	0	x					
2178	1555411_a_at	CCNL1	chr3q25.32	0				x	x	
2179	1560332_at	CHST11	chr12q	0				x		x
2180	210973_s_at	FGFR1	chr8p11.2-p11.1	0	x					
2181	219249_s_at	FKBP10	chr17q21.2	0	x	x				
2182	231904_at	U2AF1	chr21q22.3	0			x			x
2183	221039_s_at	DDEF1	chr8q24.1-q24.2	0	x					
2184	1556364_at	---	---	0				x	x	x
2185	200712_s_at	MAPRE1	chr20q11.1-11.23	0	x					x
2186	200598_s_at	HSP90B1	chr12q24.2-q24.3	0	x					
2187	210978_s_at	TAGLN2	chr1q21-q25	0	x					x
2188	236808_at	FGFR1OP2	chr12p11.23	0			x			
2189	203820_s_at	IGF2BP3	chr7p11	0	x					
2190	234994_at	KIAA1913	chr6q23.1	0			x			

2191	235493_at	ZNF638	chr2p13.2-p13.1	0			x				
2192	213024_at	TMF1	chr3p21-p12	0	x						
2193	215888_at	APRIN	chr13q12.3	0	x						
2194	200885_at	RHOC	chr1p13.1	0	x						x
2195	225240_s_at	MSI2	chr17q22	0			x	x			
2196	212814_at	KIAA0828	chr7q32.1	0	x						
2197	201231_s_at	ENO1	chr1p36.3-p36.2	0	x						x
2198	244026_at	ELL2	chr5q15	0			x	x			x
2199	203244_at	PEX5	chr12p13.3	0	x						
2200	226982_at	ELL2	chr5q15	0			x	x			x
2201	202843_at	DNAJB9	chr7q31|14q24.2-q24	0	x						x
2202	201195_s_at	SLC7A5	chr16q24.3	0	x						
2203	1554600_s_at	LMNA	chr1q21.2-q21.3	0					x	x	x
2204	201887_at	IL13RA1	chrXq24	0	x						x
2205	201948_at	GNL2	chr1p34.3	0	x						
2206	225866_at	BXDC1	chr6q21	0			x				
2207	203491_s_at	CEP57	chr11q21	0	x						
2208	217988_at	CCNB1IP1	chr14q11.2	0	x						x
2209	202856_s_at	SLC16A3	chr17q25	0	x						
2210	215646_s_at	CSPG2	chr5q14.3	0	x						x
2211	228238_at	GAS5	chr1q25.1	0			x				x
2212	221781_s_at	DNAJC10	chr2q32.1	0	x						
2213	204975_at	EMP2	chr16p13.2	0	x	x					
2214	225237_s_at	MSI2	chr17q22	0			x				
2215	201291_s_at	TOP2A	chr17q21-q22	0	x						
2216	215076_s_at	COL3A1	chr2q31	0	x	x					
2217	204268_at	S100A2	chr1q21	0	x						
2218	225189_s_at	RAPH1	chr2q33	0			x	x			x
2219	223171_at	DYM	chr18q12-q21.1	0			x				
2220	225897_at	---	---	0			x				x
2221	213661_at	DKFZP586H2	chr11p13	0	x						x
2222	235074_at	SPRED1	chr15q14	0			x				
2223	207977_s_at	DPT	chr1q12-q23	0	x						
2224	225539_at	ZNF295	chr21q22.3	0			x				
2225	221677_s_at	DONSON	chr21q22.1	0	x						
2226	202437_s_at	CYP1B1	chr2p21	0	x	x					x
2227	224852_at	TTC17	chr11p12-p11.2	0			x				
2228	214741_at	ZNF131	chr5p12-p11	0	x						x
2229	209669_s_at	SERBP1	chr1p31	0	x						x
2230	213986_s_at	C19orf6	chr19p13.3	0	x						x
2231	228563_at	GJA7	chr17q21.31	0			x				
2232	235542_at	MGC22014	chr2p13.1	0			x				
2233	225264_at	RARSL	chr6q16.1	0			x				
2234	217728_at	S100A6	chr1q21	0	x						
2235	204731_at	TGFBR3	chr1p33-p32	0	x						
2236	219087_at	ASPN	chr9q22	0	x	x					
2237	202822_at	LPP	chr3q28	0	x						
2238	202721_s_at	GFPT1	chr2p13	0	x						
2239	221489_s_at	SPRY4 /// LOC	chr5q31.3	0	x						x
2240	206211_at	SELE	chr1q22-q25	0	x	x					

2241	212065_s_at	THRB /// USP3	chr3p24.2 /// chr2p15	0	x							
2242	226771_at	ATP8B2	chr1q21.3	0			x					
2243	228442_at	---	---	0			x	x				x
2244	228201_at	ARL13B	chr3q11.2	0			x					
2245	202609_at	EPS8	chr12q13	0	x							x
2246	226000_at	CTTNBP2NL	chr1p13.2	0			x					
2247	218036_x_at	NMD3	chr3q26.1	0	x							
2248	201101_s_at	BCLAF1	chr6q22-q23	0	x							
2249	204825_at	MELK	chr9p13.2	0	x							
2250	202531_at	IRF1	chr5q31.1	0	x							x
2251	232216_at	YME1L1	chr10p14	0			x	x	x			x
2252	222435_s_at	UBE2J1	chr6q15	0			x					x
2253	201211_s_at	DDX3X	chrXp11.3-p11.23	0	x							
2254	212110_at	SLC39A14	chr8p21.3	0	x	x						x
2255	217716_s_at	SEC61A1	chr3q21.3	0	x							x
2256	228843_at	---	---	0			x					x
2257	230462_at	NUMB	chr14q24.3	0			x					x
2258	210426_x_at	RORA	chr15q21-q22	0	x							
2259	235743_at	SNED1	chr2q37.3	0			x					
2260	218516_s_at	IMPAD1	chr8q12.1	0	x							x
2261	218696_at	EIF2AK3	chr2p12	0	x							
2262	209162_s_at	PRPF4	chr9q31-q33	0	x							x
2263	233952_s_at	ZNF295	chr21q22.3	0			x					
2264	210827_s_at	ELF3	chr1q32.2	0	x							
2265	37152_at	PPARD	chr6p21.2-p21.1	0	x							x
2266	239804_at	CCNI	chr4q21.1	0			x					x
2267	200869_at	RPL18A /// LO	chr19p13 /// chr12q2	0	x							
2268	204761_at	USP6NL	chr10p13	0	x							
2269	201394_s_at	RBM5	chr3p21.3	0	x							x
2270	1555630_a_at	RAB34	chr17q11.2	0						x	x	
2271	201014_s_at	PAICS	chr4pter-q21	0	x							
2272	225569_at	EIF2C2	chr8q24	0			x					x
2273	217591_at	SKIL	chr3q26	0	x							
2274	230494_at	---	---	0			x					x
2275	211573_x_at	TGM2	chr20q12	0	x							
2276	212201_at	KIAA0692	chr12q24.33	0	x							x
2277	213094_at	GPR126	chr6q24.1	0	x	x						
2278	205281_s_at	PIGA	chrXp22.1	0	x							
2279	223297_at	MGC4268	chr2q21	0			x					
2280	227517_s_at	C1orf155	chr1q25.1	0			x					
2281	160020_at	MMP14	chr14q11-q12	0	x							
2282	214908_s_at	TRRAP	chr7q21.2-q22.1	0	x							
2283	201325_s_at	EMP1	chr12p12.3	0	x	x						x
2284	201663_s_at	SMC4	chr3q26.1	0	x							
2285	201559_s_at	CLIC4	chr1p36.11	0	x	x						x
2286	224764_at	ARHGAP21	chr10p12.1|10p12.3	0			x					
2287	222385_x_at	SEC61A1	chr3q21.3	0			x					x
2288	201635_s_at	FXR1	chr3q28	0	x							
2289	224973_at	FAM46A	chr6q14	0			x	x				x
2290	242990_at	---	---	0			x					x

2291	235457_at	MAML2	chr11q21	0			x				
2292	222547_at	MAP4K4	chr2q11.2-q12	0			x				
2293	216614_at	ITPR2	chr12p11	0	x						
2294	211744_s_at	CD58	chr1p13	0	x						
2295	1558214_s_at	CTNNA1	chr5q31	0					x	x	x
2296	230375_at	C6orf111	chr6q16.3	0			x				
2297	208683_at	CAPN2	chr1q41-q42	0	x						x
2298	210139_s_at	PMP22	chr17p12-p11.2	0	x						x
2299	227870_at	NOPE	chr15q22.31	0			x				
2300	232898_at	DAB2	chr5p13	0			x				
2301	212232_at	FNBP4	chr11p11.2	0	x						x
2302	200968_s_at	PPIB	chr15q21-q22	0	x						
2303	202897_at	SIRPA	chr20p13	0	x						x
2304	242055_at	C6orf85	chr6p25.2	0			x				
2305	218611_at	IER5	chr1q25.3	0	x						x
2306	211955_at	RANBP5	chr13q32.2	0	x						
2307	225078_at	EMP2	chr16p13.2	0			x				
2308	213548_s_at	CDV3	chr3q22.1	0	x						
2309	203355_s_at	PSD3	chr8pter-p23.3	0	x	x					x
2310	204559_s_at	LSM7	chr19p13.3	0	x						
2311	202627_s_at	SERPINE1	chr7q21.3-q22	0	x	x					x
2312	218423_x_at	VPS54	chr2p13-p14	0	x						
2313	225443_at	DCP1A	chr3p21.1	0			x				
2314	212503_s_at	DIP2C	chr10p15.3	0	x						
2315	209295_at	TNFRSF10B	chr8p22-p21	0	x						x
2316	1558569_at	LOC645238	chr11q21	0					x	x	
2317	210983_s_at	MCM7	chr7q21.3-q22.1	0	x						
2318	222623_s_at	ZNF639	chr3q26.32	0			x				
2319	218277_s_at	DHX40	chr17q23.1	0	x						
2320	235494_at	---	---	0			x				x
2321	1553349_at	ARID2	chr12q12	0					x	x	
2322	215294_s_at	SMARCA1	chrXq25	0	x						
2323	1565886_at	---	---	0					x		x
2324	224714_at	MKI67IP	chr2q14.3	0			x				
2325	203138_at	HAT1	chr2q31.2-q33.1	0	x						
2326	212529_at	LSM12 /// LOC	chr17q21.31 /// chr8p	0	x						
2327	204017_at	KDELR3	chr22q13.1	0	x						
2328	202697_at	NUDT21	chr16q13	0	x						
2329	201872_s_at	ABCE1	chr4q31	0	x						
2330	203136_at	RABAC1	chr19q13.2	0	x						
2331	225842_at	PHLDA1	chr12q15	0			x				x
2332	221881_s_at	ADD3 /// CLIC	chr10q24.2-q24.3 ///	0	x						x
2333	221922_at	GPSM2	chr1p13.3	0	x						x
2334	206245_s_at	IVNS1ABP	chr1q25.1-q31.1	0	x						
2335	1553704_x_at	ZNF791	chr19p13.2-p13.13	0					x		
2336	218667_at	PJA1	chrXq13.1	0	x						x
2337	202357_s_at	CFB	chr6p21.3	0	x						
2338	203083_at	THBS2	chr6q27	0	x						
2339	210137_s_at	DCTD	chr4q35.1	0	x						x
2340	244623_at	---	---	0			x	x			x

2341	226650_at	ZFAND2A	chr7p22.3	0			x			x
2342	235333_at	B4GALT6	chr18q11	0			x			
2343	225498_at	CHMP4B	chr20q11.22	0			x			
2344	223122_s_at	SFRP2	chr4q31.3	0			x			
2345	209774_x_at	CXCL2	chr4q21	0	x	x				x
2346	243829_at	BRAF	chr7q34	0			x			
2347	216942_s_at	CD58	chr1p13	0	x					
2348	215780_s_at	SET /// LOC64	chr9q34 /// chrXq21.	0	x					
2349	226963_at	BTF3L4	chr1p32.3	0			x			
2350	214790_at	SENP6	chr6q13-q14.3	0	x					x
2351	221766_s_at	FAM46A	chr6q14	0	x	x				x
2352	212813_at	JAM3	chr11q25	0	x					x
2353	242405_at	MAML2	chr11q21	0			x			
2354	201772_at	AZIN1	chr8q22.3	0	x					x
2355	200756_x_at	CALU	chr7q32	0	x					x
2356	209834_at	CHST3	chr10q22.1	0	x	x				
2357	225834_at	LOC652689 ///	chr1q32.1 /// chr1q21	0			x			
2358	222416_at	ALDH18A1	chr10q24.3	0			x			x
2359	223234_at	MAD2L2	chr1p36	0			x			
2360	243908_at	ZNF638	chr2p13.2-p13.1	0			x			
2361	236404_at	ATXN1	chr6p23	0			x			x
2362	233595_at	USP34	chr2p15	0			x			
2363	212561_at	RAB6IP1	chr11p15.4	0	x					
2364	203357_s_at	CAPN7	chr3p24	0	x					
2365	202816_s_at	SS18	chr18q11.2	0	x					
2366	226335_at	RPS6KA3	chrXp22.2-p22.1	0			x			
2367	233824_at	LPP	chr3q28	0			x			
2368	205931_s_at	CREB5	chr7p15.1	0	x					
2369	226722_at	FAM20C	chr7p22.3	0			x			
2370	203973_s_at	CEBPD	chr8p11.2-p11.1	0	x					x
2371	235022_at	C18orf19	chr18p11.21	0			x			
2372	202598_at	S100A13	chr1q21	0	x					
2373	209209_s_at	PLEKHC1	chr14q22.2	0	x	x				x
2374	236831_at	CCDC50	---	0			x			
2375	217788_s_at	GALNT2	chr1q41-q42	0	x					x
2376	200665_s_at	SPARC	chr5q31.3-q32	0	x					x
2377	203649_s_at	PLA2G2A	chr1p35	0	x					
2378	228284_at	TLE1	chr9q21.32	0			x			
2379	208916_at	SLC1A5	chr19q13.3	0	x					x
2380	242358_at	---	---	0			x			x
2381	202536_at	CHMP2B	chr3p11.2	0	x					x
2382	227070_at	GLT8D2	chr12q	0			x	x		x
2383	209043_at	PAPSS1	chr4q24	0	x					x
2384	200800_s_at	HSPA1A /// HS	chr6p21.3	0	x					
2385	218284_at	SMAD3	chr15q22.33	0	x					
2386	201626_at	INSIG1	chr7q36	0	x					x
2387	200755_s_at	CALU	chr7q32	0	x					x
2388	227148_at	PLEKHH2	chr2p21	0			x	x		
2389	208070_s_at	REV3L	chr6q21	0	x					x
2390	1557950_at	SDCCAG1	chr14q22	0					x	

2391	228635_at	PCDH10	chr4q28.3	0			x		
2392	222621_at	DNAJC1	chr10p12.31	0			x		
2393	213135_at	TIAM1	chr21q22.1|21q22.11	0	x				
2394	201818_at	AYTL2	chr5p15.33	0	x				
2395	223484_at	C15orf48	chr15q21.1	0			x	x	
2396	222171_s_at	PKNOX2	---	0	x				
2397	229244_at	---	---	0			x		x
2398	226705_at	FGFR1	chr8p11.2-p11.1	0			x		
2399	223125_s_at	C1orf21	chr1q25	0			x		
2400	218013_x_at	DCTN4	chr5q31-q32	0	x				
2401	201164_s_at	RNF6 /// PUM1	chr13q12.2 /// chr1p3	0	x				x
2402	226452_at	PDK1	chr2q31.1	0			x	x	
2403	200962_at	RPL31 /// LOC	chr2q11.2 /// chr2q36	0	x				
2404	219778_at	ZFPM2	chr8q23	0	x				
2405	213359_at	HNRPD	chr4q21.1-q21.2	0	x				x
2406	201136_at	PLP2	chrXp11.23	0	x				
2407	213462_at	NPAS2	chr2q11.2	0	x				
2408	214055_x_at	BAT2D1	chr1q23.3	0	x				x
2409	209056_s_at	CDC5L	chr6p21	0	x				x
2410	202729_s_at	LTBP1	chr2p22-p21	0	x				x
2411	212194_s_at	TM9SF4	chr20q11.21	0	x				
2412	240798_at	CUTL1	chr7q22.1	0			x		
2413	224937_at	PTGFRN	chr1p13.1	0			x	x	x
2414	209211_at	KLF5	chr13q22.1	0	x				x
2415	224642_at	FYTTD1	chr3q29	0			x		
2416	223993_s_at	CNIH4	chr1q42.11	0			x		
2417	200597_at	EIF3S10	chr10q26	0	x				x
2418	216321_s_at	NR3C1	chr5q31.3	0	x				x
2419	216048_s_at	RHOBTB3	chr5q15	0	x				x
2420	219049_at	ChGn	chr8p21.3	0	x	x			
2421	202620_s_at	PLOD2	chr3q23-q24	0	x	x			x
2422	206445_s_at	PRMT1	chr19q13.3	0	x				
2423	227236_at	TSPAN2	chr1p13.2	0			x		
2424	202192_s_at	GAS7	chr17p13.1	0	x				x
2425	219117_s_at	FKBP11	chr12q13.12	0	x				
2426	239561_at	THRAP2	chr12q24.21	0			x		
2427	226025_at	ANKRD28	chr3p24.3	0			x		x
2428	209218_at	SQLE	chr8q24.1	0	x				x
2429	200750_s_at	RAN	chr12q24.3	0	x				x
2430	218947_s_at	PAPD1	chr10p11.23	0	x				
2431	217196_s_at	CAMSAP1L1	chr1q32.1	0	x				
2432	232094_at	C15orf29	chr15q14	0			x		
2433	204645_at	CCNT2	chr2q21.3	0	x				
2434	202023_at	EFNA1	chr1q21-q22	0	x				
2435	201012_at	ANXA1	chr9q12-q21.2|9q12-	0	x				
2436	220917_s_at	WDR19	chr4p14	0	x				
2437	209013_x_at	TRIO	chr5p15.1-p14	0	x				
2438	211954_s_at	RANBP5	chr13q32.2	0	x				
2439	212915_at	PDZRN3	chr3p13	0	x				
2440	238520_at	---	---	0			x		x

2441	225799_at	MGC4677 /// L	chr2p11.2 /// chr2q13	0			x	x
2442	219338_s_at	LRRC49	chr15q23	0	x			
2443	219679_s_at	WAC	---	0	x			
2444	202589_at	TYMS	chr18p11.32	0	x			
2445	214167_s_at	RPLP0 /// RPL	chr12q24.2 /// chr2p2	0	x			
2446	227444_at	ARMCX4	chrXq22.1	0			x	
