# Supplementary material for: Cartilage-selective genes identified in genome-scale analysis of non-cartilage and cartilage gene expression
Source: BMC Genomics. 2007 Jun 12;8:165. doi: 10.1186/1471-2164-8-165 (PMC1906768; doi:10.1186/1471-2164-8-165)
Supplement: Additional File 3 — Tissue distribution training and validation sets. (A) 31 Non-cartilage tissues and 124 arrays were used for the validation of cartilage selective genes identified on the U133A chip. Two fetal cartilage samples were compared against 122 non-cartilage samples. (B) 27 non-cartilage Tissues and 74 arrays were used for the validation of cartilage selective genes identified on the U133B chip. Two fetal cartilage samples were compared against 72 non-cartilage arrays (C) Eight non-cartilage tissues and 26 arrays used for the validation of cartilage selective genes identified on the U133B chip. Five fetal cartilage samples were compared against 72 non-cartilage arrays. [file 1471-2164-8-165-S3.pdf]

Suplemental Table 2A. 31 Non-cartilage Tissues and 124 arrays were used for the validation of cartilage selective genes identified on the U133A chip. 2 Fetal Cartilage samples were compared against 122 non-cartilage samples.

Arrays	Tissues	Dissected tissues included in category[1,2]
32	Brain	(Including:Total, Fetal Cortex (2), Amygdala, Caudate Nucleus, Cerebellum, Cerebellar peduncles, Ciliary Ganglion, Dorsal root Ganglion, Cingulate Cortex, Globus pallidus, Hypothalamus, Medulla oblangata, Occipital lobe, Olfactory bulb, Parietal lobe, Pons, Prefrontal Cortex, Subthalamic nucleus, Superior Cervical Ganglion, Temporal Lobe, Thalamus, Trigeminal Ganglion, Aging Putamen, Aging White Matter, Left Cortex, Occipital Cortex, Parietal Cortex, Right Cortex)
8	Kidney	(Including:Total, Fetal (2), AdrenalCortex, Adrenal Gland)
8	Lung	(Including:Total, Fetal (4))
7	Blood	(Including:Total, Granulocyte, Macrophage, Monocyte)
6	Liver	(Including:Total, Fetal (2))
5	Uterus	(Including:Total, Corpus)
4	Pancreas	(Including:Total, Pancreatic Islet)
4	Placenta	
4	Thyroid	(Including:Total, Fetal (2))
3	Colon	(Including:Total, Mucusal lining)
3	Esophagus	
3	Ovary	
3	Prostate	
3	Salivary gland	
3	Skeletal Muscle	
3	Spleen	
3	Thymus	
2	Adipose	
2	Bone Marrow	
2	Heart	(Including:Total, Atrioventricular node)
2	Lymphnode/Tonsil	
2	Small Intestine	(Including: Total, Ileum)
2	Testis	
1	Appendix	
1	Breast	
1	Pituitary	
1	Skin	
1	Smooth Muscle	
1	Fetal Spinal Column	
1	Stomach	
1	Tongue	

[1]Blank if only total tissue was used.
[2]Number of fetal arrays are indicated in brackets

Supplemental Table 2B. 27 Non-cartilage Tissues and 74 arrays were used for the validation of cartilage selective genes identified on the U133B chip. 2 Fetal Cartilage samples were compared against 72 non-cartilage arrays.

Array	Tissues	Dissected tissues included in category[1,2]
11	Brain	(Including:Total, Fetal Cortex (1), Cerebellum, White Matter)
5	Kidney	(Including:Total, Fetal (3), Adrenal Gland, Ureter)
4	Bone Marrow	
4	Liver	
4	Lung	(including: Total, Fetal (2))
3	Ovary	
3	Skeletal Muscle	
3	Small Intestine	(Including:Total, Mucosa)
3	Spleen	
3	Stomach	
3	Thymus	
3	Thyroid	
3	Uterus	
2	Breast	
2	Colon	
2	Heart	
2	Placenta	
2	Prostate	
2	Testis	
1	Aorta	(Including:Fetal)
1	Bladder	(Including:Fetal)
1	Esophagus	
1	Lymph node	
1	Pancreas	
1	Salivary Gland	
1	Skin	
1	Fetal Spinal Column	

Supplemental Table 2C. 8 Non-cartilage Tissues and 26 arrays used for the validation of cartilage selective genes identified on the U133 Plus 2.0 chip. 5 Fetal Cartilage samples were compared against 72 non-cartilage arrays.

Arrays	Tissues	Dissected tissues included in category[1,2]
8	Brain	(Including:Total, Prefrontal Brain)
3	Kidney	
2	Liver	
2	Heart	
2	Testis	
2	Prostate	
1	Liver	
1	Spleen	
